# Wheat Landrace Genome Diversity

**DOI:** 10.1534/genetics.116.194688

**Published:** 2017-02-16

**Authors:** Luzie U. Wingen, Claire West, Michelle Leverington-Waite, Sarah Collier, Simon Orford, Richard Goram, Cai-Yun Yang, Julie King, Alexandra M. Allen, Amanda Burridge, Keith J. Edwards, Simon Griffiths

**Affiliations:** * Crop Genetics, John Innes Centre, Norwich NR4 7UH, UK; † Division of Plant and Crop Sciences, School of Biosciences, University of Nottingham, Sutton Bonington LE12 5RD, UK; ‡ Life Sciences, University of Bristol, BS8 1TQ, UK

**Keywords:** map distance, marker order, translocation, segregation distortion, recombination QTL, nested association mapping

## Abstract

Understanding the genomic complexity of bread wheat (*Triticum aestivum* L.) is a cornerstone in the quest to unravel the processes of domestication and the following adaptation of domesticated wheat to a wide variety of environments across the globe. Additionally, it is of importance for future improvement of the crop, particularly in the light of climate change. Focusing on the adaptation after domestication, a nested association mapping (NAM) panel of 60 segregating biparental populations was developed, mainly involving landrace accessions from the core set of the Watkins hexaploid wheat collection optimized for genetic diversity. A modern spring elite variety, “Paragon,” was used as common reference parent. Genetic maps were constructed following identical rules to make them comparable. In total, 1611 linkage groups were identified, based on recombination from an estimated 126,300 crossover events over the whole NAM panel. A consensus map, named landrace consensus map (LRC), was constructed and contained 2498 genetic loci. These newly developed genetics tools were used to investigate the rules underlying genome fluidity or rigidity, *e.g.*, by comparing marker distances and marker orders. In general, marker order was highly correlated, which provides support for strong synteny between bread wheat accessions. However, many exceptional cases of incongruent linkage groups and increased marker distances were also found. Segregation distortion was detected for many markers, sometimes as hot spots present in different populations. Furthermore, evidence for translocations in at least 36 of the maps was found. These translocations fell, in general, into many different translocation classes, but a few translocation classes were found in several accessions, the most frequent one being the well-known T5B:7B translocation. Loci involved in recombination rate, which is an interesting trait for plant breeding, were identified by QTL analyses using the crossover counts as a trait. In total, 114 significant QTL were detected, nearly half of them with increasing effect from the nonreference parents.

## Abstract (precis)

Understanding the genomic complexity of bread wheat is important for unraveling domestication processes, environmental adaptation, and for future of...

BREAD (hexaploid) wheat, also called common wheat, (*Triticum aestivum* L.) is oneof the “big three” cereal crops (shewry 2009). It is unrivalled in its geographic range of cultivation. Over 25,000 types of bread wheat have been developed in the process of adapting it to a wide range of environments (shewry 2009). The genomes of modern wheat varieties can be thought of as mosaics of landrace cultivars, which were chosen as the starting materials for systematic wheat breeding in the early 20th century. Much research has focused on the specific benefits that genes and alleles confer on the crop in attempting to explain genotypic outcomes of selection for any particular environment. However, it should be noted that other important factors influence the availability of beneficial alleles and allele combinations for selection. Chief among these are rates of recombination and segregation distortion.

Landrace collections in general show a much higher level of genetic diversity than elite varieties. There is a widely recognized imperative for breeding programs to use this genetic diversity by incorporating landrace-derived cultivars into these programs (moore 2015). The use is aided by the increased genotypic characterization of landrace collections such as those hosted at the Leibniz-Institute of Plant Genetics and Crop Plant Research (huang *et al.* 2002), INRA (balfourier *et al.* 2007), and the John Innes Centre (JIC) (wingen *et al.* 2014), and also of new collections, *e.g.*, of Mexican creole wheat (vikram *et al.* 2016). To achieve the incorporation of useful diversity, there is a need to comprehend the ramifications of the identified genetic variation on meiosis, sexual reproduction, and fertility in, *e.g.*, segregating populations. This will help us to understand the genetic events underpinning domestication and the geographic spread of wheat, and inform basic strategies for future exploitation of the unique characteristics of adaptation, performance, stress tolerance, and end use quality exhibited by landrace cultivars.

Consensus mapping is an important method in crop genomic research. In bread wheat, the dense microsatellite consensus map created by somers *et al.* (2004) was a major achievement in defining genetic loci beyond a single biparental map. High-throughput genotyping techniques have since then been used to overcome marker density limitations, but still, to our knowledge, there are currently just five high-density consensus maps publicly available. One of these is for tetraploid wheat, constructed from 13 mapping populations (maccaferri *et al.* 2014). Three other consensus maps for hexaploid wheat were either based on six biparental populations and one multi-parent advanced generation intercross (MAGIC) population (cavanagh *et al.* 2013), on six biparental doubled haploid (DH) populations that included four synthetic bread wheats among the parents (wang *et al.* 2014), or on three biparental DH populations (winfield *et al.* 2016). A further new high-density map was constructed from an eight-parent MAGIC population (gardner *et al.* 2015) and was not derived from biparental maps.

Marker distance is a measure of the number of recombinations observed between two genetic markers on a chromosome (kearsey and pooni 1996). As such, it reflects the physical length of that chromosomal region, as there is more opportunity of recombination in a longer region, but it can also reflect the recombination frequency in an organism, which might differ depending on the chromosomal regions. Within the same species, assuming that no major reductions in physical length of the chromosome have taken place, individuals with a higher recombination rate should have wider marker distances.

Marker order is reported to be highly conserved in cereals at the recombination map level, but this collinearity is often not observed at the level of local genome structure (bennetzen and ma 2003). Within one species, the marker or gene order should be similar between individuals; however, the degree of similarity will depend on the species (moore *et al.* 1995).

Segregation distortion is the deviation of the segregation ratio from the expected Mendelian ratio observed at a locus (xu 2008). The loci concerned are called segregation distortion loci (SDL) and they tend to cluster in segregation distortion regions (SDR) in the genome. It is unknown whether SDR are common in different segregating mapping populations (liu *et al.* 2010). Different factors influencing segregation distortion in plants including type of mapping population, gametophytic competition, abortion of gametes or zygotes, unbalanced meoitic products, and response to environmental conditions (liu *et al.* 2010, 2011). Segregation distortion can also occur as a result of conscious or unconscious selection during the development of mapping populations (li *et al.* 2015). Segregation distortion may be related to the genetic background of the parents and is higher in interspecific populations than in intraspecific populations, *e.g.*, in maize and related species (wang *et al.* 2012). In rice, gamete transmission was found to be influenced by female, male, and zygotic selection, which gave rise to SDL (Reflinur *et al.* 2014). kumar *et al.* (2007) reports on three SDR on chromosome 5B of tetraploid wheat due to competition among male gametes. A study in a wheat MAGIC cross found a number of at least 39 segregation distortion blocks distributed over the genome, with the chromosomes 1B, 6B, 3B, and 4A showing more distorted markers (gardner *et al.* 2015). The importance of SDL for breeding programs has been discussed for at least three decades (zamir and Tadmor 1986).

Chromosomal rearrangements, such as inversions and translocations, are large-scale mutational events and play a role in evolution in intraspecific divergence and speciation (rieseberg and willis 2007). badaeva *et al.* (2007) characterized chromosome diversity in a broad taxonomic and geographic range of wheat using C-banding. About 30% of the accessions screened showed rearrangements, with the nature of translocations either being single translocations (67%), multiple rearrangements (17%), or inversions (16%). Not all described wheat translocations were found, *e.g.*, T1B:2B (previously discovered in bread wheat) (friebe and gill 1994), was not detected; however, the most frequent translocation, T5B:7B (riley *et al.* 1967), was. Bread wheat landraces showed a broader spectrum of translocation types than commercial lines, most at low frequencies. The spatial resolution of neighboring loci is very limited in chromosome banding techniques. More detailed assessments of the chromosomal rearrangements can be made with other techniques like aneuploid analysis or genetic map analysis.

Genetic recombination is a central mechanism in evolutionary processes and is equally central to crop breeding, where the modification of recombination is of interest. Recombinant inbred lines provide information to estimate the crossover number. This allows the detection of segregating QTL influencing recombination (dole and weber 2007). In *Arabidopsis*, a study of 17 F2 populations revealed a lower number of crossovers than found in yeast, mice, and human (salomé *et al.* 2012). This study also suggested that recombination hot spots are accession-specific. For wheat, a better understanding of the recombination landscape would be important to achieve breeding targets. Additionally, to identify useful alleles, it is also of importance to predict how much a crossing program will be helped or hindered by recombinational hot spots or blocks.

The aims of this work are to report on the diversity and plasticity of the wheat genome by analyzing genetic maps from a novel nested association mapping panel (NAM) for wheat, in a similar approach as used in maize (yu *et al.* 2008). The new NAM panel consists of 60 biparental populations, most of them developed from a diverse core set of the A. E. Watkins hexaploid wheat landrace collection. This collection, established in the 1930s, comes from a wide geographic spread, has been shown to have a high genetic diversity, and reveals at least nine ancestral groups of precommercial wheat (wingen *et al.* 2014). It has been extensively screened for the presence of resistance genes (thompson and seymour 2011; bansal *et al.* 2013, 2015; burt *et al.* 2014; li *et al.* 2016) but the identification of new alleles of complex genes has, to date, been limited (qamar *et al.* 2014). The collection is one of the main resources of a UK prebreeding program (moore 2015; winfield *et al.* 2016). All of the biparental populations share the reference parent “Paragon” (Par), a hexaploid UK elite spring wheat cultivar. This line was chosen as it had been a very successful elite line in the UK environment and was already a key parent for existing Wheat Genetic Improvement Programme (http://www.wgin.org.uk) resources including EMS and γ mutant populations. To best achieve the aims: (i) genetic mapping for 60 biparental populations was conducted following set rules; (ii) a consensus map was constructed; (iii) map length and marker distances were compared between maps; (iv) marker orders were compared between maps; (v) loci with segregation distortion were identified; (vi) translocation events were detected and evaluated; and (vii) recombination QTL were calculated.

## Materials and Methods

### Plant material and growth conditions

Biparental segregating populations were developed as a NAM panel from crosses between a reference variety, the spring bread wheat cultivar Par (mainly as pollen recipient), and a further bread wheat variety or cultivar. For 55 populations, the pollen donor was a single-seed descendent (SSD) from a landrace accession from the A. E. Watkins collection (wingen *et al.* 2014); a further five populations were developed with the varieties “Pamyati-Azieva,” “Chinese Spring,” “Garcia,” “SS7010073,” and “Glasgow” either as pollen donor or recipient (see Table 1 for details on populations, and Supplemental Material, Table S1 in File S1 for details on accessions). The initial crosses were followed by either four, five, or six rounds of self-pollination as SSDs, so individuals were recombinant inbred lines (RILs). All plants were grown under standard glasshouse conditions under regular mildew, aphid, and thrips control measures applied following the manufacturers’ recommendations. Two generations were grown per season, during the summer season (Mar–Aug) under natural conditions and during the winter season (Sept–Feb) at 20° with supplementary light (400 W high-pressure sodium lights, $180-250\;\text{μmol}/{\text{m}}^{2}\text{s}$) to 16 hr light.

**Table 1:** List of populations and their characteristics as determined in this study

Population name	Pollen Recipient	Pollen Donor	Population Size	Linked Markers	Cosegr. Markers	Map Length (cM)	Number of LGs	Missing D LGs	Incongruent LGs	Expanded LGs	Segr. D. (%)	More Par Allele	More Other Allele	Translocations	CO Count	CO QTL
ParW007	Par	W007	94	162	25	838	33		1	0	3.14	5	0	1	12.1	1
ParW034*^a^*	Par	W034	94	423	129	1511	31		0	0	6.42	18	6	8	37.6	4
ParW044	Par	W044	94	176	15	1251	34		0	1	11.63	20	0	2	17.0	13
ParW079	Par	W079	94	170	20	1202	30		0	0	1.84	1	2	2	17.1	3
ParW103	Par	W103	93	251	56	1110	29	4	0	0	0.4	0	1	3	18.3	5
ParW110	Par	W110	94	172	15	1328	25	4	0	4	15.15	24	1	3	18.7	4
ParW139	Par	W139	94	178	26	981	26	3, 4	1	0	4.02	6	1	2	14.9	3
ParW141*^a^*	Par	W141	94	482	206	1556	29	4	1	0	7.08	15	9	0	27.0	4
ParW145	Par	W145	94	182	30	1172	34	7	0	0	3.87	2	5	1	16.8	1
ParW199	Par	W199	93	503	219	1388	30		0	0	0.6	1	2	1	23.2	2
ParW209*^a^*	Par	W209	87	463	135	1723	26		1	1	7.32	12	15	1	31.9	6
ParW219	Par	W219	94	191	30	1130	27		0	1	1.63	0	3	0	17.0	5
ParW224	Par	W224	94	187	26	1173	35		0	0	1.68	2	1	0	17.0	1
ParW238	Par	W238	89	273	51	1412	25		0	3	1.83	0	5	1	22.1	1
ParW254	Par	W254	94	174	29	1001	32		0	0	0	0	0	1	14.0	1
ParW273	Par	W273	94	198	21	1288	30		1	1	1.04	2	0	1	18.7	2
ParW281	Par	W281	94	214	15	2052	28	3	4*^d^*	9*^c^*	35.1*^d^*	73*^d^*	0*^d^*	47*^d^*	29.1*^d^*	18*^d^*
ParW292*^a^*	Par	W292	94	522	170	1687	27		0	1	5.91	7	16	0	31.9	2
ParW299	Par	W299	93	291	58	1442	27		1	1	0.35	1	0	3	23.2	4
ParW300	Par	W300	87	198	31	1376	26		0	0	0.51	1	0	2	20.3	2
ParW308	Par	W308	80	281	61	1595	26		0	0	3.62	9	1	4	24.1	3
ParW313	Par	W313	94	184	23	1841	27		5*^d^*	7*^c^*	30.51*^d^*	54*^d^*	0*^d^*	27*^d^*	24.9*^d^*	9*^d^*
ParW324	Par	W324	94	157	18	831	25	3, 4, 7	1	2	2.01	1	2	1	12.6	2
ParW352*^a^*	Par	W352	94	499	202	1419	25		0	2	10.8	20	17	4	27.9	5
ParW360	Par	W360	94	156	22	1125	24		0	1	2.65	3	1	0	15.6	1
ParW387	Par	W387	94	191	30	1214	26		0	0	3.78	1	6	1	17.3	2
ParW396	Par	W396	94	164	26	953	24		0	1	3.25	2	3	0	14.6	3
ParW406	Par	W406	94	182	20	1356	26		0	0	2.82	5	0	0	19.5	2
ParW433	Par	W433	94	182	17	1457	30		1	0	2.27	2	1	0	16.5	5
ParW440	Par	W440	94	175	25	1059	24		0	2	3.55	3	3	0	15.3	2
ParW468*^a^*	Par	W468	95	468	123	1856	27		1	0	9.76	25	7	4	33.2	5
ParW471	Par	W471	92	281	50	1649	26		0	0	0.36	0	1	4	25.0	3
ParW475	Par	W475	81	182	23	1190	24	6	0	0	1.1	1	1	3	17.5	5
ParW483	Par	W483	94	165	21	1074	26	4	0	1	0.62	0	1	0	15.1	1
ParW546	Par	W546	94	216	40	1354	28		0	0	0.46	1	0	3	20.2	1
ParW562	Par	W562	94	200	31	1059	29		0	1	0.51	0	1	0	16.5	2
ParW566	Par	W566	90	183	22	1335	24		0	0	1.11	0	2	2	19.4	2
ParW591	Par	W591	94	187	29	1187	26		0	0	0	0	0	2	17.1	2
ParW624	Par	W624	94	167	14	1086	33		1	5	1.26	2	0	3	15.7	3
ParW627	Par	W627	93	482	225	1370	24		0	0	1.25	6	0	2	22.9	6
ParW629	Par	W629	94	211	33	1187	23		0	0	0	0	0	4	18.2	3
ParW651	Par	W651	94	161	24	1061	26		0	0	0.65	1	0	0	14.8	0
ParW652	Par	W652	94	186	25	1385	32		0	2	0	0	0	0	18.8	1
ParW670	Par	W670	94	185	25	1080	23	3	0	1	1.14	1	1	1	16.3	1
ParW680	Par	W680	94	183	21	1130	28		0	1	2.27	1	3	1	16.0	3
ParW694	Par	W694	94	184	16	1167	25		0	1	4	7	0	2	18.0	0
ParW707	Par	W707	93	234	35	1456	24		0	1	1.72	0	4	4	22.8	2
ParW722	Par	W722	94	157	22	1140	26	3, 4B	0	0	1.32	1	1	0	15.0	5
ParW729*^a^*	Par	W729	82	500	136	1975	27		0	4	4.42	4	10	1	36.0	8
ParW731	Par	W731	93	194	24	1316	25		0	2	0.52	0	1	4	20.2	1
ParW740	Par	W740	94	164	25	940	28		0	0	1.26	1	1	0	14.1	1
ParW777	Par	W777	93	236	34	1692	27		0	1	0.43	0	1	5	25.2	2
ParW784	Par	W784	94	163	17	1142	26		0	1	3.18	1	4	1	16.4	1
ParW811	Par	W811	94	274	53	1340	31		0	0	1.1	0	3	3	22.6	3
ParW827	Par	W827	92	445	198	1275	25		0	1	1.35	2	4	3	19.8	1
CSpPar*^c^*	CSp	Par	283	284	49	1250	21	3, 4	0	0	2.11	1	5	1	19.7	0
GlaPar*^b^*	Gla	Par	163	213	29	1039	30	7	0	0	1.89	1	3	0	16.6	2
PamPar*^b^*	Pam	Par	94	124	9	984	22	1, 4, 6	1	2	1.72	0	2	1	14.4	2
ParGar*^b^*	Par	Gar	351	175	8	1558	28	3, 4	1	0	7.51	3	10	3	21.6	4
ParSyn*^b^*	Par	Syn	251	374	73	1549	22		1	0	6.11	9	13	6	25.1	4
Mean			104	246	53	1305	27.1		0.22	0.78	2.9	3.98	3.1	1.81	19.9	2.8
SD			46	114	58	271	3		0.42	1.12	3.17	6.27	4.18	1.75	5.7	2.2
Minimum			80	124	8	831	21		0	0	0	0	0	0	12.1	0
Maximum			351	522	225	2052	35		1	5	15.15	25	17	8	37.6	13

Populations were developed as single seed descent (SSD) up to at least generation F4. Populations with higher generation numbers (F5 or F6) are indicated. In general, SNP markers were used for genotyping. For the first seven populations developed some SSR markers were used. “Missing D LGs” refers to LGs (linkage groups) not present in the map, all but one named exception (4B) from the D genome. “Incongruent LGs” refers to the number of LGs found to be incongruent with the consensus map. “Expanded LGs” refers to the number of LGs that exhibited a much larger mean marker distance compared to other LGs for that chromosome. “Segr. D” refers to the segregation distortion and is given as a percentage for that population. “Excess Par/other SDL” refers to the number of loci that show more alleles from Par, respectively, than the other parent. “Translocations” refers to the number of translocations detected in that population. “Crossover estimate” refers to the mean number of crossover estimated for that population. “Crossover QTL” refers to the number of detected QTL for the trait crossover estimate. Several statistics for two populations (ParW281 and ParW313) were taken out of the general analysis as the populations showed an unexpected allele distribution. Cosegr., cosegregating; LGs, linkage groups; Segr., segregating; CO, crossover; Par, “Paragon”; W 〈number〉, Watkins accession 1190〈number〉; CSp, “Chinese Spring”; Gla, “Glasgow”; Pam, “Pamyati-Azieva”; Gar, “Garcia”; Syn, “SS7010073.”

a Initial populations developed.

b F5.

c F6.

d Result not included in overall analysis.

### Genotyping

DNA extractions and Kompetitive Allele Specific PCR (KASP)MT SNP genotyping were essentially carried out as in knight *et al.* (2015). Primer information for the markers, developed by the University of Bristol, and genotyping protocols can be found at CerealsDB (http://www.cerealsdb.uk.net/cerealgenomics/CerealsDB/). Markers were selected from a core set of codominant, genome-wide, reliable high-performance markers from CerealsDB, chosen for high levels of polymorphism over multiple populations to produce common sets. They were also selected to maximize the coverage of each of the 21 chromosomes with a low marker number. However, marker selection suffered from information on SNPs and their chromosome locations becoming only gradually available over the time course of the project. Additionally, information on polymorphisms was usually restricted to a subset of parental lines. Some addition of markers to populations that were genotyped early in the project was conducted to increase the marker overlap between these and later populations, but was limited by time and monetary constraints.

An initial set of seven biparental populations were also genotyped with 31 SSR markers, which were selected from the markers used in wingen *et al.* (2014) (see Table 1). These seven and three additional initial maps were constructed from an average number of 488 markers (SD 30) whereas the following maps were constructed on average from only 195 SNP markers (SD 39), which had been selected for best genotyping performance (see Table 1). Overall, > 2400 single SNP markers were employed leading to > 1,613,786 genotype points on the 60 populations. Unfortunately, for some of the genotyping of the initial set of seven biparental populations, genotype scoring recorded the heterozygote scores as missing. Hence, markers not showing a single heterozygote score in one of these seven populations were excluded from the test of segregation distortion and detection of translocations (between 169 and 279 markers per population), which left 206–254 markers on these populations. On average, a marker was mapped on 5.6 of the 60 populations; however, this number was higher for markers used later in the project. A marker from the subset not employed to genotype the first seven populations was on average mapped on 7.8 populations.

Given that a very large population development program was conducted, it was ensured that populations originated from the named parents and that no mix-up of populations had happened by genotyping the F1 plants with characteristic markers. Genotyping results of F1 and F4 plants were compared. Only populations with genotype consistency were used.

The mean allele distribution over all loci of each population was close to that expected with two exceptions. In those populations, the homozygous Par allele ratios were larger (≥0.54) than the expected ratio of 0.4375, and the ratios for the second parent were lower (≤0.37, $\chi^{2}:$ P=0.08 and P=0.12 for ParW281 and ParW313, respectively). No explanation for these unexpected ratios has been found. As these populations were frequently found in the outlier groups of the applied statistics they were excluded from most of the analyses.

### Genetic map and consensus map construction

Genetic maps were constructed for 60 populations using the software program MapDisto v. 1.7.7 (lorieux 2012). Genotype data were presorted following the marker order seen in existing maps, before importing into MapDisto. To construct the linkage groups (LGs), a LOD score of 3.0, a maximum recombination frequency of 0.3, and removal of loci with 10% missing data were set as constraints. Map distances were calculated using the Kosambi mapping function (kosambi 1943). Loci were ordered using the Seriation II method, with the minimum Sum of Adjacent Recombination Fractions (SARF) criteria for ordering and progressive rippling. LGs were further split if there was a distance ≥ 35 cM between two adjacent markers. For consensus mapping, map files were split into individual LGs for each chromosome. Maps were used without any weighting factors. Common marker order between LGs of different maps for each chromosome was compared by rank correlation. Those LGs that had a mean negative correlation when compared to all other groups had their order reversed. LGs that had no markers in common with any other populations were discarded.

A consensus map was created for each chromosome using LPmerge (endelman and plomion 2014). Input parameters specified maximum map intervals (*K*) of 1–3, with each LG being equally weighted. Different consensus maps were produced for each value of *K*, and for each chromosome the consensus map with the lowest SD and mean root mean-squared error (RMSE) was chosen as optimal. This was in most cases *K* = 3, but it was *K* = 2 for chromosomes 1B, 6B, and 6D.

The final consensus map, the landrace consensus (LRC) map, was compared to two publicly available maps. The first one, the “Avalon *x* Cadenza” (AvaCad) map, belongs to a population previously developed at the JIC (griffiths *et al.* 2009) that is widely used by the UK research community. The map was based on genotyping on the Illumina Infinium iSelect 90,000 SNP array by the University of Bristol, which consisted of 3970 polymorphic markers. Data and maps are available from CerealsDB. The second map is the consensus map constructed from six biparental populations described in wang *et al.* (2014), here called Wang map, which consists of 40,267 markers.

The centromeric regions were estimated by annotating LRC markers with chromosome arm information made available by the International Wheat Genome Sequencing Consortium (IWGSC, www.wheatgenome.org). The region of transition between short arm and long arm assignments was classified as centromeric.

### General statistics

Statistical analysis was conducted using the R software suite (*vs.* 3.2.3) (r core team 2015) if not stated otherwise. Box plots were plotted using the cars package. Correlation was conducted as Pearson’s product-moment correlation $\rho_{p}$ for numeric values or Spearman’s rank correlation $\rho_{s}$ where the order or markers was compared (both using functions cor and cor.test). The order of markers in each LG was compared to the corresponding LG in the other biparental maps and in the LRC map to find maps with different marker order. Linear model analysis was conducted with function lm and mixed linear models with lmer from the lme4 package. Adjustment of *P*-values for multiple testing was done following the Benjamini–Hochberg method using function p.adjust. Heat maps were constructed using the heatmap.2 function in the gplots package.

### Map lengths, marker distances, and marker order

**Figure 1:**
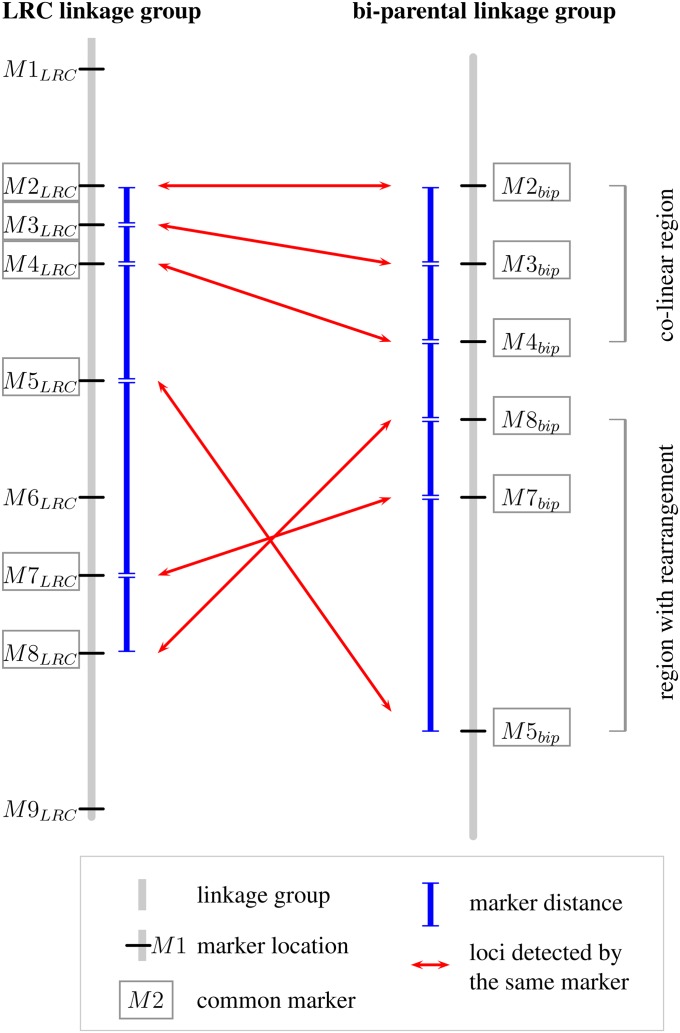
Comparison of marker distances and marker order between the LRC map and a biparental map. To calculate the mean MDR and marker order correlations, the common markers between each LG are identified first. Only one LG shown. LGs are depicted as gray vertical bars, loci are given as short black horizontal bars, and the marker names (M1–M9) are given next to the loci. A frame around the marker name signifies a shared or common marker between the two maps. For MDR, the MDs, shown as blue bars next to the LGs, are calculated for both LGs separately. In this example, if $m_{i}$ is the centimorgan position of marker Mi, the two means are $MD_{LRC}=\left(m_{8}-m_{2}\right)/5$ and $MD_{bip}=\left(m_{5}-m_{2}\right)/5.$ MDR is calculated from these means as: $MDR=\left(MD_{bip}\right)/\left(MD_{LRC}\right).$ To assess differences in marker order between the two maps, the rank difference between the common markers is used to calculate the Spearman’s rank correlation coefficient $\rho_{s}.$ In this example, as M5 and M8 swapped places, rank differences are zero for these two markers only. The regions from M2 to M4 seem to be colinear, while the regions from M5 to M8 seem to be rearranged between the two maps. bip, biparental; LG, linkage group; LRC, landrace consensus; M, marker; MD, mean of all distances of adjacent common markers; MDR, marker distance ratio.

The biparental linkage maps were compared with one another and with the LRC map regarding different characteristics: the total and chromosomal map lengths, the marker distances, and the correlation of marker orders (see Figure 1). A marker distance was calculated as mean centimorgan distance between adjacent markers in each biparental LG and then compared to the corresponding marker distance in the LRC map as mean marker order ratio (MOR), using a ratio of the biparental or any other map mean to the LRC map mean.

Cases where LGs were of length 0 cM, usually only two or three markers long, were not included in the analysis as they appeared to artificially extend the MOR range to zero, which seemed to be against the general trend. Exceptional cases of much longer or shorter MORs are defined similar to box-plot outliers. MOR exceptions are 1.5 times the interquartile range (IQR) away from the bordering quartile values, either below quartile one (Q1) or above quartile three (Q3), with IQR=Q3−Q1.

The exceptional cases above Q3 are assumed to have expanded marker distances. Also, the overall LG lengths of the LRC map were compared to the lengths of respective LG in the AvaCad and Wang maps. Moreover, the marker order of biparental LGs were compared to the LRC map using the Spearman’s rank correlation coefficient $\rho_{s}.$ Cases of low correlation, $-0.6\leq \rho_{s}\leq 0.6,$ were referred to as incongruent maps. For values between $0.6\leq \rho_{s}\leq 0.7$ the expression near incongruent will be used. Alignments of LGs to illustrate the differences between LG from different maps were drawn using Strudel software (*vs.* 1.15.08.25, https://ics.hutton.ac.uk/strudel/). For an alignment of the LRC map and the Chinese spring (CSp) genome, the marker sequences were blasted against the IWGSC whole genome assembly (WGA) v0.4 (NRGene DeNovoMAGIC) at Unité de Recherche Génomique Info (https://urgi.versailles.inra.fr/blast_iwgsc/) and positions with the highest or a very high BLAST score on the most likely chromosome were selected manually from the BLAST output. Alignments were represented as Strudel plots.

### Further genetic statistics

Segregation distortion, chromosomal translocations, crossover count, and crossover QTL were detected using the package qtl (*vs.* 1.35, broman *et al.* 2003) for R software. The qtl package took the cross type and generation number of the populations (Table 1) into account by using in the read.cross function options “BC.gen = 0” for all populations, and “F.gen = 4” or “F.gen = 5” depending on the respective generation. As an exception, CSpPar was treated as a complete RIL population (“crosstype = riself”). Segregation distortions were calculated as separate nonindependent $\chi^{2}$ tests at each locus on the imputed genotype probabilities generated by function fill.geno and method maxmarginal to correct for obvious mis-scoring. *P*-values were adjusted for multiple testing. Markers with no heterozygote states in F4 and F5 populations were excluded from the segregation distortion analysis, as a likely explanation is an erroneous categorization of the heterozygous markers with one of the homozygous groups during the KASP marker scoring. The countXO function was used to estimate the number of crossovers per line. These values were used as traits for QTL analyses. QTL analyses were performed in two steps: putative QTL were identified in an initial single QTL scan and subsequently tested in a final multiple QTL model using a significant threshold calculated from the data distribution. Major translocations were detected in the genetic maps where markers from different chromosomes were found in one LG, as most SNP markers carried a chromosomal assignment. Additionally, putative translocations were identified from a linkage test using the pairwise estimated recombination fractions (estRF) between markers, calculated by function est.rf. Linked markers usually display a high LOD score. Putative translocations were detected in pairwise estRF comparisons as markers being strongly linked to markers outside their LG indicated by a LOD score higher than 10. We felt that this was a plausible approach, as known translocations, *e.g.*, the T5B:7B reciprocal chromosome translocation, present in cultivar “Avalon” inherited from “Cappelle-Desprez” (riley *et al.* 1967), gives rise to such a translocation signature in the estRF LOD scores in the cross AvaCad (result not shown). The threshold of LOD 7.0 was determined by comparing the number of expected translocations for different LOD thresholds with those translocations observed in genomic *in situ* hybridization (GISH) experiments.

### GISH

Multicolor GISH was performed on mitotic chromosomes of nine selected Watkins accessions following the protocol by zhang *et al.* (2013) and kato *et al.* (2004). The A genome was labeled green, the B genome was labeled purple, and the D genome was labeled red. Between two and five spreads were analyzed for each of the accessions. A green/purple (A:B) translocation was present in all chromosome spreads analyzed. This is an ancient wheat T4AL:5AL:7BS translocation that can be found in both *T. durum* and *T. aestivum* (devos *et al.* 1995). All other translocations observed were taken as characteristic for the accessions analyzed.

### Data availability

Biparental population maps and genotypes are available from http://wisplandracepillar.jic.ac.uk. Biparental populations are available upon request. File S1 contains all supporting tables and figures. File S2 contains the LRC map.

## Results

### Populations, genotyping, and genetic map construction

This study reports on the development and characterization of a novel bread wheat NAM panel consisting of 60 biparental populations (55 at generation F4, 4 at F5, and 1 at F6). All populations share Par as reference parent. The second parent for the F4 populations was a landrace accession taken from the 119 accessions-strong core collection of the bread wheat Watkins collection, selected to capture a maximum of the genotypic diversity (wingen *et al.* 2014). The 55 accessions cover all of the nine ancestral groups described to be a representative subsample and come from 21 of the original 32 countries of the whole collection (Table S1 in File S1). The average size of the populations is 92.7 individuals (range 80–95 individuals) for the landrace populations and PamPar (details on crosses and maps in Table 1). The other SSD populations were larger, being composed of between 163 and 374 individuals (mean 262.0). Genetic mapping was conducted on mainly SNP genotypic information following the same rules for all maps. As expected, the number of markers used for genotyping was strongly correlated with the number of linked markers ($\rho_{p}=0.99$). This also reflected the strategy to use previously validated markers that resulted in a reasonable spread along the chromosomes.

### Genome coverage

The average marker number per chromosome was similar between the A and B genome (mean 14.8, range 7.9–31.3 and mean 13.9, range 7.7–31.5, respectively). Coverage was satisfactory for these chromosomes. As expected, the number on the D genome was lower (mean 6.2, range 1.7–13.7), as due to a lower diversity of the D genome the number of genetic markers available was limited. In 25% of the populations, the constructed linkage maps lacked one chromosome or more, in comparison with the full wheat genome of 21 chromosomes (Figure S1 in File S1 and Table 1).

Mainly, chromosomes of the D genome appeared to be missing, apart from one case where chromosome 4B was absent. It is unlikely that these chromosomes were truly absent, and they may only have appeared to be missing due to random markers not being polymorphic in some populations. Given the low marker density of the maps, a few markers can define a chromosome and, with the exception of the ParW141 map, all maps with missing chromosomes had a low marker number. The actual presence of two seemingly missing chromosomes (ParW722 4B and ParW141 4D) was confirmed in these cases by targeted genotyping (data not shown). The particularly low number of D markers would explain the absence of predominately D chromosomes. The low coverage of the D genomes may have had other unintended consequences for the statistics employed. Any anomalies found in the characteristics for the D genomes of the NAM panel were, thus, considered to be caused by low marker coverage if there was no other more likely explanation.

### Consensus mapping

A consensus map for the NAM panel, here called the LRC map as 55 landrace accessions were included as parents, was constructed. The LRC map contains 2406 markers, all SNP markers apart from 33 SSRs, on 2498 loci. Similar numbers of loci fell on the A and B genomes: 951 loci and 1122 loci, respectively. Only 425 loci fell on the D homeologous genome (Figure S2 and Table S2 in File S1). Counting the number of markers genotyped per single map, there were a total of 6213 markers on the A genomes, 5855 on the B genomes, and 2575 on the D genomes. All larger LGs could be combined into the LRC map; however, some conflicts of marker order were reported, which were dealt with following the linear programming strategy. This means that the LRC map is a generalization of the order of the individual maps and may, in regions where ordering conflicts were reported, represent the majority of the maps only. On average, 5.6% of the marker order comparisons resulted in an unresolvable ordering constraint.

### Map comparisons

The characteristics of the 60 biparental maps and the LRC map were assessed by comparing the following properties: map or LG lengths, marker distances, and marker order. Furthermore, a comparison with two recent high-density maps, the AvaCad map and the Wang map, was conducted.

#### Map lengths:

A range of 831–2050 cM for the map lengths of the NAM populations was found (Table 1). There was a positive and statistically significant correlation between the number of linked markers and the map length ($\rho_{p}=0.58$). As stated above, map length variation was one characteristic of interest. It was noted that, with $R^{2}=0.34,$ the variation explained was only about one-third of the total variation. Thus, we felt confident that variation in map length was determined by more than the number of markers used for genotyping. However, further factors that influence the differences in map length cannot be derived from this analysis. Some populations of the same size, genotyped with a similar marker number, and having a similar number of LGs, *e.g.*, ParW406 and ParW475, show differences in map length, in the example case by 12% (1356 cM *vs.* 1190 cM). It is likely that the major factor in the observed differences is down to random genotyping effects, *e.g.*, where chromosomes remained undetected the maps were shorter (Table 1). It is possible that different recombination rates contribute to map length differences but it is difficult to find conclusive evidence for this in the overall comparison of map lengths. There was no significant correlation between population size and map length. The LRC map is 1862 cM long. This is longer than the mean map length of the biparental set (1310 cM), but shorter than the longest biparental map (2050 cM) included in the consensus mapping (Table 1).

The LRC LGs were compared to those of two high-density maps with respect to map length, with the aim of getting some reference points for the LRC map (Table S2 in File S1). In general, the LRC LGs were much shorter than those of the other maps, reaching on average only 51% of the Wang LGs and 57% of AvaCad LGs. Map length seems to be strongly determined by the number of markers used, while marker saturation has not been reached. This explanation is supported by the LRC D LGs having a low marker coverage, with several cases of whole chromosome arms not being detected (Figure S2 in File S1, LGs 3D, 4D, 5D, 6D, and 7D) and being particularly short. We conclude that the map length values of LRC and NAM maps do not provide unique characteristics for the used populations and their parents as the marker density of the maps was below a level that would ensure that the calculated map lengths are closely correlated to the true map lengths.

#### Marker distances:

**Figure 2:**
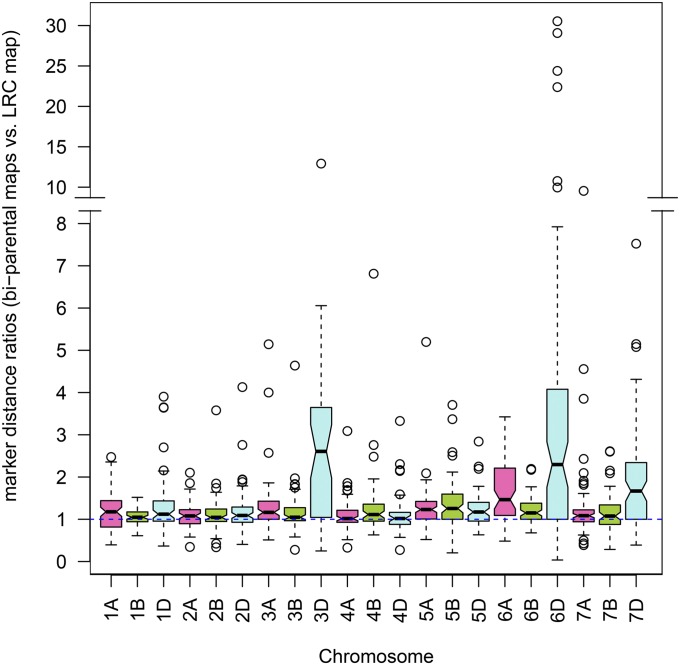
Marker distance ratios per chromosome. Ratios of marker distances are the mean distances between adjacent markers for 60 biparental populations divided by the landrace consensus (LRC map) marker distances.

The distances between common markers were compared between maps to find expansion or reduction of LG regions. Differences would be expected if recombination rates were higher or lower than average in a map. For this, the mean marker distance ratio (MDR) (for method see Figure 1) between the biparental maps and the LRC map was calculated. The comparison revealed different levels of MDR, when applied at the LG level (Figure 2), meaning that marker distance is partly a function of the LG.

The average MDR over the A and B genome LGs is 1.22. This means that the marker distances in the biparental maps are, in general, larger than those in the LRC map, which is consistent with the reported marker compression by the consensus mapping procedure (endelman and plomion 2014). The 6A LGs show the highest MDR (1.65, SD = 0.69) and also the widest IQR (extension of the boxes in Figure 2). The 5B LGs show the next highest MDR (1.33, SD = 0.54), but the value is not very far from the overall mean and the IQR is also much narrower than that for the 6A LGs.

Apart from these general trends, 74 cases were detected where LGs in individual populations showed much higher MDRs (outliers in Figure 2). These are putative cases of a higher recombination rate, possibly restricted to the chromosome level rather than the whole genome level as none of the populations has statistically significantly more outliers. A higher MDR can also be the result of longer physical chromosome in the biparental population; sequencing of regions with unusual MDRs would help to distinguish between the two options. To restrict a detailed analysis to robust cases, only those LGs that exhibited expanded MDRs and consisted of a minimum of six markers were considered, reducing the number of outlier cases to 20 (Table S3 in File S1). Five of the expanded MDR cases concerned the 3B LGs, three the 1D LG, and the rest either two LGs (2B, 7B) or just one LG (2A, 2D, 5A, 5B, 5D, 6B, 6D, and 7A). Interestingly, for chromosome 6A, where the highest average MDR was found, none of the 6A LG fell into the category of expanded MDR. This is partly explained by the 6A MDRs having a higher dispersion than the other LGs. The higher dispersion results in a higher IQR and, thus, leads to a higher threshold in the detection of expanded MDR. However, no particularly extreme outliers were observed for the 6A LGs, as there were for several of the other LGs.

Comparing the MDR of the LRC map to the two high-density maps in general shows that marker distances are smaller in the LRC map (Table S2 in File S1). Due to the differences in marker number and either the different nature of the map (AvaCad map is biparental) or the different way of construction (MergeMap was used for the Wang consensus map construction) these comparisons are not very informative. However, it seems to be of interest that the 6A LG of the Wang map shows a large MDR of 3.2 (with an average MDR of 1.6 for the A and B genome), which points to a possible common mechanism for the marker distance diversity found in the biparental maps for this chromosome.

### Marker order comparison

The marker order of each LG of the 60 populations was compared to the respective chromosome of the LRC map. In total, there were 1611 LGs to compare; the results are thus summarized and a few extreme cases discussed in more detail.

In general, most LGs show good correlations between biparental populations and the LRC map, and only 61 LGs (5.7%) were incongruent with the LRC map (Table 1). The number of incongruent LGs per chromosome varies between zero and seven, with 6B having the highest number of incongruent cases (Table S4 in File S1). However, no specific pattern seems to be present that would explain differences between chromosomes.

**Figure 3:**
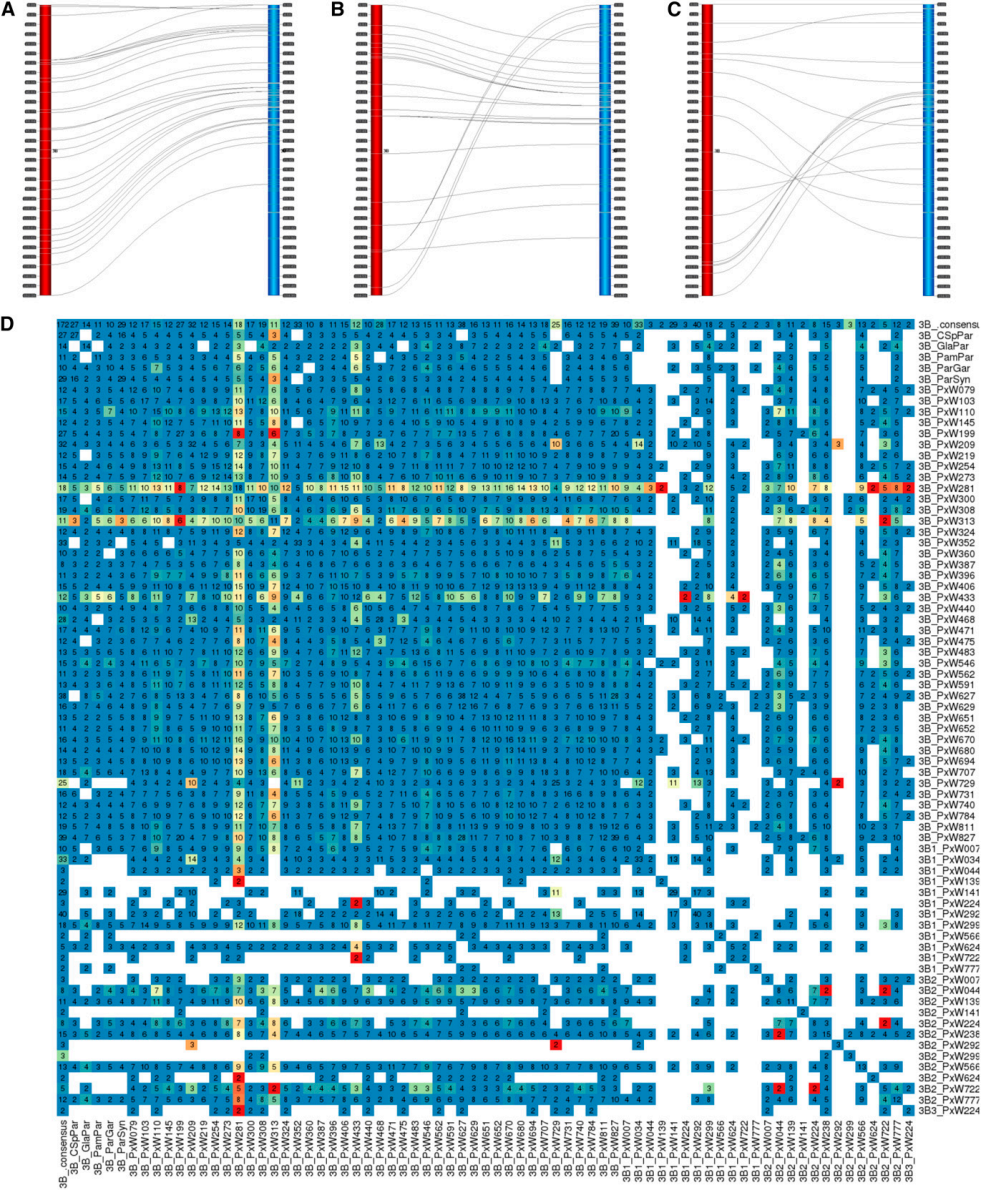
Alignment of 3B LGs of the biparental NAM populations and the LRC map. (A–C) Alignment between biparental LG (depicted as red vertical bar) *vs.* the LRC LG (blue) as Strudel plots. Markers shared between LGs are connected. Positions of markers are given in gray boxes at the sides of the bars. (A) ParW209 *vs.* LRC: the alignment seems near perfect, distances between markers show variations. (B) ParW729 *vs.* LRC: a putative chromosomal translocation of the bottom part of the ParW729 3B LG is suggested by the alignment of that part to the top short arm of the LRC LG. (C) ParW281 *vs.* LRC: the alignment seems to suggest several rearrangements, including an inversion in the lower middle part and a translocation of the bottom part of the ParW281 3B LG, which is found in the upper middle region of the LRC LG. (D) Heatmap representing the pair-wise comparisons of the marker order between all 3B LGs of the NAM populations and the LRC map. Names of the populations are given to the right and below. Heat map colors reflect the Spearman’s rank correlation coefficient $\rho_{s}$ in a color gradient from red (strong negative) via yellow (close to zero) to blue (strong positive). The numbers in the squares refer to markers in common between the LGs compared. The majority of squares show a blue color for congruence between LGs, *e.g.*, for most comparisons with 3B LG of ParW209 [see also (A)]. The 3B LG of ParW729 shows incongruence to some other LGs including the LRC LG [see also (B)]. The 3B LG of the three populations ParW281 [see also (C)], ParW313, and ParW433 appear very incongruent to others. Gaps in the matrix are due to not enough common markers. LG, linkage group; LRC, landrace consensus; NAM, nested association mapping.

As an example of a particularly congruent chromosome, LG 7A shows a nearly uniform marker order in all populations and only one incongruent case is discovered (Figure S4 in File S1). However, for the majority of LGs, a few incongruent cases are present, *e.g.*, for LG 3B three populations showed distinct marker order changes (Figure 3D). In contrast, LG 6B appears to have the most variation in marker order, with seven obvious cases of incongruent LGs (Figure S5 in File S1). Two further incongruent 6B cases have fewer than seven markers in common with the LRC 6B LG.

We asked if the incongruent regions might all be of the same nature and belong to a cluster of LGs with alternative marker order. This seems to be the case for chromosome 5A, where a common inversion was found in at least three LGs. In other cases, *e.g.*, chromosome 2B, the incongruent LGs seemed to represent a number of different orders. In some cases not enough common markers are present to decide if LGs incongruent to LRC are aligned with each other or not.

It was interesting to see that some populations showed more changes in marker order than others (Table 1). Nearly half of the populations (29) had at least one LG incongruent with the LRC map. In most of these populations, only one to two LGs showed incongruence, but six populations had more.

The marker order of the LRC map was also compared to the AvaCad map and the Wang map (Table S2 in File S1). In general, LRC LGs showed good agreement in marker order with the other two maps. Aligning the LRC map with the CSp wheat genome assembly confirms the general good agreement of marker orders with some local rearrangements (Figure S3 in File S1). It also demonstrates the lower recombination rate around the centromeres, as there are few markers from the genetic LRC map lining up with those regions on the physical wheat genome. The genome regions around the centromeres are particularly sparse in this study due to the genotyping strategy, which followed a low marker density with a preference for markers that are spaced widely along the chromosome.

### Segregation distortion

**Figure 4:**
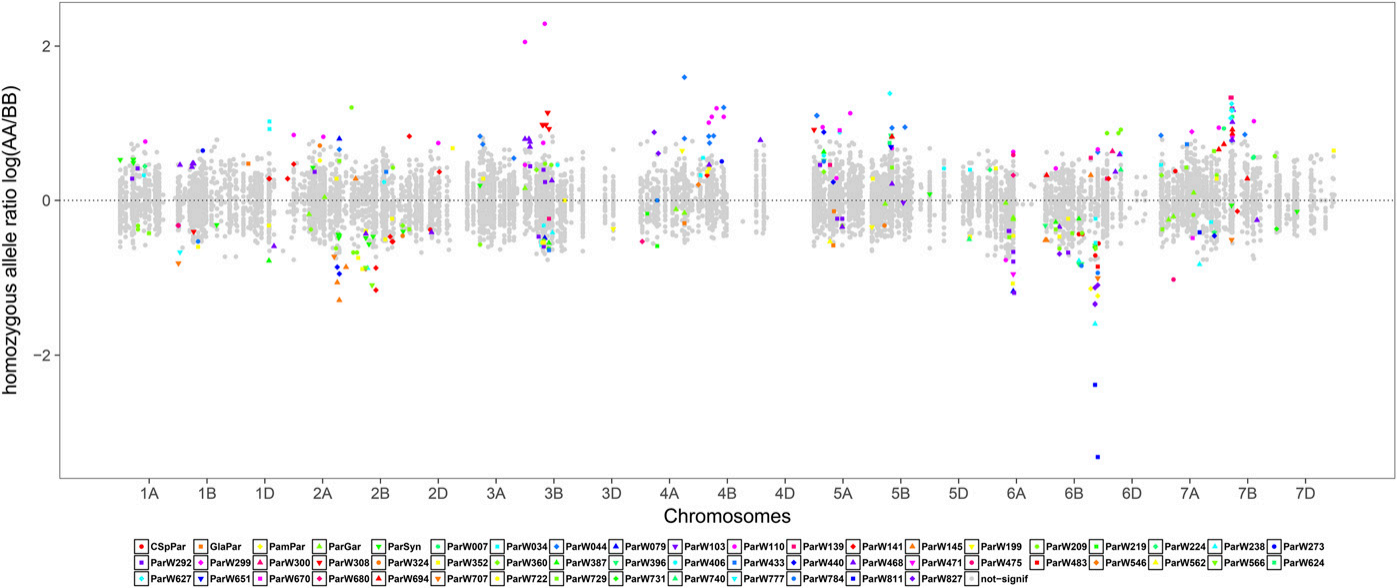
Allele ratios per locus for 54 populations plotted along the genome, highlighting those ratios that show significant segregation distortion. The log ratios of the number of homozygous parental alleles [log(AA/BB), with AA the number of homozygous Par (Paragon) alleles and BB the number of homozygous alleles from the second parent] are plotted along the genome axis according to the marker position on the landrace consensus (LRC) map for each chromosome. Fifty-four populations with segregation distortion were included in the figure. The filled symbols along the map axis signify the allele ratios, either above the axis for an excess of Par or underneath the axis for an excess of the second parent. Gray dots signify ratios that did not show segregation distortion, colored symbols signify statistically significant distorted allele ratios. Neighboring chromosomes are shown in different shades of gray for clarity. Each color–symbol combination stands for a different population as given in the key below the figure.

Segregation distortion of single markers, meaning a statistically significant deviation of the expected distribution of alleles, was found in 413 loci or 3.1% of the loci analyzed (P<0.05, adjusted for multiple testing). The majority of populations (54) carried markers with segregation distortion, however the level of distortion varied widely from an average of 0.35–15.5% of the loci in this subset (Table 1), with 56.2% of the loci having a higher proportion of the Par allele (Figure 4).

Higher numbers of SDL were generally found in the first seven produced populations that had been genotyped with a higher marker number, of which some were not codominant. Populations not in that set, but with high levels of segregation distortion, were ParW110 (15.15%) and ParW044 (11.6%). These populations can be seen as outliers, showing a considerably higher distortion than the majority of populations with SDL (mean 2.9%).

The amount of segregation distortion detected increased with map size but not with population size; however, a linear model taking this explanatory effect into account, explains only 12% of the observed variation. The segregation distortion is not evenly spread over the genome. LGs from the A and B genome show on average between 1.4 and 6.9% of their markers distorted, with LG 7B (7.0%), 6B (6.3%), and 4B (5.5%) having the highest number of distorted markers

Differences are also observed at the single marker level, with hot spots for segregation distortion found for single markers, *e.g.*, loci BS00110651 (6B) is distorted in seven populations and BS00084005 (5A) in six. If the distribution of SDL is plotted along the genome, a number of putative hot spots can be detected, some leaning toward Par, some toward the other parent (Figure 4). The chromosomes have a varying number of hot spots, *e.g.*, chromosome 3A has no hot spots, whereas 3B showed segregation distortion in at least two markers in three or more populations (Figure S8 and Table S5 in File S1).

### Translocation detection from genotype data

The presence of a translocation was assumed if the chromosome assignments of a SNP marker listed on CerealsDB disagreed with the majority of chromosome assignments of the other markers in a LG. Such differences in assignments were observed in general only in a minority of LGs. There were 92 such cases, found in 36 of the 60 maps (see Table S7 in File S1). Two of these 92 putative translocations were found to be reciprocal, thus there were 90 different translocation events, which can be categorized into 38 different types according to the chromosomes involved. Many populations (13 or 36.1% of those assumed to have translocations) had only a single putative translocation, while the rest had between two and eight translocations. Additional chromosomal translocations were predicted by analyzing the recombination fraction between markers. LOD scores above the threshold of seven, measuring the linkage between markers in different LGs, were interpreted as an indication of a translocation. As it was initially not obvious which LOD score threshold would distinguish between a true translocation and a spurious signal, several thresholds were tested. Scores below LOD 7.0 predicted many translocations that could not be confirmed in GISH experiments (see below). Therefore, LOD 7.0 seemed to be the best threshold for this exploratory translocation analysis. With this threshold, 141 translocations were predicted, that would be a translocation in 0.75% of all LGs analyzed. Two populations (ParW281 and ParW313) seemed to have many translocations (27 or 47 cases, respectively). These values seem to be extremely high. Furthermore, both accessions are extreme outliers for other statistics, and the number of translocations found with the initial method of translocation discovery are very low. Thus, we excluded these populations from the analysis, which reduced the number of predicted translocations to 17. The overlap to the cases of putatively mapped translocations was low (two cases). Putting together the putative cases from mapping and from the estRF analysis, we predicted 105 translocation events when analyzing 58 populations (Table 1). Translocation numbers per population ranged between one and eight (mean 4.1, SD 2.4) for the concerned 42 populations with translocations. Of different translocation classes (regarding chromosomal involvement), 29 occurred only once, 37 classes occurred in either 2, 3, 4, or 5 accessions, and 4 more common translocation classes occurred in either 6 (T1B:1D), 7 (T2A:2B), 8 (T3B:7B), or 13 (T5B:7B) accessions (Table S7 in File S1). We assumed that the distribution of the more frequent translocations could show a historic signature, if the translocation originated once and was then passed on. Thus, we tested the distribution of lines with and without the translocation according to their membership with regard to the nine ancestral groups identified by wingen *et al.* (2014). This revealed that the distributions of the accessions carrying T3B:7B or T5B:7B are significantly different statistically from the distribution of accessions without the respective translocation ($\chi^{2}:$ P≤0.05). Thus, it is likely that each translocation originated from one founder event. The number of accessions from groups 1.2 (China–India) and 1.3 (Central/Eastern Asia) with the respective translocation was higher than expected in both cases, which makes it likely that the origin of both translocations was in those regions. For T5B:7B, the number of accessions from group 2.2. (Northwest Europe) was also slightly increased (4 *vs.* 2.5 expected). Given the geographic distance to the other two ancestral groups and a lower degree of enrichment, it seems more likely that T5B:7B did not originate in Northwest Europe but spread there and then became more established in the gene pool. For the other two frequent translocations, T1B:1D and T2A:2B, no difference in distribution could be found. This may mean that they have originated more than once.

### Translocations: GISH experiments

**Figure 5:**
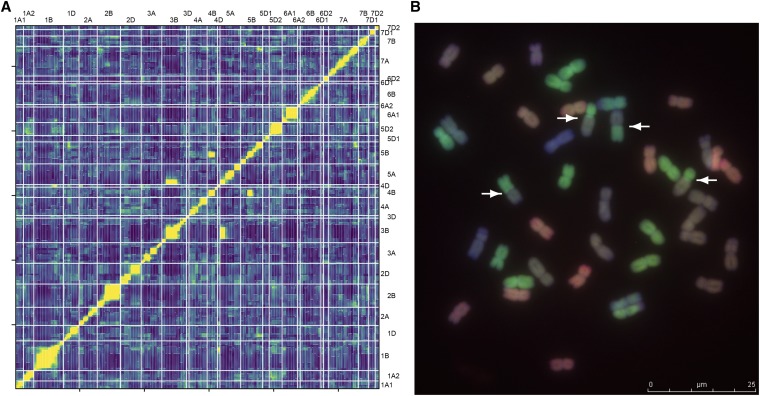
Detection of chromosomal translocations. (A) Estimated pairwise recombination fractions (estRF) plot for W308. EstRFs between markers are plotted as a heat map, all markers against all markers. Markers are in mapping order on both axes. Areas of low estRF, signifying linkage, are represented by yellow squares; estRF values near 0.5, signifying no linkage, are represented by blue squares. Green squares identify values between those two extremes. The yellow diagonal from 1A:1A to 7D2:7D2, demonstrates the low estRF values within linkage groups (LGs). Yellow areas outside LGs, as seen for 3B:5A and 4B:5B, are hypothesized to identify translocations between chromosomes. (B) Genomic *in situ* hybridization (GISH) performed with accession W308, showing 42 chromosomes. Chromosomes are colored according to genome: A, green; B, purple; and D, red. Translocations can be identified between genomes only. A reciprocal A:B translocation, involving nearly half of each chromosome, is highlighted by white arrows pointing at translocation break points.

To confirm the validity of estRF analysis to predict translocations, GISH was performed on 10 Watkins accessions, some of which were showing high LOD scores between markers from different chromosomes and others with lower levels of linkage support from the estRF analysis (Figure 5 and Table S6 in File S1). The GISH performed allowed the detection of translocations between different homeologous groups only, as the different genomes were labeled in different colors. The ancient T4A:5A:7B translocation present in bread wheat was detected in all lines. A further six translocations were detected in the 10 analyzed accessions. All detected translocations could be explained by predictions for translocations made by either genetic mapping or the estRF analysis. One line without a predicted translocation was also found without translocation in the GISH analysis. Two further lines, which did not show a translocation in the GISH analysis, had an estRF LOD value under 7.0. From this, we deduced that only a higher LOD value would predict cytologically visible translocations. Two further cases were predicted to have translocations that we could not detect in GISH. One of those cases was predicted from a single 5B marker in the middle of a 5A chromosome. It is very likely that the GISH procedure was not sensitive enough to find a short introgressed chromosome part. The final case of a predicted T1B:1D translocation could not be explained. However, another 1D translocation (T1D:6A) was predicted in the same accession and detected by GISH (A into D). A further investigation of this case could reveal that the genetic mapping in this accession is impaired at the 1D locus, due to the presence of the T1D:6A translocation, and that the predicted T1B:1D translocation is an artifact of that. The production of a high-density map would possibly be able to support this explanation (See also Figure 5).

### Recombination QTL

From the genetic maps, ∼126,300 crossover events were estimated over all populations, with a mean number of 19.9 crossovers per individual (SD = 5.7, range 12.1–37.6). The mean number of crossovers varied between different populations, and linear model analysis was employed to identify possible covariates that would influence this. Marker number, map size, and population size were all found to have a significant effect on the detected crossover number, and the linear model including these three covariates explained 98% of the variation observed. Thus, it can be assumed that the nonreference parents did not strongly influence the recombination rate. This could result from the fact that either the frequency of recombination events was very similar for all parents, or more likely that an effect coming from the common parent was so strong that any other effects were hidden in this general analysis.

In spite of the absence of an obvious parental effect, in 51 of the 58 populations, 119 significant QTL for the crossover phenotype (LOD ≥ 2.0) were detected (Table S8 in File S1). For 66 QTL, the increasing effect came from Par. For 50 QTL, the increasing effect came from the nonreference parents. The observed additive effects coming from Par were between 0.03 and 3.00 crossover, with a mean of 1.26 crossover, and the effect coming from the nonreference parents were between 0.10 and 4.63 crossover, with a mean of 0.84 crossover. Chromosomes 3B and 3A carried the highest number of QTL with effects from the nonreference parents (seven and six QTL, respectively).

QTL were not equally distributed over the genomes. The A genome seemed to be enriched with QTL, 64 fell on this genome. In more detail, 12 QTL were found on chromosome 4A, 11 on 3A and 5A, 8 on 1B, 2A, 3B and 7A, 7 on 6A, 6 on 1A, 4B, 5B, 6B, and 3 and under on the D chromosomes and the other remaining chromosomes.

QTL were projected on the LRC map chromosomes to investigate possible colocalizations. In many cases, the QTL seemed dispersed and not localized in the same region (Figure S10 in File S1). However, in several such cases, QTL from different populations share some of their C.I. markers, *e.g.*, the QTL on chromosome 4A carry markers highly common between populations, with marker BS00049911 found in eight QTL intervals. Similarly, for the QTL on 3A, the markers BS00022862 and BS00074617 are each present in seven QTL intervals. If one would assume that a marker present in a QTL region in at least four different populations would indicate a common QTL, this would define at least 10 common recombination QTL, present on chromosomes 1B, 2A, 3A, 3B, 4A, 4B, 5A, 5B, 6A, and 7A (Figure S10 and Table S9 in File S1).

## Discussion

### Genetic mapping of the NAM panel

We report on a novel bread wheat NAM panel, consisting of a set of 60 biparental hexaploid wheat populations, sharing one reference parent. The nonreference parents were chosen mainly from landrace accessions from the genetically diverse core of the Watkins collection, selected from all nine ancestral populations discovered in wingen *et al.* (2014) (Table S1 in File S1).

Although many factors in the subsequent genotyping and mapping processes were similar, and populations mainly differed in the second parent, automatic mapping functions produced very different outcomes for individual populations, particularly regarding the numbers and lengths of LGs (data not shown). Some LGs consisted of groups of markers previously assigned to different chromosomes, mostly with large gaps between the groups. To deal with these differences in LGs, hand-curating of initial maps was necessary. The multi-chromosome LGs were manually split into LGs following set rules. Furthermore, many cases were observed where markers assumed to be on the same LG did not all link up but fell into several small LGs. Such splits were observed in 341 cases, resulting in two or even three LGs (34 cases) instead of the expected equivalent of one per chromosome. In total, 27% of the expected chromosomes were represented by more than one LG. Splits in LGs seemed to be most frequent in the A genome (mean 32.4%) and least frequent in the B genome (mean 20.1%), and thus not randomly distributed. We see some evidence in the marker order comparisons, *e.g.*, in chromosome 7A, that chromosome splits are linked to regions with an inverse order. In the split LGs, this region will normally be present in total in one or the other part of the chromosome (Figure S4 in File S1). An inversion, present in one parental accession but not the other, will have an influence on the observed recombination fractions between loci. As the mapping program would not normally take this into account, this can lead to LGs not linking up fully. Similarly, a chromosomal translocation present will mean that markers that are normally located on separate chromosomes will be linked, and thus unusual LGs composed of markers from different chromosomes will be observed. We conclude that the unexpected effects observed in the genetic mapping process, the unexpected linkage of different LGs, and the unexpected splits of LGs, can be possibly understood in the light of the high number of rearrangements and translocations detected (see below).

### Consensus mapping

The 60 biparental maps were used to build the LRC map using linear programming. This approach was chosen as the possibly more accurate directed acyclic graphs algorithm for consensus map formation (wu *et al.* 2011) could not resolve the conflicts detected. This highlights the challenge that the analysis of 60 bread wheat genomes poses, as the complexity of a single genome is already high and, where genomes differ, the complexity increases quickly. Using six maps only, wang *et al.* (2014) reported that to build the Wang map, the exclusion of chromosome 2B from one population was necessary, as an alien introgression on that chromosome restricted recombination and complicated map construction. Thus, it is not surprising that a larger and possibly more diverse set of parental accessions would result in considerably more conflicts for the consensus formation.

However, it should be highlighted that the underlying assumption of the linear programming approach is that there is only one correct order. This assumption is not fulfilled, as shown by the analysis of marker order, so the LRC map is an approximation of the majority of orders. In cases where there are alternative map orders, *e.g.*, due to an inversion shared between several accessions, separate consensus maps could be built for each of the alternative orders as this would allow a more accurate description of a particular region. This has successfully been demonstrated for the 5A awn inhibitor locus. A more precise position could be found when maps carrying an insertion in that region were excluded from analysis (S. Collier, personal communication).

### Map length and marker distances

In the majority of cases, variation in map length fell within the expected range. Thus, detailed analysis of individual biparental map lengths seemed to be uninformative.

The comparison of the LRC map to two high-density maps shows that it is significantly shorter, with only 1862 cM. We assume that this could be due to the marker extent not being sufficient to detect all of the recombination, and thus not revealing the full extent of the map. However, an overestimation of the recombination events in the other maps would also be a possibility, particularly as a higher marker density would allow for more genotyping errors, which in turn would increase the map length. Additionally, the consensus mapping program LPmerge, employed for the LRC map, is known for collapsing rather than expanding map distances (endelman and plomion 2014). A map of an eight-founder bread wheat MAGIC population (gardner *et al.* 2015) derived a total length of 5405 cM, with the map length being strongly associated with the number of unique loci. This is much longer than even the Wang map with 3800 cM. The MAGIC founder lines are all elite lines and quite homologous to one another. Recombination between these lines should thus be high, hence the long map length. With the LRC map being a consensus map and capturing 60 very diverse populations, we assume that future high-density genotyping of the founder maps will result in the detection of more recombination and expand the map length to values near the those of the Wang or MAGIC maps.

The comparison of MDRs between the biparental maps and the LRC map seems to be more informative than the map length comparison, as closer distances between common markers are compared. The mean average MDR for each biparental map chromosome *vs.* the LRC map is, in general, near to or slightly greater than one (Figure 2, dotted line at ratio 1:1). Due to the LRC map being slightly compressed, values are in general not below one. The result shows that, overall, the biparental maps are in agreement with the LRC map. In contrast, genotyping errors in combination with the use of MergeMap (wu *et al.* 2011) led to an inflation of genetic distances in the Wang map, which had to been scaled using the Synthetic DH genetic map (wang *et al.* 2014).

Of specific interest were cases where either complete maps or just single LGs showed larger marker distances than the average, indicating a globally or locally higher recombination rate. Several cases at the chromosome level were identified (Figure 2, disregard the D chromosomes due to insufficient marker availability). Chromosome 6A seems to show more general variation in marker distance than other chromosomes. This could hint at the presence of a genetic element controlling recombination rate that is specific for that chromosome. It was shown that recombination greatly increased from the centromere toward the telomeres on chromosome 6A (poursarebani *et al.* 2014), as expected for plants with large genomes. Moreover, it was shown that a recent translocation (6VS:6AL) led to a suppression of recombination rate on 6AL (xie *et al.* 2012), which could be due to the knockout of a locus involved in recombination.

Many LGs showing a larger MDR also show a poor correlation with the LRC LGs, *e.g.*, LG 3B from ParW433 (Table S3 in File S1). Thus, it can be assumed that many increased MDRs were most likely due to chromosomal rearrangements and not to generally higher recombination rates. However, there are chromosomes that show much longer MDRs that do not show a different marker order. These are putative examples of a different recombination rate *e.g.*, ParW273 LG 1D, ParW141 2B, and ParW209 3B (Table S3 in File S1), and would need to be analyzed for this in more detail. None of these examples coincide with a crossover QTL on the same chromosome.

None of the maps showed increased marker distances for all their LGs. This seems to suggest that recombination rate in wheat is not controlled at the genome level but rather at the chromosome or subchromosome level, as increased marker distances were found for single chromosomes.

### Marker order correlation

Whereas the general trend for most maps and most chromosomes is a strong marker order correlation, individual chromosomes in individual populations may show a different order. The least number of such incongruent cases was found for chromosome 7A (Figure S4 and Table S4 in File S1). The only incongruent 7A LG came from ParW281, one of the two populations that were excluded from most analyses as they showed an unexpected allele ratio and were found to be outliers in most analyses. In contrast, the highest number of incongruent LGs (seven) were found for chromosome 6B (Figure S5 in File S1). Chromosome 3B can be seen as an example between these two extremes, in which there were three obvious cases of incongruent LGs (Figure 3D). Moreover, the AvaCad map formed an additional case of different marker order but the Wang map did not (Table S2 in File S1). Most biparental 3B LGs align well to the LRC LG, *e.g.*, ParW209 (Figure 3A). The ParW729 3B LG shows one major rearrangement in comparison to the LRC LG (Figure 3B). Interestingly, the diverging 3B LGs seem to share a similar marker order, as the 3B LG of ParW729 correlates strongly with that of ParW433 ($\rho_{S}=1.0$) and highly with ParW281 ($\rho_{S}=0.8$), but really poorly with the LRC ($\rho_{S}=0.2$). This suggests that one chromosomal rearrangement event gave rise to the marker order changes in W281, W433, and W729. Furthermore, the AvaCad LG 3B was the LG least congruent with the LRC map ($\rho_{s}=0.66,$ Table S2 in File S1). This comparison is well-supported by a large marker number, and an alignment between LGs 3B of LRC and the AvaCad map suggests that more than a simple inversion has given rise to the differing marker order (data not shown). To larger parts, the marker orders are in concordance but for three small sections, which are located differently on the AvaCad LG than on the LRC LG. These observations could be explained by three independent rearrangement events. In contrast, the correlation between LRC 3B and Wang 3B LGs was quite good ($\rho_{s}=0.83$), suggesting that this anomaly is cross-specific.

In general, to understand the rearrangements better, it would be of interest to determine if a common rearrangement is found in several accessions or if there were independent events of rearrangements, one for each accession. A common event is likely when incongruent LGs show a similar order between them and come from the same ancestral group. There are several cases following this pattern, one of which is the example of the incongruent 3B LGs just mentioned. Another case can be found in the 6B LGs, where the LGs from ParW219, ParW324, and ParW680 are highly correlated ($0.94\leq \rho_{s}\leq 1.00$), but not strongly correlated with the LRC LG. Two of the three accessions come from the same ancestral group 1.4 from Europe–Asia (wingen *et al.* 2014). Furthermore, it seems curious that the landrace parents for all three incongruent cases for LG 7D, W034, W352, and W731, also derive from one common ancestral group (1.4). Exceptions to the general consensus marker order might thus be related by a common history and specific consensus maps could be constructed for such subsets. It seems worth mentioning that these named examples were all connected to ancestral group 1.4, with only 20% of our accessions coming from this group. It would be of future interest to investigate if this is due to accessions within this group being more closely related to each other, but less with the rest of the wheat accessions.

### Segregation distortion

Levels of segregation distortion were found to be different between populations of the NAM panel and possibly determined to a larger extent by the genotype of the noncommon parent. The observed percentage of SDL (mean 3.2%) is similar to but slightly lower than others have reported, *e.g.*, marone *et al.* (2012) reported SDL frequencies between 0.6 and 11.8% (P<0.01, no correction for multiple testing) for six durum wheat RIL populations and li *et al.* (2015) found frequencies of 10.4 and 18.5% (P<0.05) in two elite or breeding bread wheat RIL populations. The present analysis found the highest levels of SDL for chromosome 7B (6.88%) followed by 6B (6.47%). A comparison of the frequency of SDL on chromosomes 6B and 7B across all 60 populations shows strong variations in the distribution. It is more evenly distributed for 6B (22 populations have SDL) than for 7B (11 populations have SDL at high levels). This pattern of large variation of SDL frequencies between populations and chromosomes was also observed in other studies. cavanagh *et al.* (2013) identified cases of severe deviation from Mendelian segregation in wheat populations mainly on chromosome 2B in the region of Sr36. Other hot spots were chromosome 1A and smaller regions on chromosomes 4B, 5A, 5B, 6A, 6B, and 7A. li *et al.* (2015) reported two different sets (1B, 6B, and 7A *vs.* 2B and 3B) for two populations, whereas marone *et al.* (2012) named 7B and 6B as the two chromosomes with the highest proportions of skewed markers. Thus, it seems that the distribution and frequencies of SDL are dependent on the specific population analyzed. It would be of interest to test the assumption that the genetic relatedness of the parents may play a role in the amount of SDL detected in a future analysis. High-density genotyping of our NAM panel would provide sufficient statistical power for this. The frequency of SDL per genome was found not to be very different between the homeologous genomes; for the D genome it was lowest with 1.9%, followed by the A genome with 2.6%, and highest for the B genome with 4.2%. This finding agrees with other studies, in that the B genome has the highest number of SDL. Differences are more pronounced at the single marker level where there seem to be hot spots for segregation distortion around some markers (Table S5 in File S1). In general, the direction of significant distortions over several populations is either leaning toward the Par allele or toward the nonreference parents at a single locus, presumably driven by the Par allele being more or less favorable over all other alleles at that locus (Figure 4). Due to different polymorphisms being available in the populations, marker sets on maps only partly overlap. Therefore, it is difficult to verify any similarities in patterns of distortion between maps. However, the detection of marker hot spots supports the assumption that there is a certain degree of similarity. A future high-density genotyping approach may reveal details on how unique or how common SDL are in the NAM panel.

The causative effects of the observed SDL are of importance when planning germplasm improvement by breeders. Seven populations were developed from landrace accessions with winter growth habits. These populations have a slightly higher average percentage of SDL (3.69%) than those developed from spring-type landrace accessions (2.95%). The difference is not significant as numbers are low; however, the two chromosomes with the most differences are 5D and 5B, which carry the vernalization genes *Vrn-D1* and *Vrn-B1*. It seems extremely likely that a selection for spring growth habit in the progenies could have led to an increased ratio of Par alleles at the *Vrn-1* loci and led to segregation distortion at these loci.

Moreover, chromosome 5B has also been shown to contain elements associated with segregation distortion in a targeted analysis of that chromosome (kumar *et al.* 2007). Particular preferential male gamete transfer was the mechanism leading to SDRs. We find for chromosomes 5B and 7B, where the highest numbers of similar translocation types are observed, that the same chromosomal regions seem to be part of the translocation and carry SDL (Figure S8 in File S1). Thus, we hypothesize, that these two effects could be causally linked.

In other cases where no such correlation can be found, *e.g.*, for chromosome 4B where hardly any translocations are present, SDL will be possibly caused by markers linked to a deleterious locus involved in gamete transfer preferences. Even a bias in selection, as found in a large study of *Arabidopsis* F2 populations (salomé *et al.* 2012), could underlie some of the SDL. In the *Arabidopsis* study, an inadvertent selection against late-germinating genotypes in the population development is discussed as the likely cause for SDL in *DELAY OF GERMINATION-1*.

Segregation distortion could also stem from genomic regions where parents have been heterozygous. In the most extreme case, this could have been as much as 50%, if the landrace seed used was a complete hybrid. However, this extreme is not very likely. Furthermore, regions where there are segregation hot spots in several varieties will most likely be due to either shared history, *e.g.*, a common translocation, or the presence of genes promoting segregation distortion like embryo lethal or gametocidal genes.

No large overrepresentation of heterozygotes was observed in the F4 RIL populations, reported by truong *et al.* (2014) as being common for RIL populations from some plant species such as pea and maize. In this large study of wheat, only 15% of the markers showed slightly more heterozygotes than expected. This gives confidence that the produced genetic maps, unlike in pea and maize, are not inflated due to a large overestimation of recombination frequencies.

### Translocation

Reports of chromosomal rearrangements in wheat date back some time and evidence of the abundance of such events is accumulating fast, with increasing gains of genotyping and sequence data on bread wheat and related species. luo *et al.* (2009) reports on the detection of 50 inversions and translocation events between rice, sorghum, and *Aegilops tauschii*, the wheat D genome progenitor, concluding that chromosomal rearrangements were very much part of the formation of the Triticeae family and thus have also played a major role in the history of bread wheat. More signs of chromosomal rearrangements in bread wheat were found by comparing homoeoloci between the CSp genotype shotgun sequence and the genome sequences of *Brachypodium distachyon*, *T. uratum*, and *A. tauschii*. At least 10 wheat chromosomes revealed pericentric inversions (ma *et al.* 2014) and 42 events of interchromosomal rearrangement were found on 18 wheat chromosomes (ma *et al.* 2015), rather scattered across the genome. Additionally, lucas *et al.* (2014) found groups of genes on chromosome 5D that were absent in the syntenic regions of rice or *Brachypodium*, but that were present as a group elsewhere in the genomes of those two species. This supports a large-scale conservation of Triticeae chromosomes, but also suggests that some regions are evolving rapidly through chromosomal rearrangements.

In the report presented here, we detect signs of at least 105 translocations in 42 of the analyzed 60 Watkins landrace accessions, falling into > 60 different translocation classes. This number seems high, given that these are accessions of a domesticated species and that rearrangements must either have happened in the evolutionarily short time of ∼10,000 years since the domestication of bread wheat, or must have introgressed from wild species after domestication, as some gene flow between wild progenitors and domesticated crops seems to be the rule rather than the exception (dvorak 2006).

There is good agreement between the cytological translocation survey (badaeva *et al.* 2007), which found that 30% of analyzed wheat accessions carried visible translocations, and the current report in finding no pattern of class of translocation that would be more likely than others. It seems that chance is a major element in the translocation process. Similarly, ma *et al.* (2015) reports that the diversity of predicted translocation events between CSp bread wheat and other related species seems to point at a basic mechanism that is at least partly random. The present study predicts that an even larger diversity of chromosomal rearrangements will be found as soon as more sequence information on different wheat varieties is available.

chaffin *et al.* (2016) reported on a consensus map for hexaploid oat from 12 biparental populations, from a total of 19 different parental lines. They detected a number of chromosomal rearrangements and speculated that allo-hexaploid plants have mechanisms for “a very high amount of genetic buffering” and can, thus, accommodate wild introgression, duplications, and deficiencies arising from reciprocal and nonreciprocal chromosomal rearrangements. Given the large number of rearrangements and translocations found in this study, it provides further support for the buffering capacity of hexaploid genomes.

For hexaploid wheat, the accessions from Central Asia showed the highest frequency of translocations (43%), also characterized by a broad diversity of translocation types (badaeva *et al.* 2007), coinciding with the center of diversity for bread wheat (vavilov 1992). In accordance with this, we found that accessions coming from ancestral group 1.3 (Central/Eastern Asia) have a very high percentage of translocations (82%), but that the ancestral group 1.4 (Europe–Asia) has the highest percentage (90%).

Supplementary to the enrichment of translocations due to the ancestry of accessions, we found that the chromosomes of the B genome showed a higher number of translocations than those of the A genome (Table S7 in File S1). This could possibly mean that the B chromosomes had more time to accumulate translocations and would thus be older, but it could also mean that the B chromosomes are less stable than the A chromosomes.

### Recombination QTL

Crossover frequency is important for the patterns of genetic variation and relative crossover rates vary between plant genomes as, *e.g.*, increased genome size is associated with reduced crossover frequency (henderson 2012). Little is known about the variation of the genome-wide recombination rates within plant species (bauer *et al.* 2013).

From the genetic maps of the NAM panel, ∼126,300 crossover events were estimated and used as a trait for a crossover or recombination QTL analysis. One hundred and sixteen QTL were found, which could be loci controlling recombination frequencies or hot spots where crossovers are more likely. For 50 QTL, the positive effect came from the nonreference parents. At least on 10 chromosomes, QTL seem to cluster to the same regions. However, the overlap of the C.I. is not always obvious. This might reflect the low statistical power of a QTL analysis of a trait that is established during the process of population development, and will thus have less statistical power than a trait that was measured in the final population (esch *et al.* 2007). To try to increase recombination rates by using alleles from landrace accessions, QTL regions on 2D, 3B, 5A, and 7A seem to be good candidate regions to be evaluated in a near isogenic line crossing program. In support of one of these regions, a recombination QTL was also detected in population “W7984 × Opata 85” on chromosome 3B (esch *et al.* 2007). The region between markers Xtam61 and Xpsr689 spans over the centromere (cM 42.8–107.0 on the “W7984 × Opata 85” map), a broad region but, in line with the location of several QTL in our study, quite close to the centromere.

Better understanding of recombination rate variation will have practical applications by facilitating the construction of particularly stable varieties on the one hand and lines with higher recombination rates on the other. Favorable linkage blocks will be more stable in the former lines with low recombination rates, which would be the aim for commercial varieties. Lines with high recombination rates could be used to break unfavorable linkage blocks during the breeding process. The control of recombination rate changes would be even more valuable if it they could be targeted to specific genomic regions. Interestingly, population ParW044, which showed the highest number of crossover QTL, was also reported as flowering very late, much later than most of the other populations. It would be interesting to find out if being late could give more opportunity for the plants to have crossovers, and thus a higher number of overall crossovers. On the other hand, a higher recombination rate could result in meiosis being slowed down, thus, it would take the plants longer to get to the point of flowering.

### General thoughts

Conducting a large study on 60 populations with big genomes was bound to bring up some unexpected genomic events, if the assumption that the wheat genome shows a high fluidity is correct. As expected, such singular events were observed, and these did not immediately have conclusive explanations. The strange allele distributions in populations ParW281 and ParW313, different from the expected F4 ratios, are part of these observations. Although the higher rate of Par alleles seemed to hint toward a backcross, this would not really explain the allele numbers observed. Another deviation from the expected was observed in the ParSyn (Syn = SS7010073) population. Here, chromosomes 1A and 1B predominantly carried alleles from Par only in a cohort of that large population. The predicted translocation T1A:1B possibly explains low levels of recombination on both chromosomes. Furthermore, one could speculate that the Par 1A and 1B chromosomes were more advantageous than the respective Syn chromosomes and would thus be more successfully transferred to the next generation, but the genomic mechanism of how this would happen to a cohort of the population is not clear. In another population, ParW698, which was for this reason excluded from the analysis, the whole 7B chromosome was completely inherited from W698 only, whereas other chromosomes had the normal mixtures. In the future, more detailed genotypic analyses may reveal more unexpected genomic events and possibly help to find explanations for the mechanisms underlying this fluidity.

An alignment of the LRC map along the CSp genome assembly reveals good overall agreement of marker order (Figure S3 in File S1). It is also obvious that there is low genetic diversity on the D genome, and that the alignments are very sparse in spite of efforts to specifically choose polymorphic D genome markers when available. Interestingly, there seem to be regions of higher diversity and lower diversity along the D chromosomes. Checking if the regions of higher diversity came from specific populations only, it seemed that a large number of populations carried these regions, *e.g.*, 22 populations had four or more markers mapping to the region on LRC chromosome 1D ∼44 cM. This suggests that these regions of higher diversity are quite old and stem either from the origin of wheat or before, rather than stemming from a recent alien introgression. The genetic heterogeneity found also agrees with the uneven distribution of nucleotide diversity per chromosome in the D genome reported by akhunov *et al.* (2010).

Many useful genetic resources have been developed as one outcome of this study. Biparental SSD populations with landrace parents from the Watkins collection together with genetic maps are available to the research and breeding community to mine for genetic diversity in the collection. The populations can be put together and used as a NAM panel if more recombination to identify specific genomic loci is needed. The study has also identified genomic regions in specific populations that might form a barrier for recombination, like SDL and translocations. This information could be consulted in the selection of crossing parents for breeding programs. Moreover, the study has also revealed QTL that influence crossover number and has identified chromosomes in specific populations that seem to have a higher recombination rate. This information can hopefully be used to develop wheat varieties with higher recombination rates, which could help to achieve breeding targets more easily.

## Supplementary Material

Supplemental material is available online at www.genetics.org/lookup/suppl/doi:10.1534/genetics.116.194688/-/DC1.
