# Supplementary material for: Wheat Landrace Genome Diversity
Source: Genetics. 2017 Feb 16;205(4):1657–76. doi: 10.1534/genetics.116.194688 (PMC5378120; doi:10.1534/genetics.116.194688)
Supplement: Supplementary file 1 [file 1657FileS1.pdf]

Wheat Landrace Genome Diversity - Supplementary Material

Luzie U. Wingen[*,1], **Claire West**[*], **Michelle Leverington-Waite**[*], **Sarah Collier**[*], **Simon Orford**[*], **Richard Goram**[*], **Cai-Yun Yang**[†], **Julie King**[†], **Alexandra M. Allen**[‡], **Keith J. Edwards**[‡] **and and Simon Griffiths**[*]

[*]Crop Genetics, John Innes Centre, Norwich Research Park, Norwich, NR4 7UH, Norfolk, UK, [†]Division of Plant and Crop Sciences, School of Biosciences, University of Nottingham, Sutton Bonington, LE12 5RD, UK, [‡]School of Biological Sciences, University of Bristol, Bristol, UK

Literature Cited

Wang, S., D. Wong, K. Forrest, A. Allen, S. Chao, B. E. Huang, M. Maccaferri, S. Salvi, S. G. Milner, L. Cattivelli, A. M. Mastrangelo, A. Whan, S. Stephen, G. Barker, R. Wieseke, J. Plieske, I. W. G. S. Consortium, M. Lillemo, D. Mather, R. Appels, R. Dolferus, G. Brown-Guedira, A. Korol, A. R. Akhunova, C. Feuillet, J. Salse, M. Morgante, C. Pozniak, M.-C. Luo, J. Dvorak, M. Morell, J. Dubcovsky, M. Ganal, R. Tuberosa, C. Lawley, I. Mikoulitch, C. Cavanagh, K. J. Edwards, M. Hayden, and E. Akhunov, 2014 Characterization of polyploid wheat genomic diversity using a high-density 90 000 single nucleotide polymorphism array. Plant Biotechnology Journal **12**: 787–796.

Wilhelm, E. P., R. M. Howells, N. Al-Kaff, J. Jia, C. Baker, M. A. Leverington-Waite, S. Griffiths, A. J. Greenland, M. I. Boulton, and W. Powell, 2013 Genetic characterization and mapping of the Rht-1 homoeologs and flanking sequences in wheat. Theoretical and Applied Genetics **126**: 1321–1336.

Wingen, L., S. Orford, R. Goram, M. Leverington-Waite, L. Bilham, T. Patsiou, M. Ambrose, J. Dicks, and S. Griffiths, 2014 Establishing the a. e. watkins landrace cultivar collection as a resource for systematic gene discovery in bread wheat. Theoretical and Applied Genetics **127**: 1831–1842.

acc code	country	accession name	comment	growth habit	ancestral group
Par	GBR	Paragon	PBI Cambridge; 1998^{wp}	S	-
CSp	CHN	Chinese Spring	Sichuan^{wp}	S	-
Gla	FRA	Glasgow	Saaten Union; 2006^{wp}	W	-
Gar	FRA	Garcia	SECOBRA; 2006^{wp}	W	-
Pam	RUS	Pamyati-Azieva	Sibirskii NIISKH; 2000^{wp}	S	-
Syn	USA	SS7010073	E. Sears; synthetic hexaploid of *Triticum dicoccum* x *Aegilops tauschii* (Wilhelm *et al.* 2013)	W	-

doi: 10.1534/genetics.XXX.XXXXXX
Manuscript compiled: Sunday 25th December, 2016%

[1]Crop Genetics, John Innes Centre, Norwich Research Park, Norwich, NR4 7UH, Norfolk, UK phone: 0044-1603-450-508, luzie.wingen@jic.ac.uk

acc code	country	accession name	comment	growth habit	ancestral group
W007	AUS	-	-	S	2.4
W034	IND	Desi	Etawah, United Provinces; 27N 79E	S	1.4
W044	MAR	-	Granja Agricola, Quebdana, Melilla; 35N 3W	S	2.4
W079	IND	Dolatkhani (white)	United Provinces	W	1.3/2.4
W103	ITA	Gentil Rossa	Flaksberger 19928	S	2.5
W110	FRA	Carré Géant blanc	-	W	2.2
W139	FRA	Bladette de Besplas	-	S	2.5
W141	CHN	-	Mukden	S	2.4
W145	ESP	Aurora	Navarre	S	2.4
W199	IND	Boojri (bearded)	Margnzani, Sibi, Baluchistan; 29N 68E	S	1.4
W209	EGY	Belardi 24B	-	S	1.3
W219	ESP	-	Vallodolid district	S	2.4
W224	CHN	Red wheat	Tsinan district	S	2.4
W238	IRN	-	Mehraban, N Hamadan; 36N 48E	S	1.4
W254	MAR	-	Tazi, Kenitra; 34N 6W	S	2.5
W273	ESP	-	Valverde del Camino, Huelva; 37N 6W	S	1.3
W281	GRC	Deve	Karditza + Trikkala districts of Thessalia	S	1.3
W292	CYP	Asprokoutsoullon	Paphos district.	S	2.3
W299	TUR	-	Seraikeuy	S	1.3
W300	TUR	-	Kirk Agach, 50 miles NE of Izmir; 39N 28E	S	1.3
W308	IRN	-	Rasht, Caspian shore; 38N 50E	S	1.4
W313	BUR	2193/20-12	S. Shan States (Imperial Institute)	-	2.1
W324	CHN	-	Yu Mao Grain Store, Hatamen Street, Peking	W	1.4
W352	YUG	-	Bugojno, Travnik, Bosnia; 44N 18E	S	1.4
W360	YUG	Veliko Hoce	Veliko Hoce	S	1.1
W387	ESP	-	Minorca, southern part of Mahon; 40N 4E	S	2.4
W396	PRT	Trigo Rietti	Golegã	S	2.5
W406	IND	Desi	Hasanganj, Unnao, United Provinces; 27N 81E	S	1.3
W433	IND	Soor Ghanum	Musafirpur, Upper Zhob, Baluchistan; 32N 68E	S	1.4
W440	CHN	-	-	S	1.2
W468	AFG	-	-	S	2.2
W471	AFG	-	-	S	2.1
W475	AFG	-	-	S	1.4
W483	POL	Surka Oscista	-	S	2.5
W546	ESP	Hembrilla alto, fuerta	Navarre	S	2.4
W562	GRC	-	Tripolis district, Peloponese	S	2.3
W566	GRC	-	Pygros district, Peloponese	S	1.3
W591	PRT	Trigo Anafil	Frontiera	S	1.3
W624	BGR	Svalene	Vetovo	W	2.5
W627	IRN	-	Asadabad, SW Hamadan; 34N 48E	S	1.2
W629	IRN	Qizilqin Boghda	Hashdarud, Azerbaijan province; 40N 50E	S	1.2
W651	CHN	-	-	W	2.3
W652	CHN	-	-	W	2.5

acc code	country	accession name	comment	growth habit	ancestral group
W670	POL	Zlotka Miczynskiego	-	S	2.3
W680	ITA	Grano locale	Aquila	S	1.4
W694	IND	Lyallpur 8A	Punjab; 31N 74E	S	1.2
W707	IND	Walaiti (Mastung)	Panjpai, Shorarud, Baluchistan	S	1.2
W722	CHN	-	-	S	2.2
W729	IRN	Gandum-i-Jiruft	Jiruft + Roodbar, Kerman; 30N 58E	S	1.4
W731	IND	Kyo / Gahu (Nepali)	Sikkim; 27N 89E	S	1.4
W740	SUN	-	Siberia	W	2.3
W777	FIN	-	-	S	1.2
W784	ITA	Oberdan	Royal Exp. Sta. Rieti	S	1.2
W811	TUN	-	Maison Carree	S	1.2
W827	CHN	-	Shanghai (Tsao-Ka-Doo)	S	1.2

Table S 1 List of accessions and background information. Watkins hexaploid wheat landrace collection accession codes are indicated by 'W' followed by a three digit number. Replace 'W' by 'WAT 1190' for the original code. Country, accession name and comments on Watkins accession received from `www.seedstor.ac.uk`; ^{wp} from `wheatpedigree.net`; growth habit from spring trials 2010 (Wingen *et al.* 2014), S = spring type, W = winter type; ancestral groups from Wingen *et al.* (2014): 1.1.East-Europe-North-Asia (former USSR), 1.2.China-India, 1.3.Centr-East-Asia, 1.4.Europe-Asia, 2.1.South-Europe-Asia, 2.2.North-Asia, 2.3.East-Europe, 2.4.South-Mediterranean-Africa, 2.5.North-Mediterranean.

Chr	Marker number	Map Length [cM]			MDR to LRC		MCR to LRC		MCR
	LRC	LRC	AvaCad	Wang	AvaCad	Wang	AvaCad	Wang	AvaCad to Wang
1A	142	97.4	109.4	161.4	1.64	1.46	0.92 (79)	0.94 (52)	0.86 (51)
1B	227	100.0	178.2	174.1	1.64	1.70	0.93 (176)	0.98 (87)	0.92 (133)
1D	111	76.5	125.4	209.1	1.57	2.08	0.76 (54)	0.84 (33)	0.72 (204)
2A	137	113.3	233.8	185.5	1.68	1.39	0.94 (77)	0.99 (45)	0.97 (42)
2B	172	113.2	141.5	188.9	1.25	1.53	0.85 (123)	0.91 (56)	0.91 (66)
2D	101	114.5	194.3	152.8	1.17	0.79	0.72 (58)	0.90 (32)	0.35 (100)
3A	118	115.4	183.2	207.3	1.77	1.63	0.93 (86)	0.99 (48)	0.97 (65)
3B	172	134.9	240.5	156.1	1.76	1.10	0.66 (136)	0.83 (72)	0.74 (93)
3D	18	16.9	116.1	166.2	7.94	9.27	0.77 (11)	0.90 (5)	0.89 (20)
4A	94	123.3	156.8	166.7	1.16	1.09	0.96 (75)	0.94 (32)	0.97 (41)
4B	95	102.9	111.2	123.0	2.16	1.10	0.97 (78)	0.97 (46)	0.97 (61)
4D	20	37.3	123.7	170.4	2.63	10.99	0.69 (12)	0.75 (10)	0.87 (10)
5A	146	104.0	203.7	148.5	1.98	1.51	0.96 (92)	0.94 (57)	0.99 (55)
5B	199	118.1	264.8	219.8	2.95	1.99	0.78 (157)	0.93 (67)	0.90 (93)
5D	79	47.5	108.1	224.0	1.72	4.32	0.40 (42)	0.84 (17)	0.51 (67)
6A	171	85.2	166.4	183.4	1.88	3.19	0.86 (142)	0.96 (62)	0.75 (85)
6B	156	93.5	136.7	127.5	1.43	1.37	0.97 (110)	0.95 (58)	0.93 (72)
6D	38	19.3	78.5	161.8	4.34	12.54	0.79 (10)	0.50 (4)	0.74 (23)
7A	143	128.5	219.0	244.2	1.52	1.66	0.99 (112)	0.98 (44)	0.98 (56)
7B	101	79.0	74.1	188.6	1.10	1.82	0.91 (63)	0.98 (45)	0.98 (36)
7D	58	41.4	147.0	241.3	2.77	4.48	0.69 (28)	0.52 (12)	0.91 (30)
sum	2498	1862.1	3312.4	3800.6	-	-	-	-	-
mean	119	88.7	157.7	181.0	2.2	3.2	0.83	0.88	0.85

Table S 2 Characteristics of the landrace consensus map (LRC) LGs. Comparison of LG lengths, marker distance ratios and marker correlation ratios to the AvaCad map (`www.cerealsdb.uk.net`) and the Wang (Wang *et al.* 2014) map are given. Number of markers in common between maps compared are given in brackets. Mean distance ratios over 2.0 are highlighted by a red shading of the cell. Marker order correlation values under 0.8 are highlighted by a blue shading of the cell. .

Map	Number of markers	Mean Marker Distance Ratio	Chromosome	Correlation with LRC
ParGar	7	2.70	1D	0.79
ParW273	10	2.16	1D	0.99
ParW313	8	3.90	1D	-0.50
ParW281	11	1.85	2A	0.22
ParW313	10	3.58	2B	-0.03
ParW141	8	1.85	2B	1.00
ParW313	10	1.94	2D	-0.30
ParW209	32	1.83	3B	0.99
ParW281	18	1.97	3B	0.35
ParW313	11	1.83	3B	0.45
ParW433	12	4.64	3B	0.61
ParW007	10	1.75	3B	1.00
ParW468	25	2.09	5A	0.98
ParW281	16	2.51	5B	0.23
ParW139	7	2.84	5D	1.00
ParW281	9	2.19	6B	-0.10
ParW299	9	24.39	6D	0.17
ParW313	11	1.85	7A	0.91
ParSyn	23	2.61	7B	1.00
ParW624	8	2.09	7B	0.24

Table S 3 List of bi-parental LGs with expanded marker distances as revealed by larger MDR in comparison to the LRC map.

Chromosome	Number of times poor correlation	Number of LGs with > 6 markers
6B	7	50
2A	6	59
5A	5	59
5D	5	24
6A	5	61
1B	4	53
2B	4	57
1A	3	39
3A	3	53
3B	3	58
7B	3	41
1D	2	35
2D	2	48
4A	2	40
5B	2	56
6D	2	2
7D	2	9
7A	1	60
3D	0	4
4B	0	20
4D	0	1

Table S 4 Numbers of bi-parental LGs per chromosome that are incongruent with the LRC map The number of bi-parental population LGs that have a poor correlation to the respective LRC LG as measured by Spearman's rank correlation of marker order. Also, the number of LGs which were included in the analysis are given.

marker name	ratio distorted	CSpPar	GlaPar	PamPar	ParGar	ParSyn	ParW007	ParW034	ParW044	ParW079	ParW103	ParW110	ParW139	ParW141	ParW145	ParW199	ParW209	ParW219	ParW224	ParW238	ParW254
BS00110651_6B	0.21			x					x			x									
BS00084005_5A	0.40																				
BS00022053_7B	0.17						x		x				x								
BS00069132_3B	0.26												x					x			
BS00107316_2A	0.14								x	x					x						
BS00022937_6B	0.17	x																		x	
BS00080621_7B	0.12						x					x	x								
BS00011360_5A	0.75																x				
BS00022780_5B	0.12																				
BS00022803_3B	0.11				x							x									
BS00023109_7A	0.27					x													x		
BS00078124_6A	0.15											x									
BS00085589_6B	0.11			x									x		x						

marker name	ratio distorted	ParW273	ParW292	ParW299	ParW300	ParW308	ParW324	ParW352	ParW360	ParW387	ParW396	ParW406	ParW433	ParW440	ParW468	ParW471	ParW475	ParW483	ParW546	ParW562	ParW566
BS00110651_6B	0.21																	x			
BS00084005_5A	0.40								x	x	x	x		x							
BS00022053_7B	0.17																				
BS00069132_3B	0.26					x				x			x								
BS00107316_2A	0.14													x							
BS00022937_6B	0.17									x	x										
BS00080621_7B	0.12																				
BS00011360_5A	0.75		x												x						
BS00022780_5B	0.12										x										
BS00022803_3B	0.11														x						
BS00023109_7A	0.27													x							
BS00078124_6A	0.15																				
BS00085589_6B	0.11																				

marker name	ratio distorted	ParW591	ParW624	ParW627	ParW629	ParW651	ParW652	ParW670	ParW680	ParW694	ParW707	ParW722	ParW729	ParW731	ParW740	ParW777	ParW784	ParW811	ParW827
BS00110651_6B	0.21																x	x	x
BS00084005_5A	0.40																x		
BS00022053_7B	0.17			x							x								
BS00069132_3B	0.26																		
BS00107316_2A	0.14												x						
BS00022937_6B	0.17																		
BS00080621_7B	0.12									x									
BS00011360_5A	0.75																		
BS00022780_5B	0.12		x			x													
BS00022803_3B	0.11																		
BS00023109_7A	0.27																		
BS00078124_6A	0.15								x			x							
BS00085589_6B	0.11																		

Table S 5 Summary of markers showing segregation distortion in more than two populations. Abbreviation of population names as in Table 1. An ‘x’ in the table indicates that a marker is distorted in that population. The presence of a chromosome name means that the maker is distorted, but located on that chromosome.

Accession	Translocations detected in			Comment on GISH
	genetic map	estRF (LOD, no of markers)	GISH	
W103	BS00097307_2A at 80cM end of 2D	-	TA/B:D	detected
	-	T5B:7B (7, 8)	-	TB:B not visible
	-	T2A:3A (7, 3)	-	TA:A not visible
W139	BS00077039_6A at 0cM of 1D	-	TA:D	detected
	-	T1B:1D (18, 5)	-	not detected; maybe complex 1D events?
W308	-	T3B:5A (22, 8)<br>5A 0-19cMs link to 3B 24-43cMs	TA:B	detected
	-	T4B:5B (21, 8)	-	TB:B not visible
W360	-	T5D:6B (6, 1)	-	not detected; below threshold?
	-	-	missing B	
W468	-	T5A:5B (22, 2)<br>single 5B marker in middle of 5A at 70cM	-	not detected; too short?
W546	-	several (< 6)	-	not detected; below threshold?
W624	-	T1D:6B (8, 1)<br>4B (0-6cM) linked to 6D (35-39cM)	TA/B:D	detected
W680	-	T5D:6B (10, 1)<br>BS00009821-BS00022937 link	TA/B:D	detected
W740	-	T5B:5D (21, 3)<br>BS00022999_5B(0cM)-IS00037384_5D(0cM)	TD:B	detected
W827	T5B:7B	-	-	TB:B not visible
	-	T1B:6B (9, 8)	-	TB:B not visible

Table S 6 Comparison of translocations detected by mapping, predicted by estRF analysis and detected by GISH. Ten accessions were tested. In cases where the translocation was short it was not always possible to discriminate the color in the GISH and thus there is some ambiguity if translocations came from the A or from the B genome. Abbreviations: T = Translocation, TA/B:D = Translocation of either A or B with D.

acc	1A:					1B:				1D:					2A:				2B:				2D:		3A:	3B:				4A:		4B:		4D:		5A:		5B:		5D:		6A:	6B:	7A:	acc
name	1B	1D	6A	6D	7B	1D	2B	5B	6B	5D	6A	6B	6D	7D	2B	2D	3A	7B	2D	3B	4D	7B	6D	7A	3B	4B	4D	5A	7B	6B	7A	5B	6D	5A	7D	5B	6D	5D	7B	6B	7B	6B	6D	7B	sum
CSp																															x														1
Pam																																				x[a]									1
Gar				x									x																										x						3
Syn	x[b]														x									x	x						x					x									6
W007																																							x						1
W034								x[a]						x	x										x									x								x	x	x	8
W044																																					x		x[c]						2
W079																											x												x[c]						2
W103													x			x			x																										3
W110																				x																		x	x						3
W139						x					x																																		2
W145																																							x						1
W199																										x																			1
W209																						x[c]																							1
W238						x																																							1
W254																																							x[c]						1
W273																																							x[c]						1
W299																													x	x					x										3
W300																																						x	x						2
W308													x															x[c]				x[c]							x						4
W324																																						x							1
W352															x				x											x														x	4
W387																																							x[c]						1
W468			x																x											x						x[b]									4
W471													x		x			x																								x[c]			4
W475		x			x																			x																					3
W546						x									x									x																					3
W566																								x															x						2
W591						x																																	x						2
W624										x		x[c]																					x												3
W627																													x												x				2
W629															x						x								x													x			4
W670							x																																						1
W680																																								x[c]					1
W694									x[c]																														x						2
W707															x														x	x												x			4
W729																			x[c]																										1
W731						x																	x						x										x						4
W777						x																		x					x										x			x			5
W784																																							x						1
W811																	x												x	x															3
W827									x[c]																				x										x						3
occur	1	1	1	1	1	6	1	1	2	1	1	1	4	1	7	1	1	1	4	1	1	1	1	5	2	1	1	1	8	5	2	1	1	1	1	3	1	3	18	1	1	5	1	2	105

Table S 7 Translocation types found in the NAM panel Of 58 bi-parental genetic maps 42 showed signatures translocations. Par is reference parent for all populations. A detected translocation is noted as 'x' in the table underneath the translocation type. For two translocations predicted from the maps the reciprocal translocation were found (indicated by [a]). Two translocations were predicted by genetic mapping and the estRF plots (indicated by [b]). and 15 translocations were predicted from the estRF plot alone (indicated by [c]). The number of translocations per line are summed up on the right and per type at the bottom. Abbreviations: TL = translocation, acc = accession, occur = occurrence.

Population	Chr	Pos	LOD	%Var	Add.Eff	Nearest Marker
ParW044	1A	42.7	3.0	2.9	-2.2	BS00076668
ParW139	1A	3.7	3.0	11.9	-1.2	BS00023182
ParW300	1A	19.2	2.6	11.2	-1.6	BS00062783
ParW440	1A	57.0	3.3	13.6	1.2	BS00084986
ParW475	1A	15.0	2.7	8.8	-0.3	BS00035720
ParW729	1A	103.8	2.9	9.9	-1.7	BS00010039
PamPar	1B	1.0	3.1	12.2	1.2	BS00022874
ParW044	1B	0.0	13.8	18.0	-3.0	BS00022609
ParW103	1B	18.0	2.0	6.1	1.0	BS00022778
ParW238	1B	54.0	2.4	11.5	-1.8	BS00078414
ParW324	1B	29.0	2.2	9.3	-0.1	BS00022775
ParW396	1B	1.0	4.2	14.4		BS00080214
ParW468	1B	1.0	4.1	14.3	-1.1	BS00011360
ParW475	1B	10.0	2.8	9.3	-0.8	BS00082143
ParW777	1B	83.0	2.2	9.3	1.5	BS00078414
ParW433	1D	2.0	4.0	12.6	0.7	BS00055737
ParW624	1D	30.0	2.6	10.3	-1.7	BS00079462
ParSyn	2A	44.0	2.0	3.1	0.2	BS00008805
ParW034	2A	40.0	4.8	16.8	1.4	BS00001108
ParW044	2A	45.0	4.1	4.1	-1.5	BS00001108
ParW141	2A	24.6	4.0	14.9	0.5	BS00024921
ParW300	2A	0.0	2.4	10.0	-0.6	BS00008805
ParW440	2A	21.3	2.9	11.8	-0.5	BS00078489
ParW627	2A	20.0	2.9	7.7	0.7	BS00059111
ParW827	2A	55.0	2.2	10.4	-1.3	BS00023083
ParGar	2B	65.0	2.9	3.4	0.3	BS00059315
ParW034	2B	4.0	4.3	15.0	-0.6	BS00011388
ParW308	2B	0.0	2.3	9.2	-1.3	BS00023068
ParW044	2D	29.0	5.3	5.5	-2.1	BS00043985
ParW433	2D	54.0	4.7	15.3	0.9	BS00021950
ParW652	2D	88.1	2.4	11.0	0.7	BS00021950
ParW034	3A	24.7	2.0	6.7	1.1	BS00010414
ParW044	3A	2.0	11.7	14.5	-2.2	BS00022844
ParW103	3A	46.0	2.2	6.6	1.1	BS00022862
ParW209	3A	101.0	4.5	12.8	0.8	BS00022862
ParW254	3A	113.7	2.8	12.7	1.2	BS00022410
ParW396	3A	67.1	2.3	7.5	-0.8	BS00076631
ParW546	3A	0.0	2.1	9.7	-1.2	BS00022844
ParW562	3A	99.0	3.1	13.4	0.5	BS00022092
ParW566	3A	94.0	2.3	10.2	-0.9	BS00022735
ParW680	3A	35.3	2.6	9.6	1.1	BS00022844
ParW729	3A	13.3	2.7	9.0	-2.4	BS00009805
ParW044	3B	38.2	5.4	5.7	-2.2	BS00089954
ParW103	3B	0.0	3.3	10.2	0.2	BS00023186
ParW110	3B	134.0	5.0	16.8	4.6	BS00089954
ParW209	3B	31.3	3.0	8.4	1.9	BS00011373
ParW406	3B	48.0	2.2	9.2	0.6	BS00060073
ParW471	3B	28.0	3.3	12.5	0.7	BS00030651
ParW680	3B	42.0	2.8	10.7	0.7	BS00060073
ParW722	3B	16.0	4.0	13.1	0.2	BS00022315
ParW141	3D	40.0	3.1	11.1	-1.0	BS00037276
ParW219	3D	6.0	2.3	7.5	-0.8	BS00082501
ParGar	4A	18.0	2.6	3.1	0.1	BS00035322
ParW044	4A	0.0	2.2	2.2	-2.5	BS00065607
ParW079	4A	46.0	2.5	8.5	-0.8	BS00088726
ParW219	4A	100.0	2.1	6.8	-0.8	BS00048067
ParW299	4A	0.0	3.2	11.1	-0.8	BS00065863
ParW387	4A	26.0	3.4	13.8	0.7	BS00049911
ParW433	4A	5.0	2.0	6.1	0.2	BS00036472
ParW468	4A	69.1	2.4	8.0	-1.0	BS00018651
ParW627	4A	69.0	4.3	11.9	-0.1	BS00043286
ParW707	4A	46.0	2.1	8.9	-1.4	BS00060097
ParW722	4A	8.0	3.7	12.0	-1.9	BS00021989
ParW784	4A	0.0	2.0	9.3	-1.1	BS00065863
ParW044	4B	20.4	9.6	11.3	-1.9	BS00094771
ParW110	4B	42.3	2.6	8.2	0.8	BS00022793
ParW199	4B	8.4	3.1	13.4	-1.6	BS00067242
ParW209	4B	27.0	2.9	8.0	0.7	BS00010015
ParW566	4B	7.0	2.1	9.2	0.8	BS00044374
ParW627	4B	35.0	2.3	6.1	0.6	BS00042105
ParW629	4D	7.5	2.3	8.9	-1.2	BS00068013
GlaPar	5A	13.0	2.8	7.1	0.2	BS00022191
ParGar	5A	39.0	3.0	3.6	-0.5	BS00029413
ParW007	5A	4.3	2.4	10.9	0.3	BS00110139
ParW044	5A	69.0	8.5	9.6	-1.9	IS00027559
ParW079	5A	61.0	2.2	7.7	0.8	BS00022644
ParW139	5A	19.1	2.7	10.7	-1.2	BS00066143
ParW209	5A	11.6	4.7	13.5	2.0	BS00028802
ParW468	5A	242.6	2.1	6.9	0.2	BS00047751
ParW475	5A	70.0	4.6	16.1	-1.3	BS00022891
ParW591	5A	18.0	3.1	13.2	0.7	BS00021953
ParW627	5A	49.0	4.0	11.2	-1.4	BS00075595
ParW044	5B	0.0	6.0	6.4	-2.4	BS00023072
ParW308	5B	44.5	3.9	16.5	-0.4	BS00065729
ParW360	5B	20.0	2.1	9.9	-1.1	BS00040784
ParW471	5B	28.6	3.0	11.0	0.5	BS00084528
ParW627	5B	37.8	3.0	8.2	-0.9	BS00084528
ParW731	5B	75.4	2.6	12.0	-1.0	BS00022405
PamPar	6A	2.0	2.6	10.3	-1.0	BS00023192
ParSyn	6A	16.5	2.6	4.0	0.7	BS00022951
ParW079	6A	8.0	4.7	17.2	-0.4	BS00022628
ParW299	6A	3.0	2.4	8.2	-1.2	BS00081386
ParW308	6A	4.0	2.9	12.2	0.4	BS00105466
ParW722	6A	13.0	3.4	10.8	-1.3	BS00023192
ParW811	6A	0.0	2.0	7.6	1.0	BS00083630
ParW034	6B	60.0	3.0	10.0	-0.0	BS00004016
ParW044	6B	0.0	7.0	7.7	-2.1	BS00067590
ParW110	6B	122.0	2.0	6.4	-0.8	BS00077844
ParW145	6B	0.0	3.0	13.5	-0.2	BS00085589
ParW219	6B	3.3	3.5	11.5	1.2	BS00081608
ParW352	6B	19.5	2.0	7.0		BS00011365
ParW433	6B	13.0	3.4	10.5	0.1	BS00049941
ParW044	6D	16.6	7.0	7.7	-2.5	BS00059791
ParW273	6D	0.0	3.5	14.1	-1.8	BS00042153
ParW483	6D	3.0	3.0	13.7	1.0	IS00007973
ParSyn	7A	70.7	4.2	6.7	1.0	BS00022049
ParW044	7A	52.0	2.6	2.6	-2.6	BS00023109
ParW352	7A	43.0	2.7	9.9	-1.3	BS00024786
ParW387	7A	82.0	2.5	9.9	-1.1	BS00071736
ParW396	7A	69.0	4.9	16.9	-0.5	BS00063460
ParW468	7A	7.0	3.3	11.3		BS00110283
ParW627	7A	53.0	3.2	8.7	0.6	BS00021964
ParW722	7A	13.0	3.1	9.9	1.2	BS00025521
ParW811	7A	76.0	3.1	12.1	0.1	BS00023128
ParW209	7B	43.0	4.1	11.8	-2.0	BS00010355
ParW475	7B	8.0	2.3	7.5	0.2	BS00064146
ParW624	7B	6.0	2.7	10.7	-0.9	BS00049372
ParW707	7D	49.0	2.0	8.5	0.6	BS00022312
ParW811	7D	76.9	2.7	10.3	-0.9	BS00098027

Table S 8 Summary table of QTL detected for the trait crossover count Abbreviation of population names as in Table 1. Abbreviations: Chr='Chromosome', Pos='Position', %Var='Variation explained', Add.Eff='Additive Effect'.

chr	marker name	occur	populations
1B	BS00022101	4	PamPar, ParW396, ParW475, ParW777
1B	BS00022539	4	ParW103, ParW238, ParW475, ParW777
1B	BS00074911	3	ParW044, ParW324, ParW475
1B	BS00099829	3	ParW396, ParW475, ParW777
2A	BS00008805	5	ParSyn, ParW034, ParW300, ParW627, ParW827
2A	BS00059111	5	ParW044, ParW300, ParW440, ParW627, ParW827
2A	BS00078489	5	ParW034, ParW300, ParW440, ParW627, ParW827
2A	BS00001108	4	ParW034, ParW044, ParW440, ParW827
2A	BS00027847	4	ParW141, ParW440, ParW627, ParW827
2A	BS00107316	4	ParW044, ParW440, ParW627, ParW827
2A	BS00019094	3	ParW044, ParW300, ParW440
2A	BS00022995	3	ParW300, ParW440, ParW827
2A	BS00023214	3	ParSyn, ParW440, ParW627
2A	BS00056232	3	ParW044, ParW440, ParW627
3A	BS00022862	7	ParW034, ParW103, ParW209, ParW254, ParW396, ParW546, ParW566
3A	BS00074617	7	ParW034, ParW044, ParW103, ParW254, ParW396, ParW546, ParW566
3A	BS00022410	5	ParW209, ParW254, ParW546, ParW562, ParW566
3A	BS00024548	5	ParW034, ParW103, ParW209, ParW546, ParW566
3A	BS00070014	5	ParW103, ParW254, ParW396, ParW546, ParW566
3A	BS00022844	4	ParW044, ParW254, ParW546, ParW680
3A	BS00023087	4	ParW254, ParW396, ParW546, ParW566
3A	BS00025739	4	ParW254, ParW396, ParW680, ParW729
3A	BS00041121	4	ParW103, ParW396, ParW546, ParW680
3A	BS00022735	3	ParW254, ParW562, ParW566
3B	BS00023030	5	ParW044, ParW209, ParW406, ParW471, ParW680
3B	BS00022403	4	ParW209, ParW406, ParW471, ParW680
3B	BS00022961	4	ParW103, ParW209, ParW406, ParW680
3B	BS00022315	3	ParW209, ParW406, ParW722
3B	BS00022803	3	ParW110, ParW209, ParW722
3B	BS00060073	3	ParW209, ParW406, ParW680
3B	BS00069132	3	ParW406, ParW471, ParW680
3B	BS00089954	3	ParW044, ParW110, ParW471
4A	BS00049911	8	ParW044, ParW079, ParW219, ParW387, ParW433, ParW627, ParW707, ParW784
4A	BS00065607	7	ParW044, ParW079, ParW219, ParW299, ParW387, ParW627, ParW784
4A	BS00060097	6	ParW219, ParW387, ParW433, ParW627, ParW707, ParW784
4A	BS00021989	5	ParW219, ParW299, ParW627, ParW707, ParW722
4A	BS00088726	4	ParW079, ParW219, ParW299, ParW707
4A	BS00101512	4	ParW219, ParW299, ParW707, ParW722
4A	BS00048067	3	ParW219, ParW299, ParW722
4A	BS00065863	3	ParW219, ParW299, ParW784
4B	BS00040517	4	ParW044, ParW110, ParW209, ParW566
5A	BS00021953	6	GlaPar, ParGar, ParW007, ParW139, ParW468, ParW591
5A	BS00021955	5	GlaPar, ParGar, ParW079, ParW139, ParW591
5A	BS00110139	4	ParW007, ParW044, ParW468, ParW591
5A	BS00022891	3	ParW468, ParW475, ParW627
5A	BS00029413	3	GlaPar, ParGar, ParW468
5A	BS00043676	3	ParW209, ParW468, ParW475
5A	BS00079069	3	GlaPar, ParGar, ParW139
5B	BS00047751	4	ParW360, ParW471, ParW627, ParW731
5B	BS00106043	4	ParW044, ParW308, ParW360, ParW731
5B	BS00022780	3	ParW360, ParW471, ParW731
5B	BS00022999	3	ParW360, ParW471, ParW731
6A	BS00081386	5	PamPar, ParW299, ParW308, ParW722, ParW811
6A	BS00105466	5	ParW079, ParW299, ParW308, ParW722, ParW811
6A	BS00023119	4	ParW079, ParW308, ParW722, ParW811
6A	BS00022628	3	ParW079, ParW722, ParW811
6A	BS00023192	3	PamPar, ParSyn, ParW722
6A	BS00054054	3	ParW079, ParW308, ParW722
6A	BS00061296	3	ParW079, ParW308, ParW722
6B	BS00010420	3	ParW468, ParW034, ParW352
6B	BS00067590	3	ParW044, ParW219, ParW433
6B	BS00081608	3	ParW034, ParW219, ParW352
7A	BS00031028	5	ParW044, ParW396, ParW468, ParW627, ParW811
7A	BS00025521	4	ParW352, ParW468, ParW627, ParW722
7A	BS00033613	3	ParW044, ParW387, ParW627

Table S 9 Markers present in at least three confidence intervals of the crossover count QTL Abbreviation of population names as in Table 1 (Wingen et al. 2016) Abbreviations: chr = Chromosome.

Figure S 1

Figure S 1: **Diagrams of the marker density and distribution of 60 bi-parental genetic linkage maps.** The name of the bi-parental population is given on the top left corner of each diagram. Vertical bars below depict LGs of the map, with the name of the LG given on top. Horizontal ticks on the bars refer to marker positions. The spacing of the ticks reflect the genetic distances of the markers. A centimorgan scale is given on the left hand side of each map. Abbreviation of population names as in Table 1.

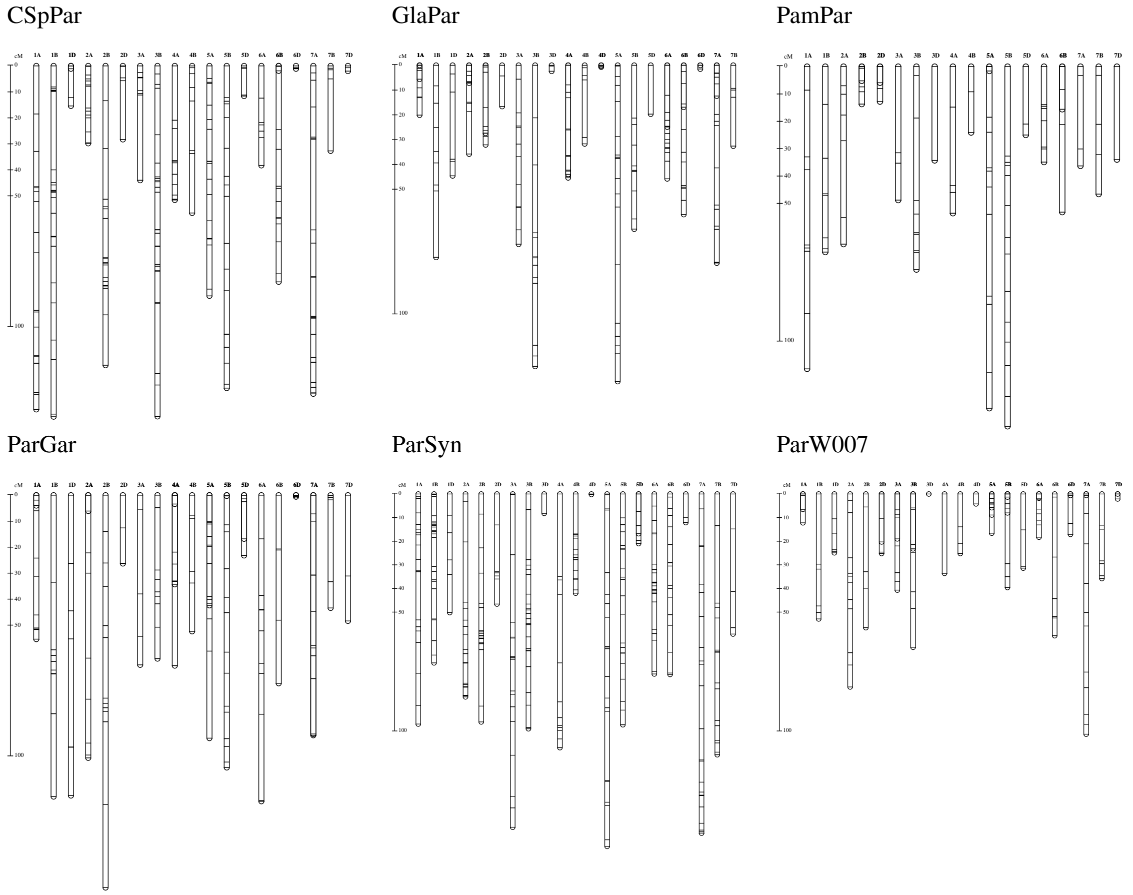

ParW034

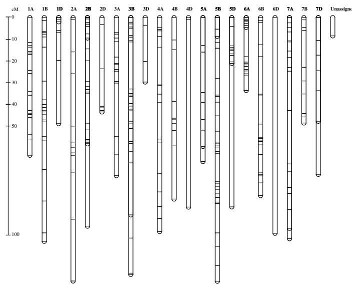

ParW044

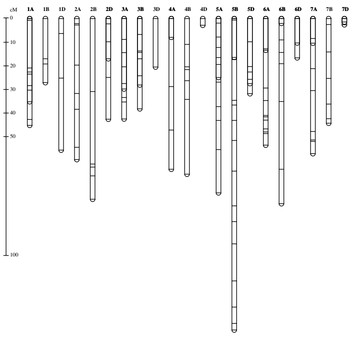

ParW079

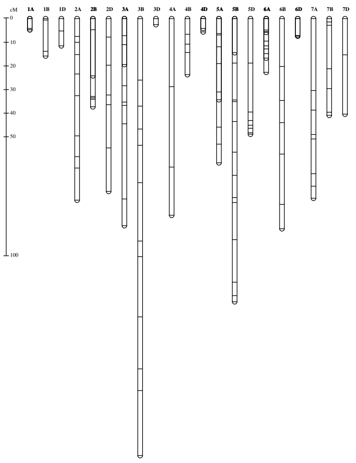

ParW103

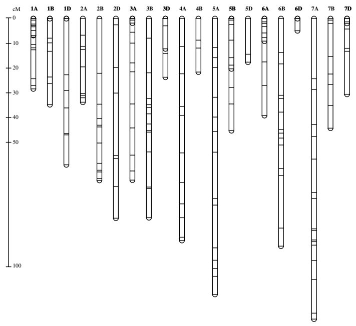

ParW110

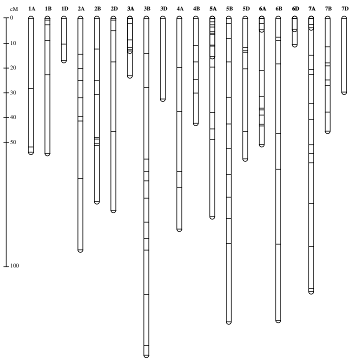

ParW139

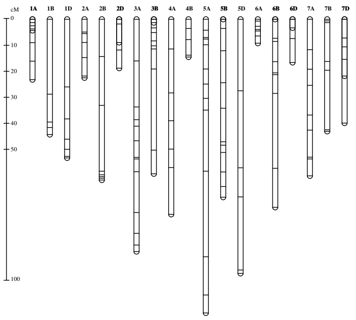

ParW141

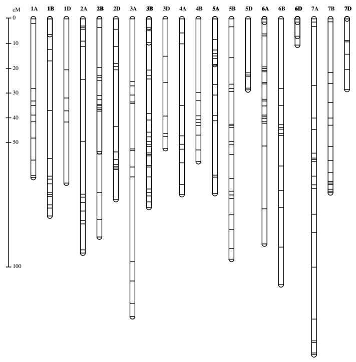

ParW145

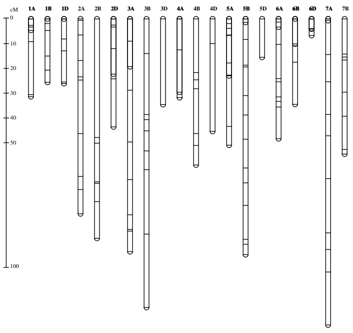

ParW199

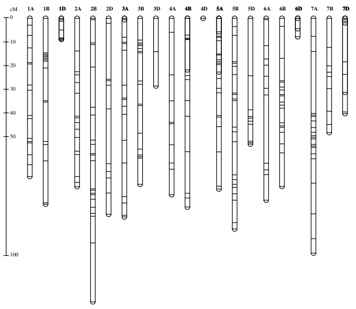

ParW209

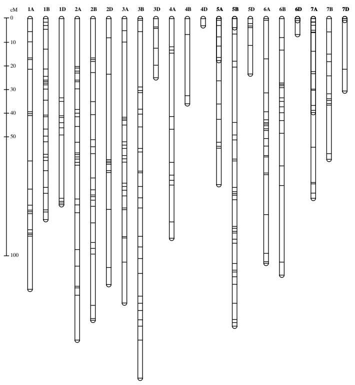

ParW219

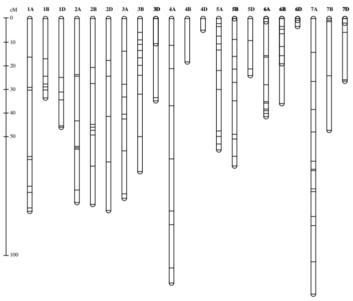

ParW224

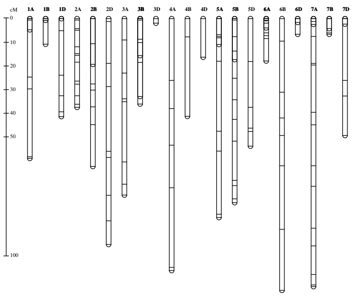

ParW238

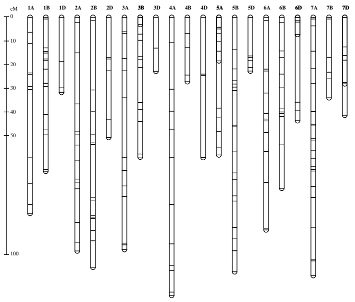

ParW254

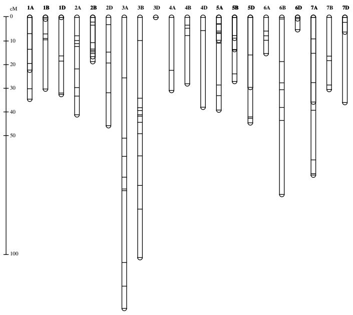

ParW273

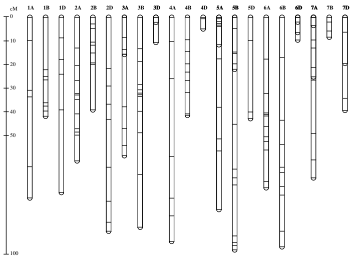

ParW281

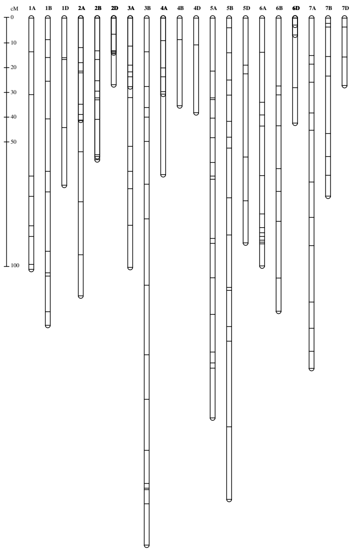

ParW292

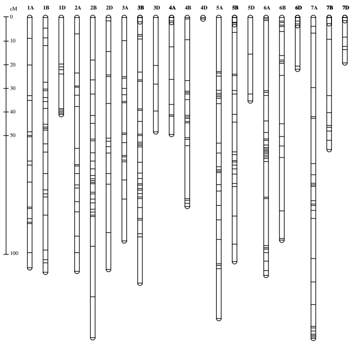

ParW299

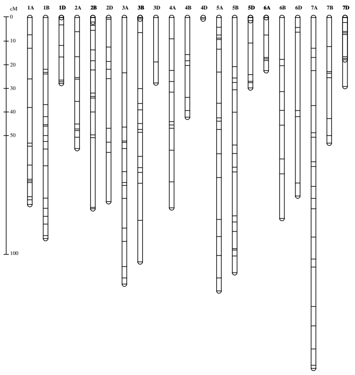

ParW300

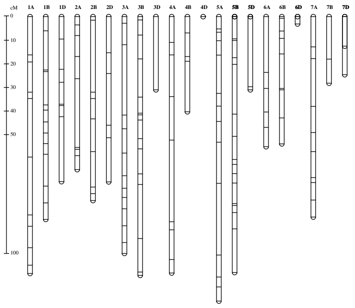

ParW308

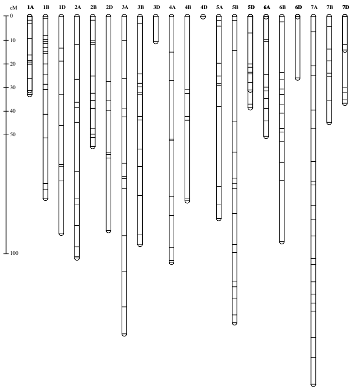

ParW313

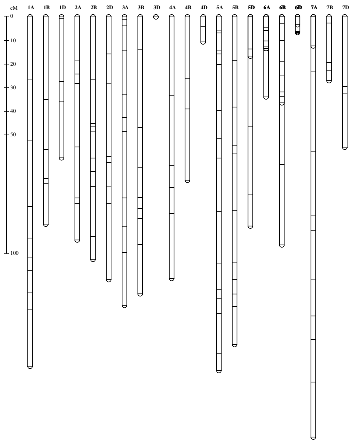

ParW324

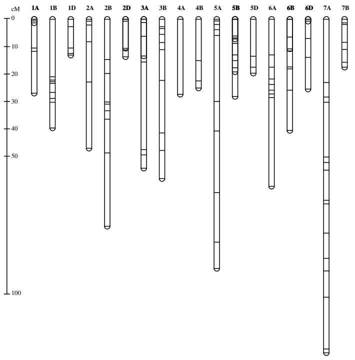

ParW352

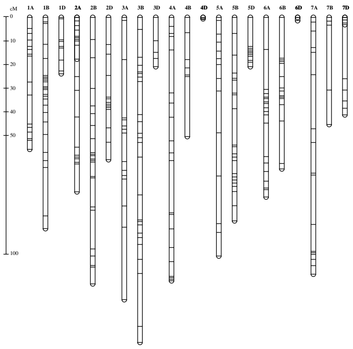

ParW360

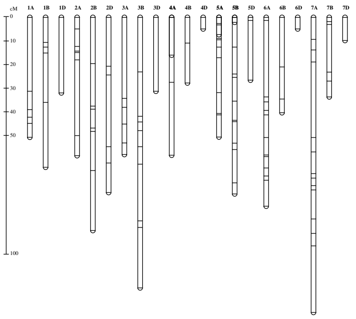

ParW387

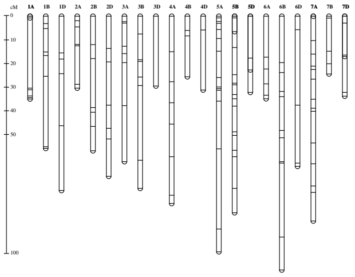

ParW396

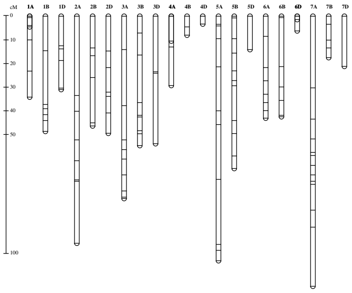

ParW406

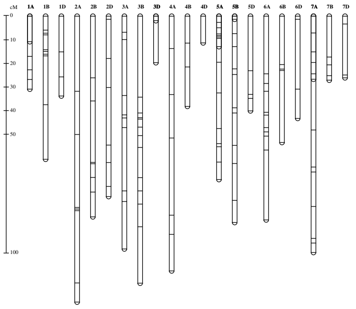

ParW433

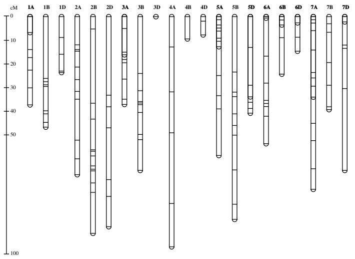

ParW440

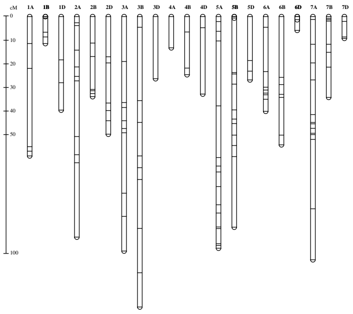

ParW468

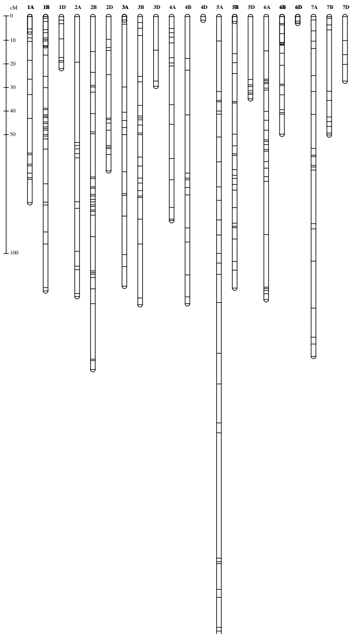

ParW471

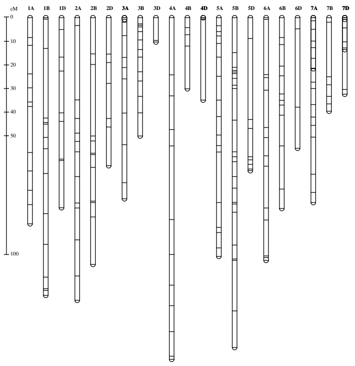

ParW475

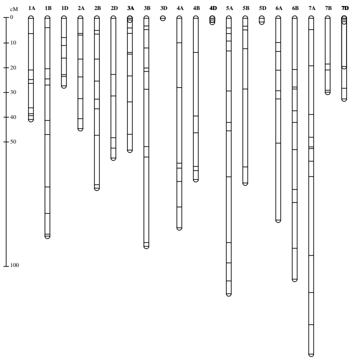

ParW483

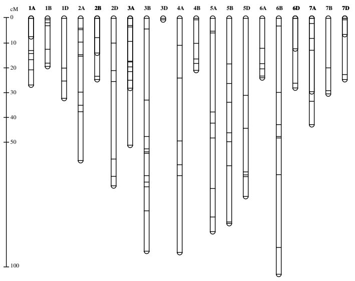

ParW546

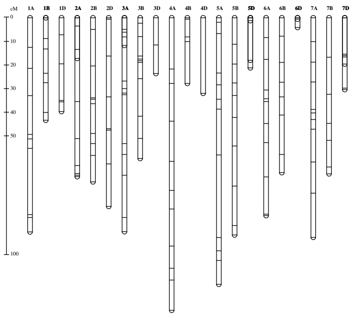

ParW562

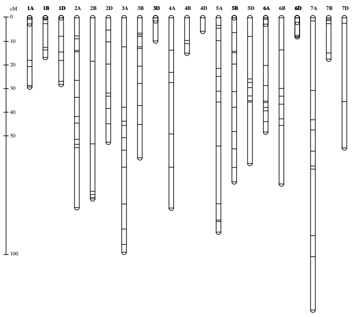

ParW566

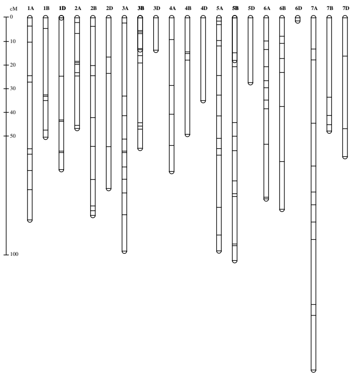

ParW591

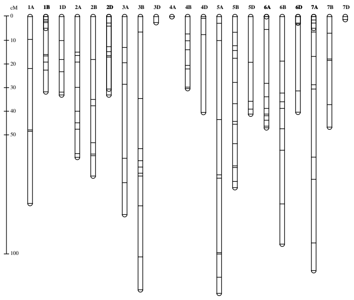

ParW624

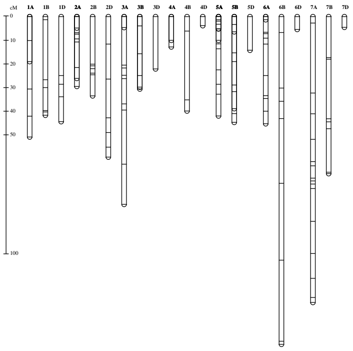

ParW627

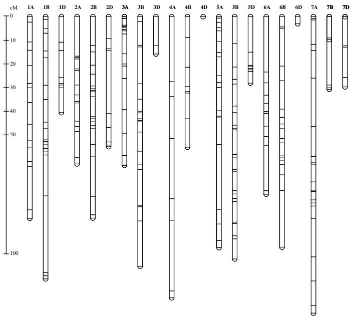

ParW629

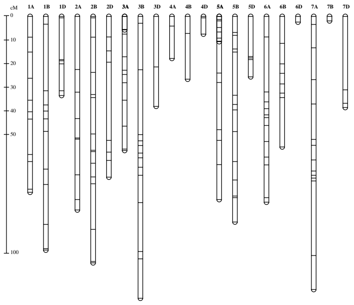

ParW651

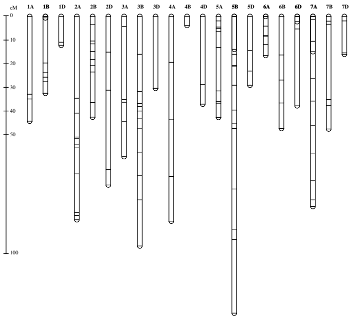

ParW652

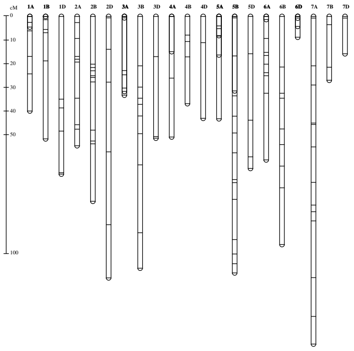

ParW670

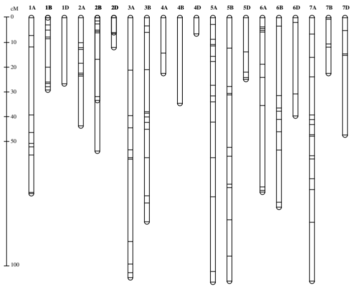

ParW680

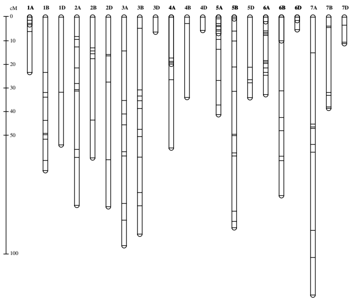

ParW694

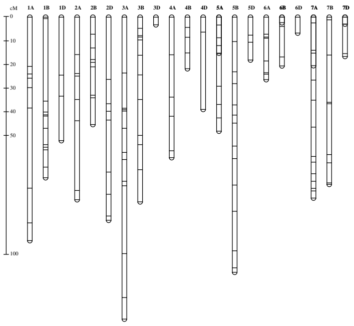

ParW707

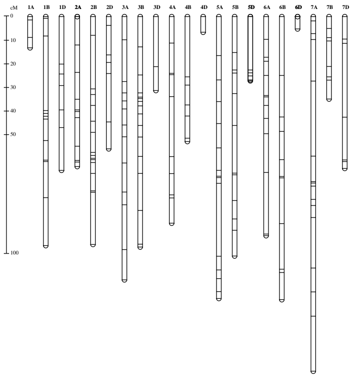

ParW722

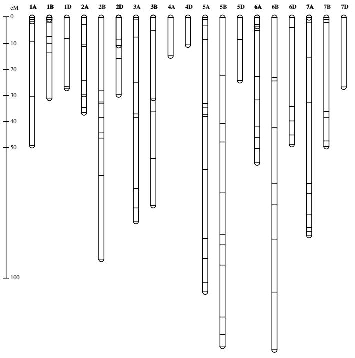

ParW729

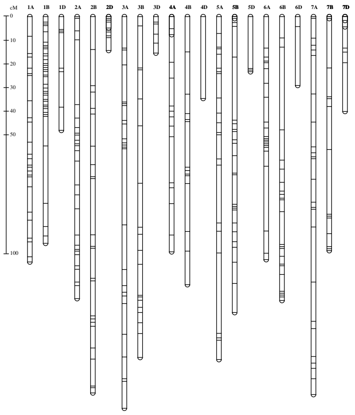

ParW731

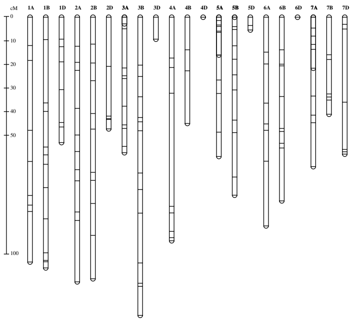

ParW740

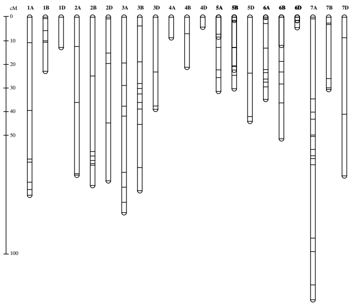

ParW777

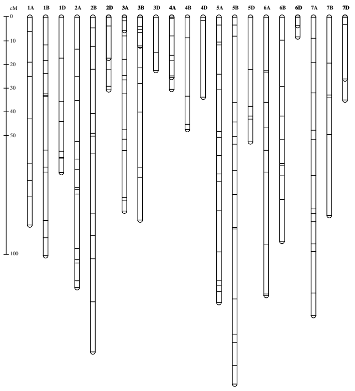

ParW784

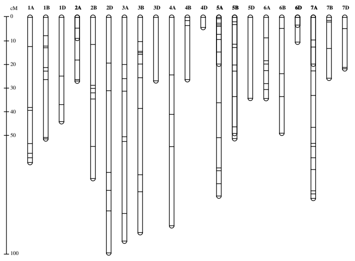

ParW811

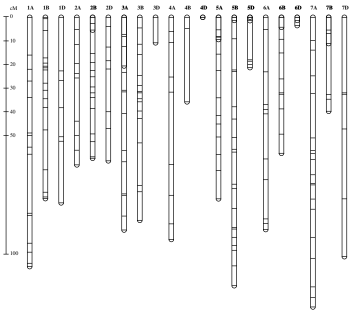

ParW827

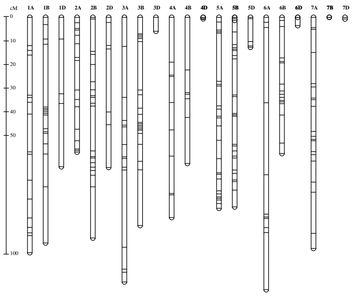

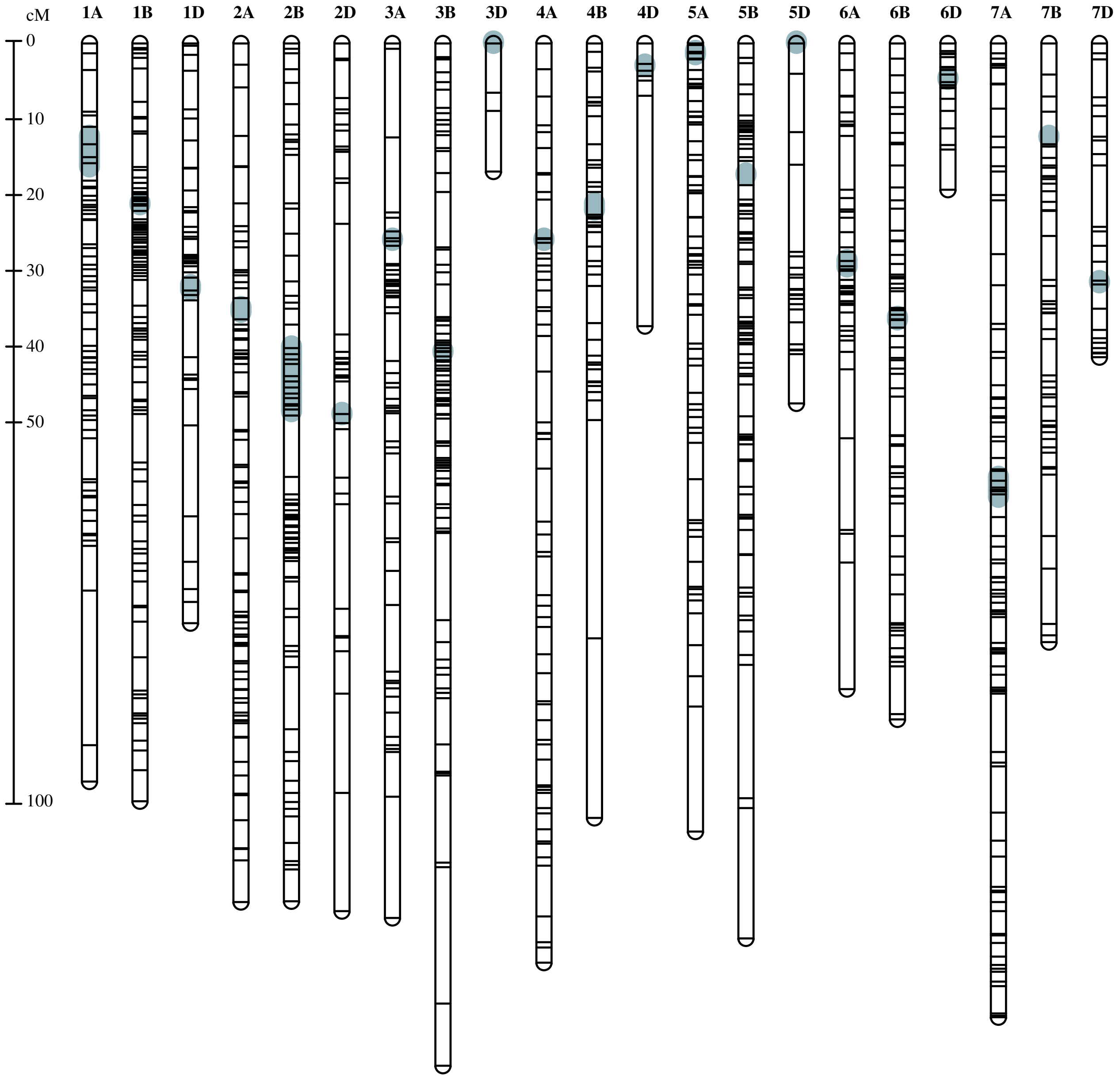

Figure S 2 LRC map marker density. Diagram of the distribution and density of markers on the LRC map constructed from 60 bi-parental population maps. Vertical bars depict LGs of the map, with the name of the LG given on top. Horizontal ticks on the bars refer to marker positions. The spacing of the ticks reflect the genetic distances of the markers. Centromere regions are indicated by a light blue shading. A centimorgan scale is given on the left hand side of each map.

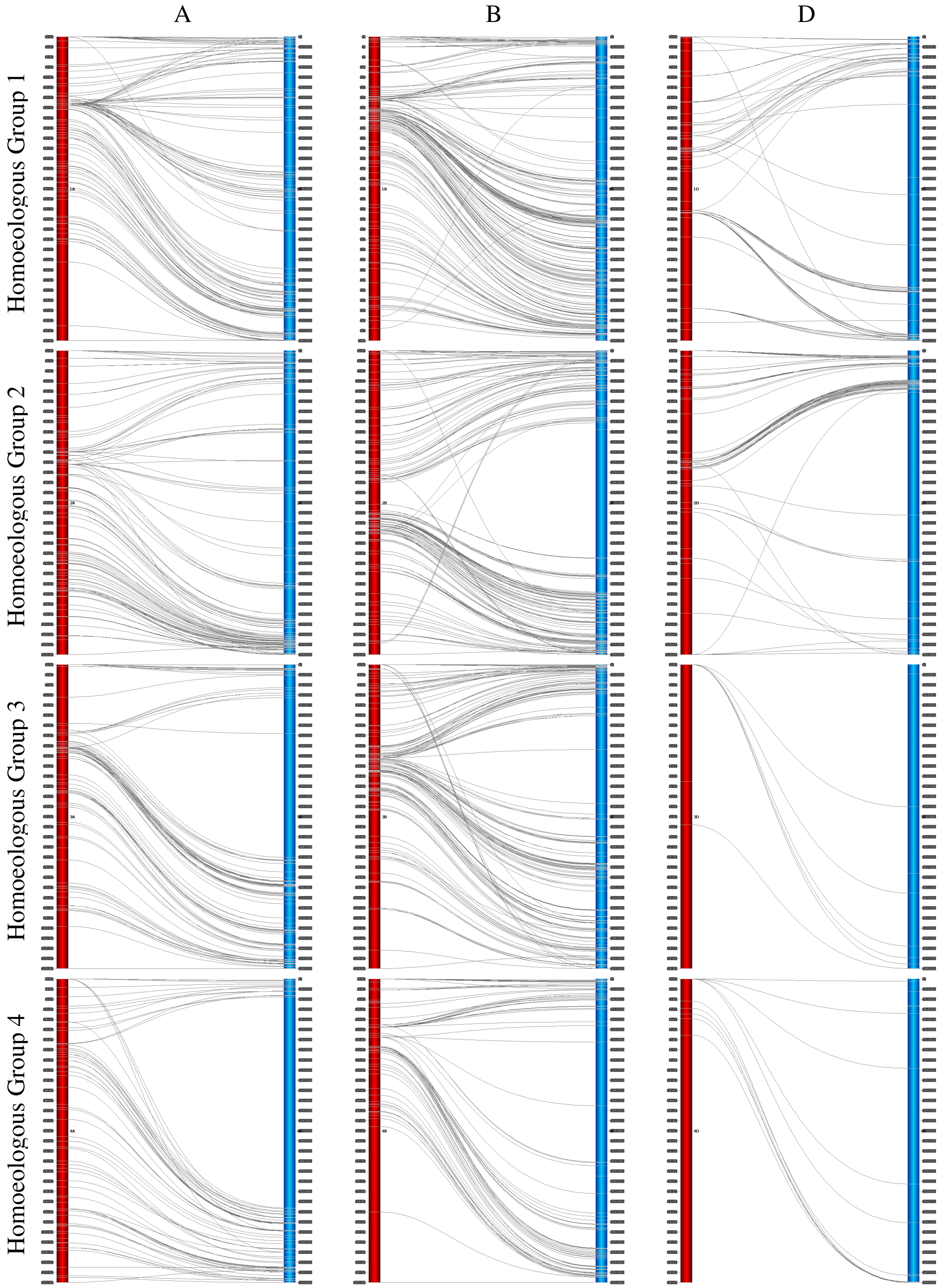

Figure S 3 The LRC map aligned along the wheat genome assembly. Alignment of all chromosomes of the LRC map with the respective chromosome of a recent wheat genome assembly. Alignments depicted as `Strudel` plots with vertical bars representing the chromosomes. (LRC: blue, genome assembly: red). Markers shared between chromosomes are connected. Positions of markers are given in gray boxes at the sides of the bars.

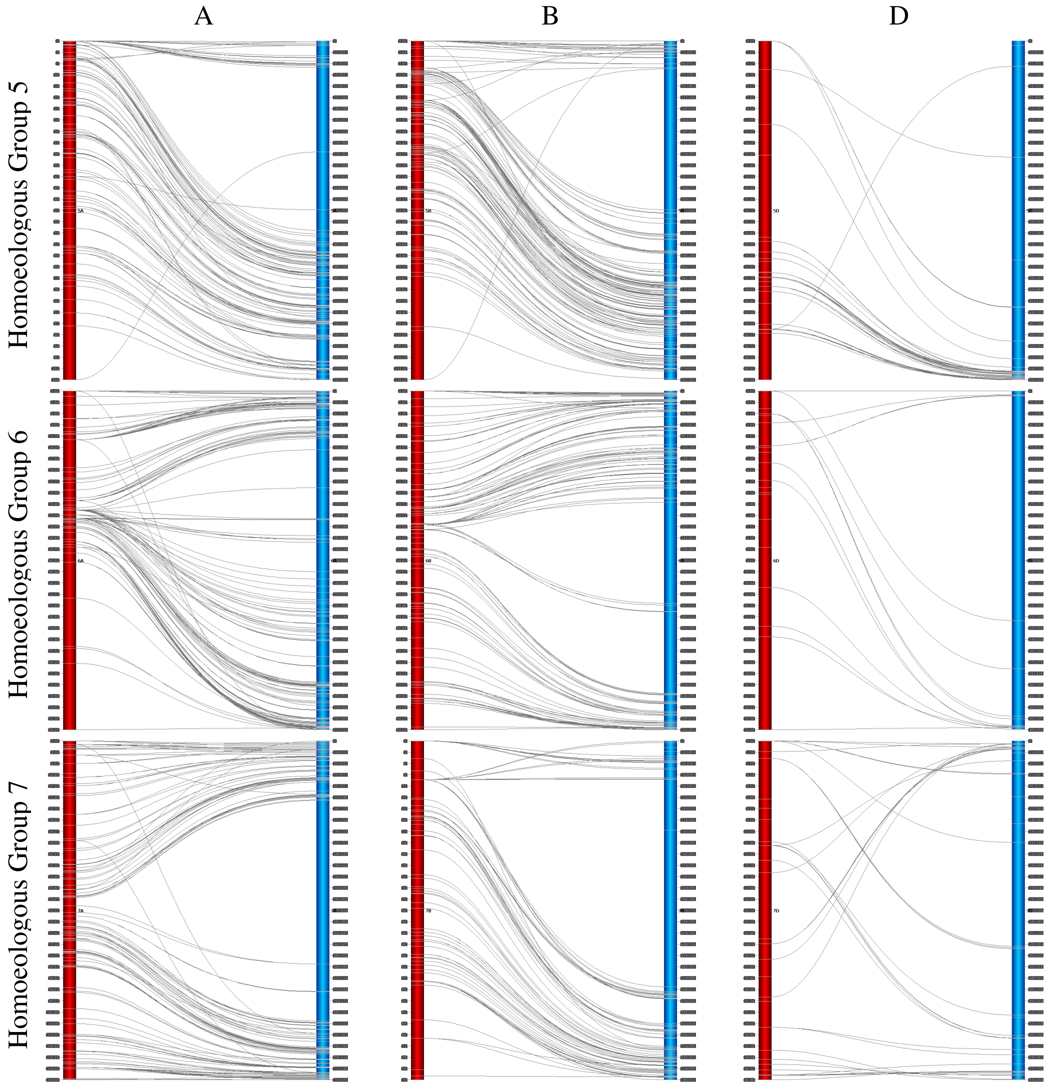
A
B
D
Homoeologous Group 5
Homoeologous Group 6
Homoeologous Group 7

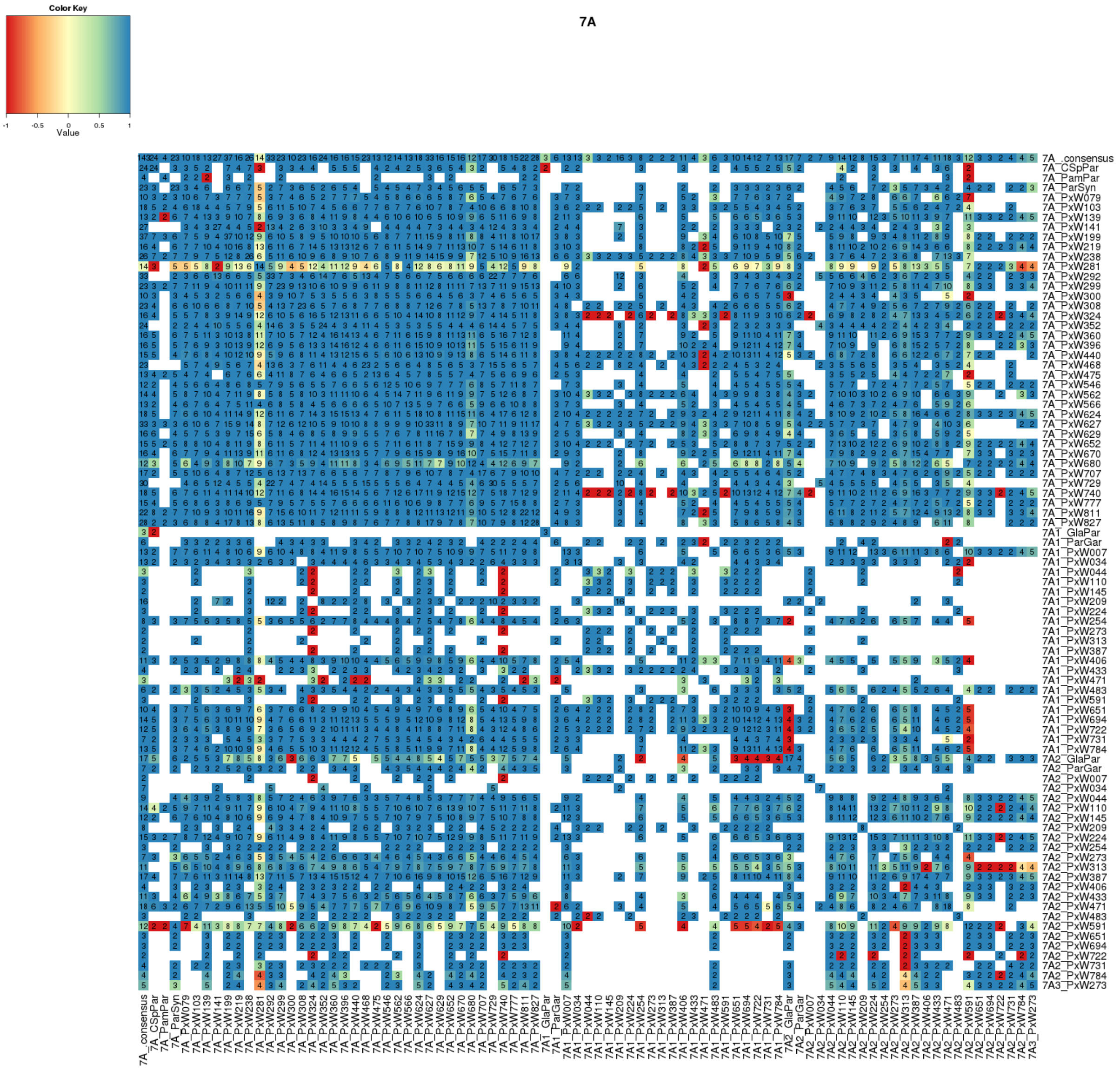

Figure S 4 Heatmap of the marker order correlation on chromosome 7A. Pair-wise comparisons of the marker order were made between all 60 bi-parental maps and also to the LRC map. Names of the maps are given at the right and below. Heatmap colors reflect the Spearman's rank correlation coefficient ρ_s in a color gradient from red (strong negative) via yellow (close to zero) to blue (strong positive). The color gradient is given on the top right. The numbers in the colored squares are the numbers of markers in common between the maps compared. Gaps in the matrix are due to not enough common markers.

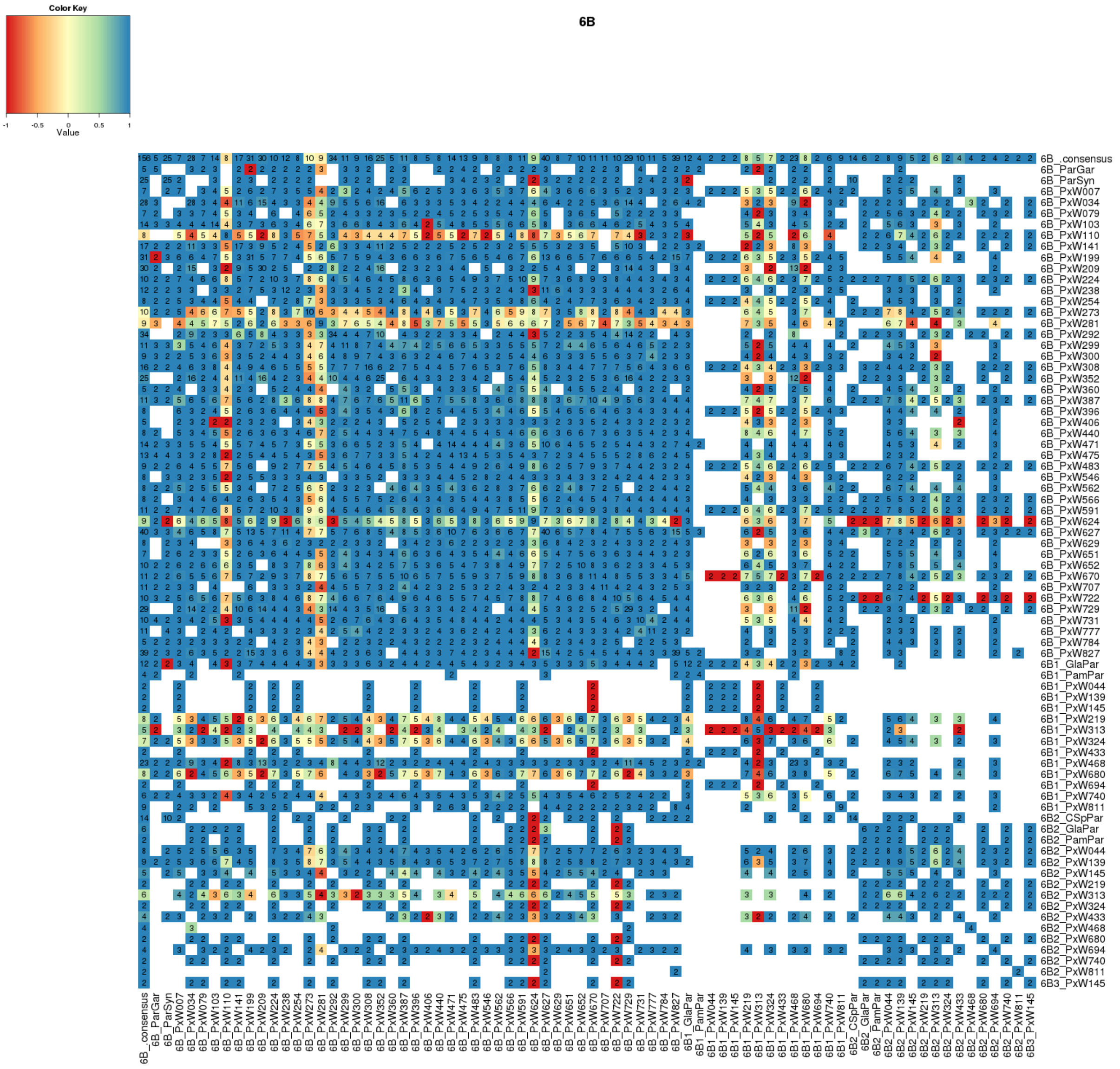

Figure S 5 Heatmap of the marker order correlation on chromosome 6B. Pair-wise comparisons of the marker order were made between all 60 bi-parental maps and also to the LRC map. Names of the maps are given at the right and below. Heatmap colors reflect the Spearman's rank correlation coefficient ρ_s in a color gradient from red (strong negative) via yellow (close to zero) to blue (strong positive). The color gradient is given on the top right. The numbers in the colored squares are the numbers of markers in common between the maps compared. Gaps in the matrix are due to not enough common markers.

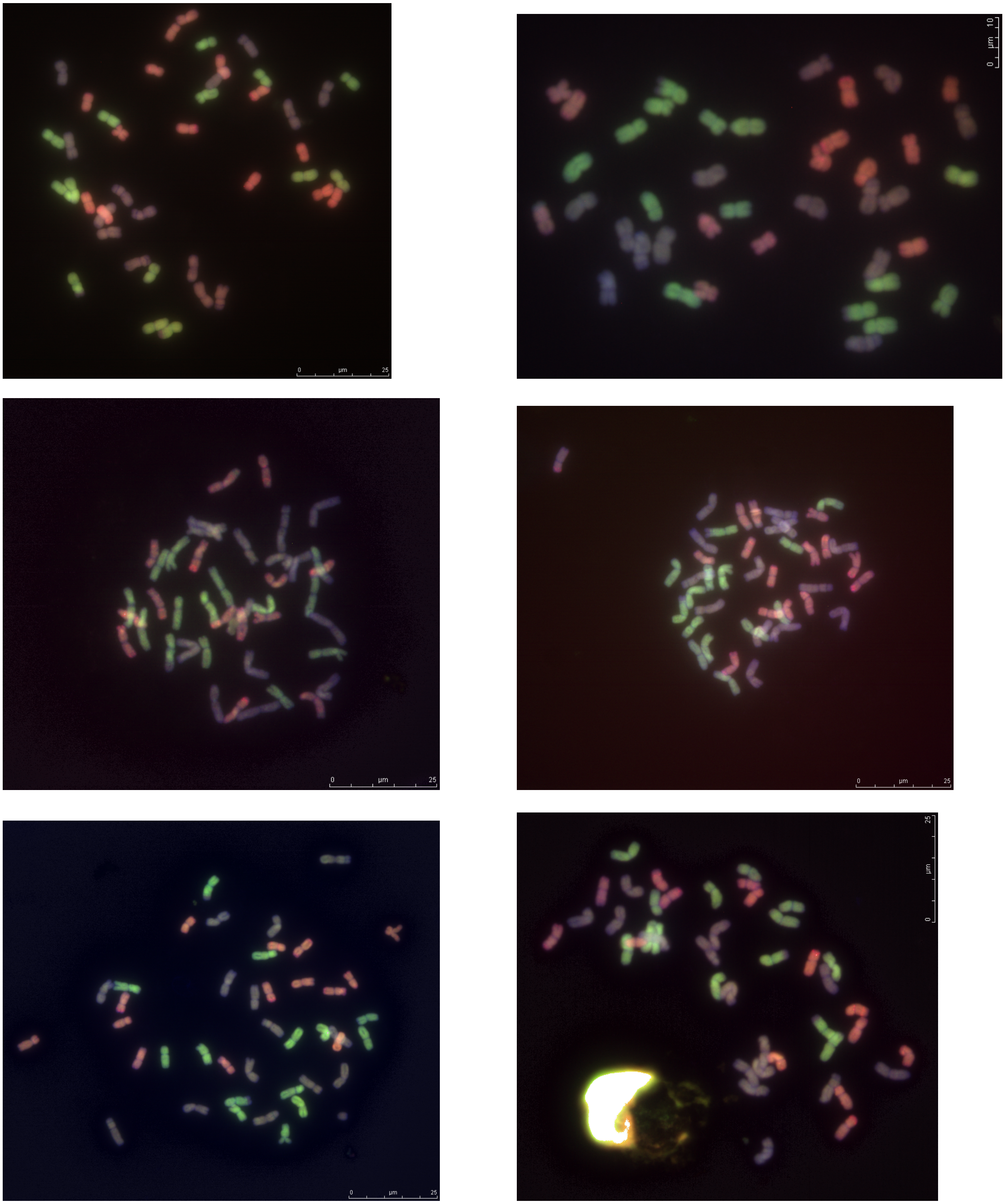

Figure S 6 Genome *in situ* hybridisation (GISH) performed with accession W103 (top-left), W468 (top-right) W139 (2x middle), W360 (2x bottom).

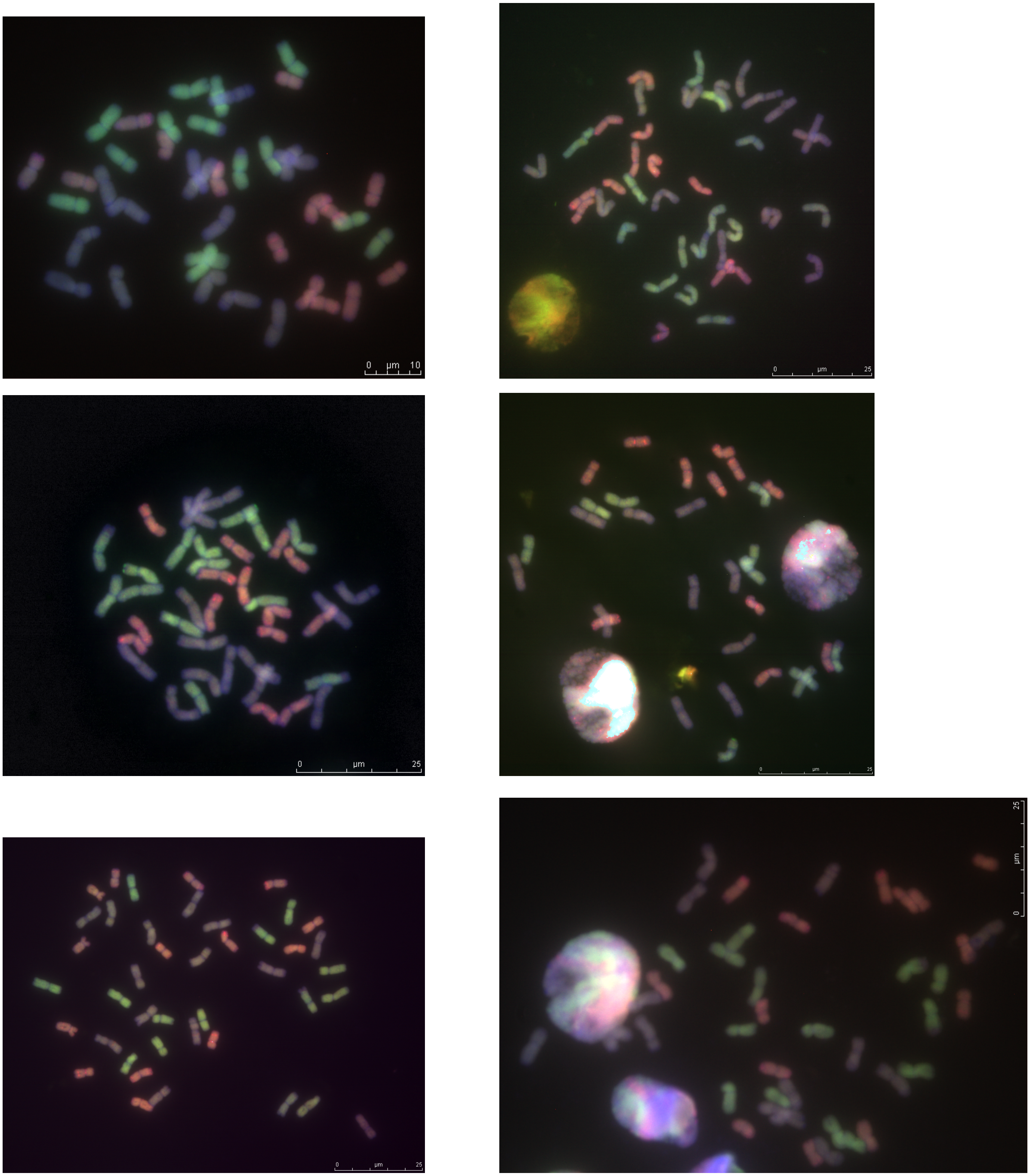

Figure S 7 Genome *in situ* hybridisation (GISH) performed with accession W546 (top-left), W624 (top-right), W680 (middle 2x), W740 (bottom-left), W827 (bottom-right).

Figure S 8 Segregation distortion aligned along the LRC map chromosomes. Chromosomes are depicted as bold black vertical bar underneath their name. Marker positions are indicated by thin black horizontal lines, crossing the chromosome. Putative centromere regions are highlighted in red on the chromosome. Population names are listed on top in alpha-numerical order starting on the left side of the chromosome bar. Each population is depicted in a separate color, color codes are given at the end of the consensus map. Segregation distortion for a marker is depicted as a short line underneath the population name, at the same height as the marker position on the LRC map. Significant levels for segregation distortion is coded as line width, the smaller the significant level, the wider the line. Segregation distorted markers are only included if the p-value was below 0.05 (adjusted for multiple testing).

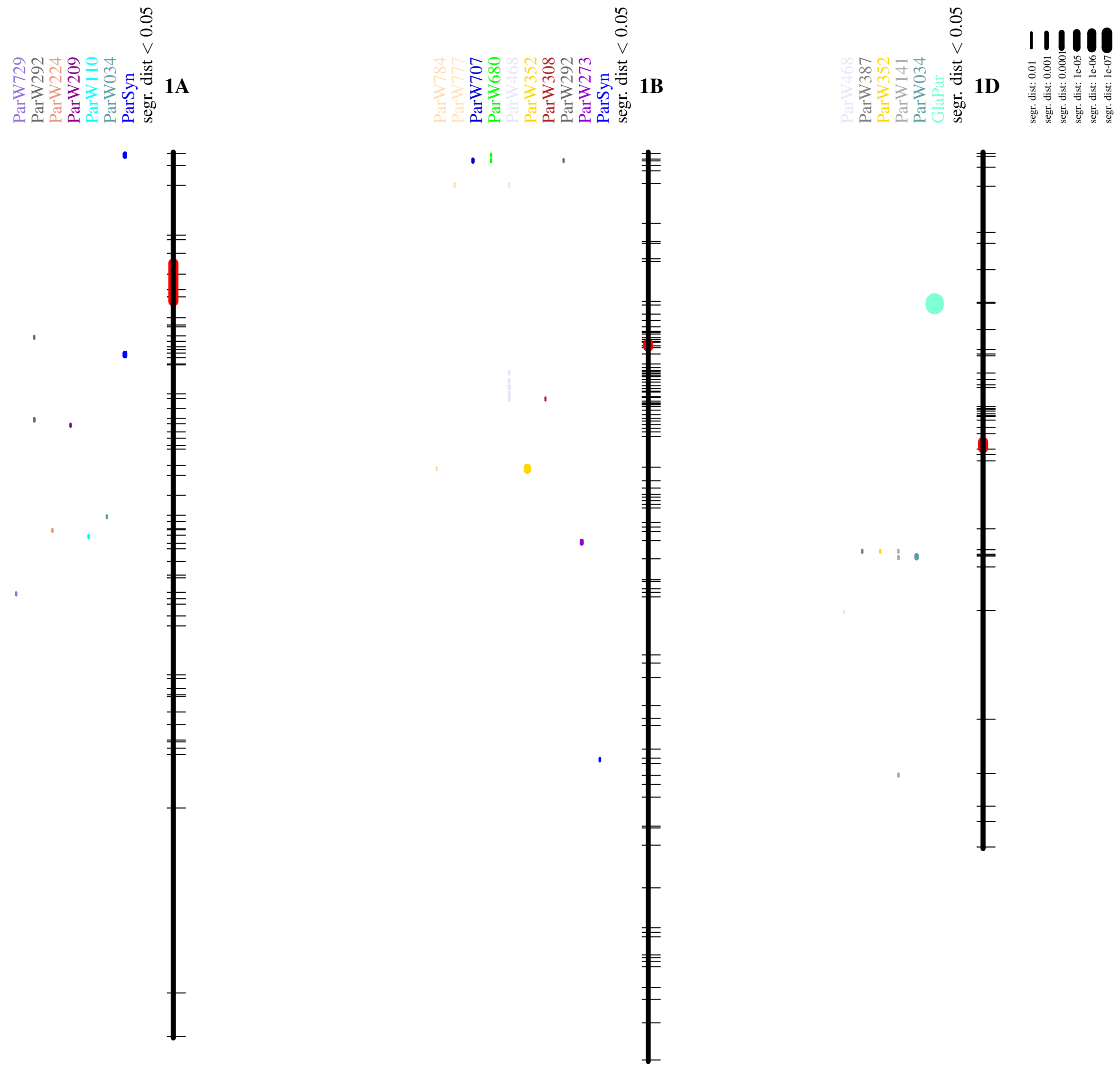

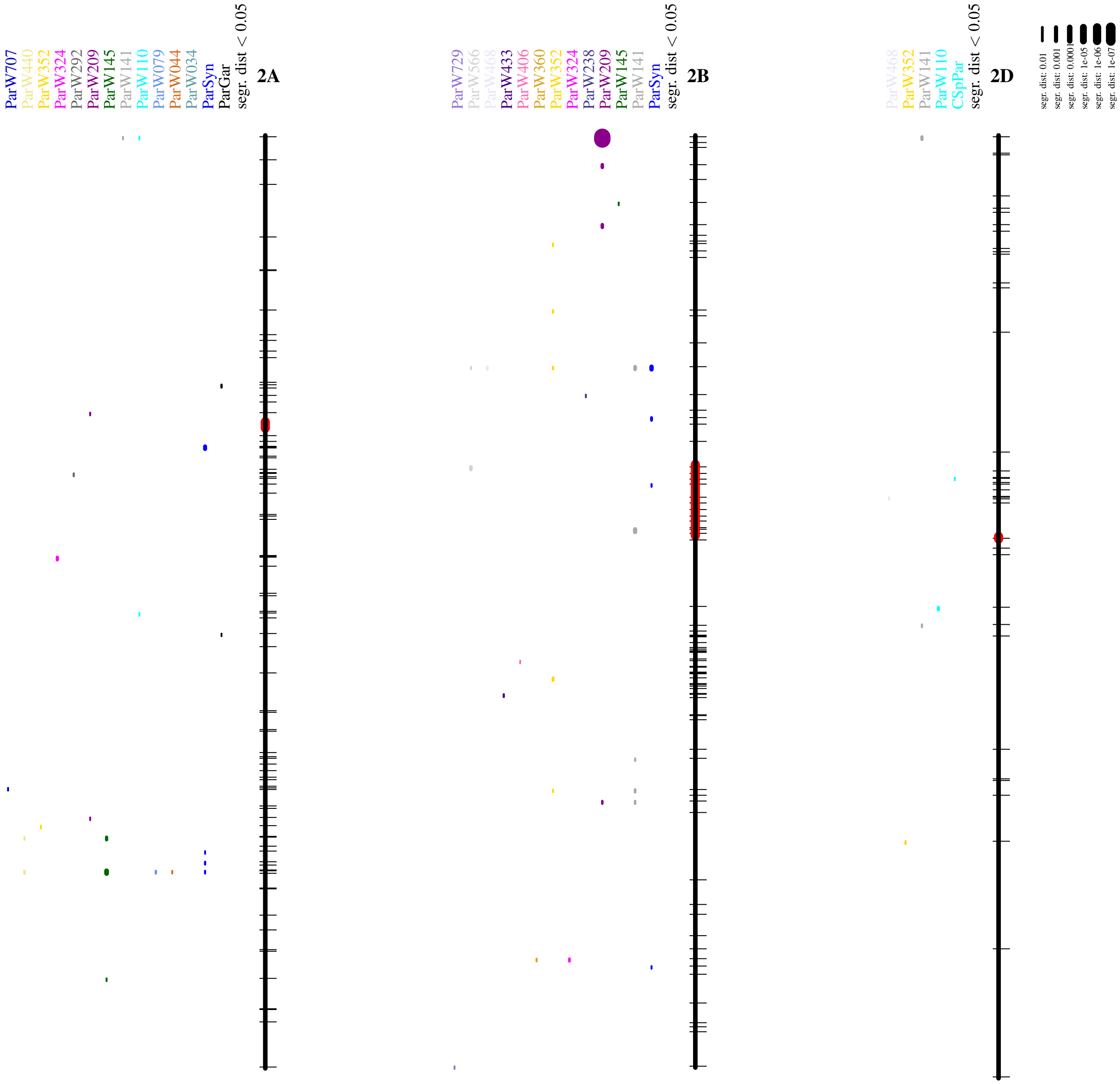
2A
ParW707
ParW440
ParW352
ParW324
ParW292
ParW209
ParW145
ParW141
ParW110
ParW079
ParW044
ParW034
ParSyn
ParGar
segr. dist < 0.05
2B
ParW729
ParW566
ParW468
ParW433
ParW406
ParW360
ParW352
ParW324
ParW238
ParW209
ParW145
ParW141
ParSyn
segr. dist < 0.05
2D
ParW468
ParW352
ParW141
ParW110
CSpPar
segr. dist < 0.05
segr. dist: 0.01
segr. dist: 0.001
segr. dist: 0.0001
segr. dist: 1e-05
segr. dist: 1e-06
segr. dist: 1e-07

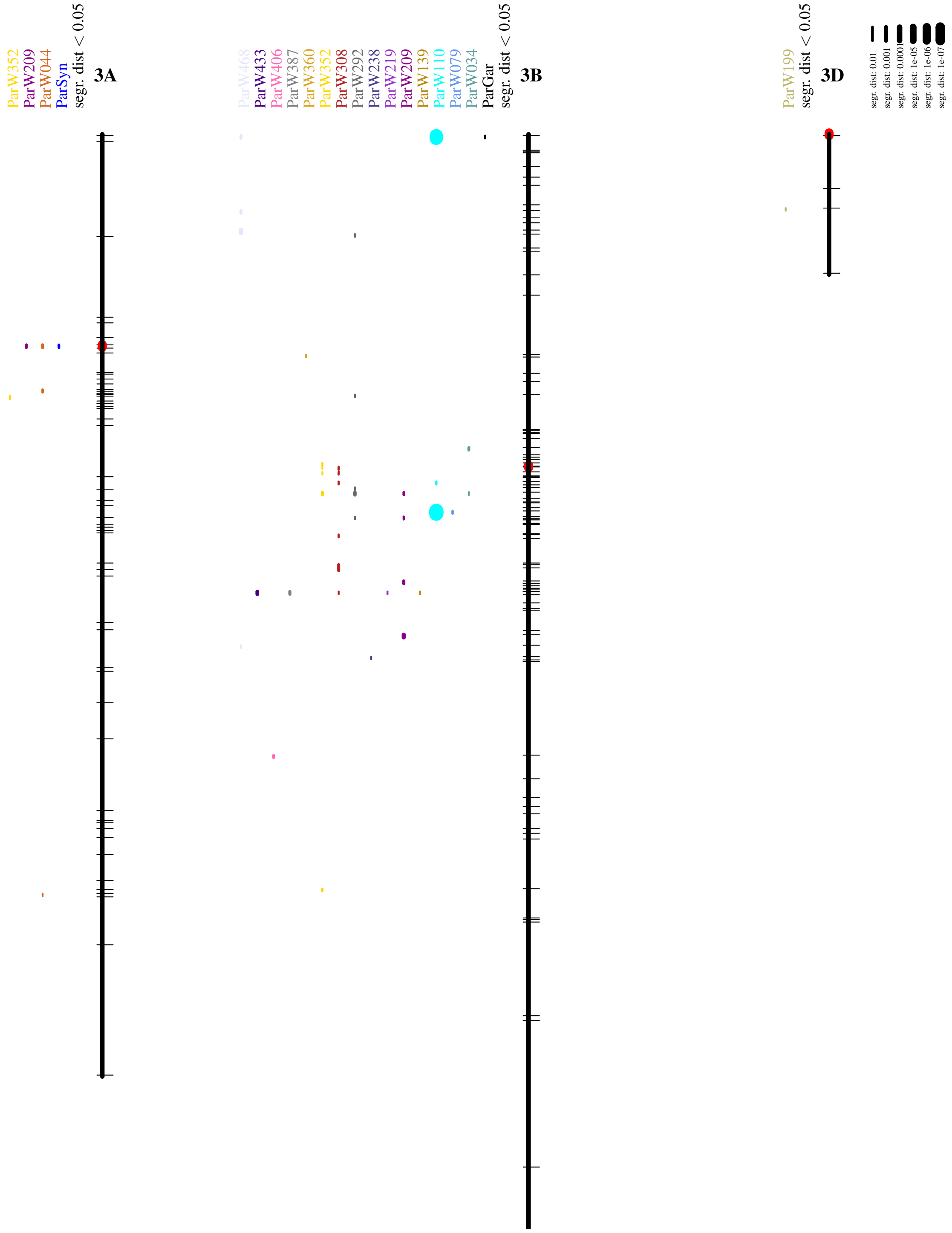

3A
segr. dist < 0.05
ParSyn
ParW044
ParW209
ParW352
3B
segr. dist < 0.05
ParGar
ParW034
ParW079
ParW110
ParW139
ParW209
ParW219
ParW238
ParW292
ParW308
ParW352
ParW360
ParW387
ParW406
ParW433
ParW468
3D
segr. dist < 0.05
ParW199
segr. dist: 0.01
segr. dist: 0.001
segr. dist: 1e-05
segr. dist: 1e-06
segr. dist: 1e-07

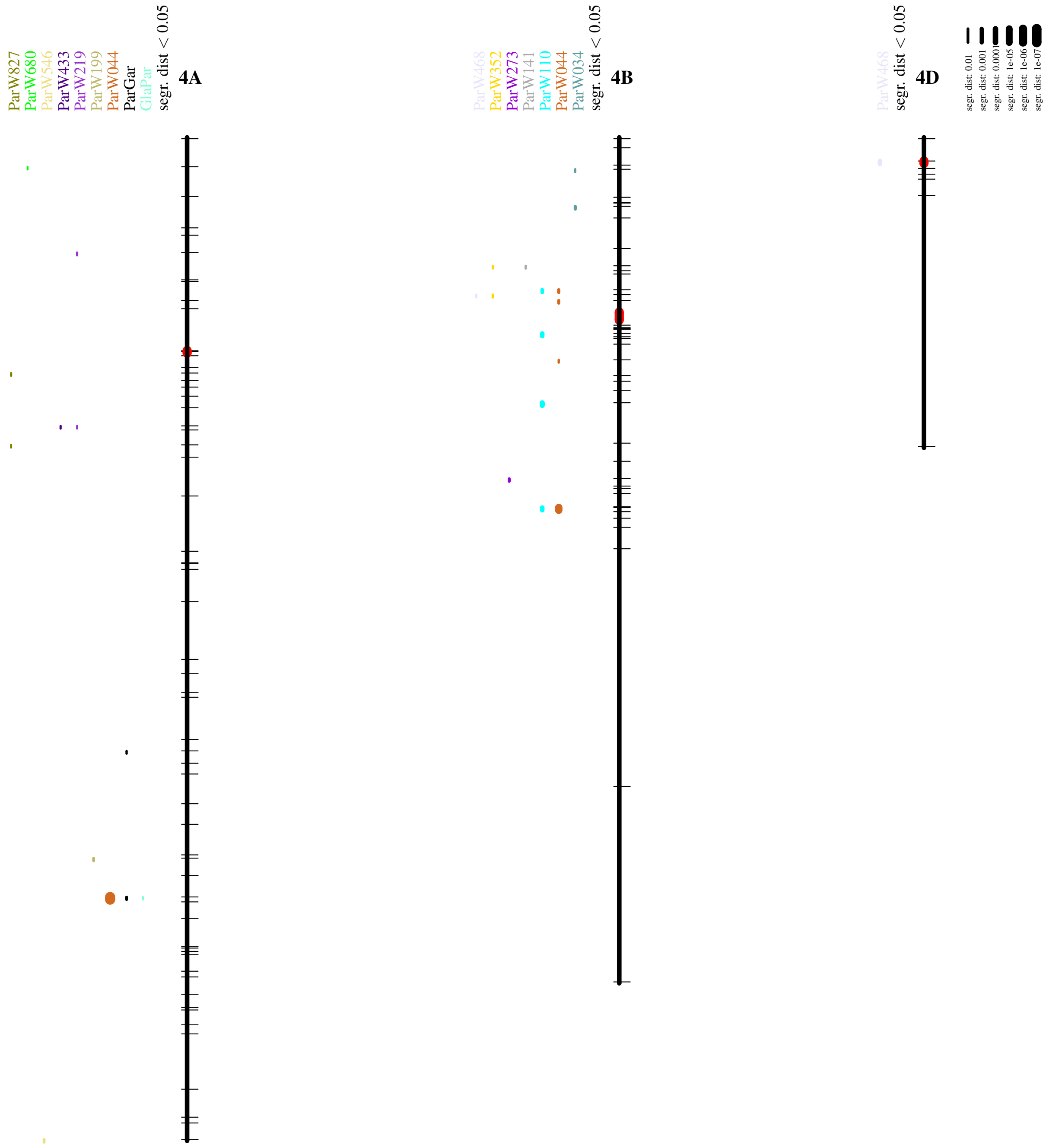
4A
ParW827
ParW680
ParW546
ParW433
ParW219
ParW199
ParW044
ParGar
GlaPar
segr. dist < 0.05
4B
ParW468
ParW352
ParW273
ParW141
ParW110
ParW044
ParW034
segr. dist < 0.05
4D
ParW468
segr. dist < 0.05
segr. dist: 0.01
segr. dist: 0.001
segr. dist: 0.0001
segr. dist: 1e-05
segr. dist: 1e-06
segr. dist: 1e-07

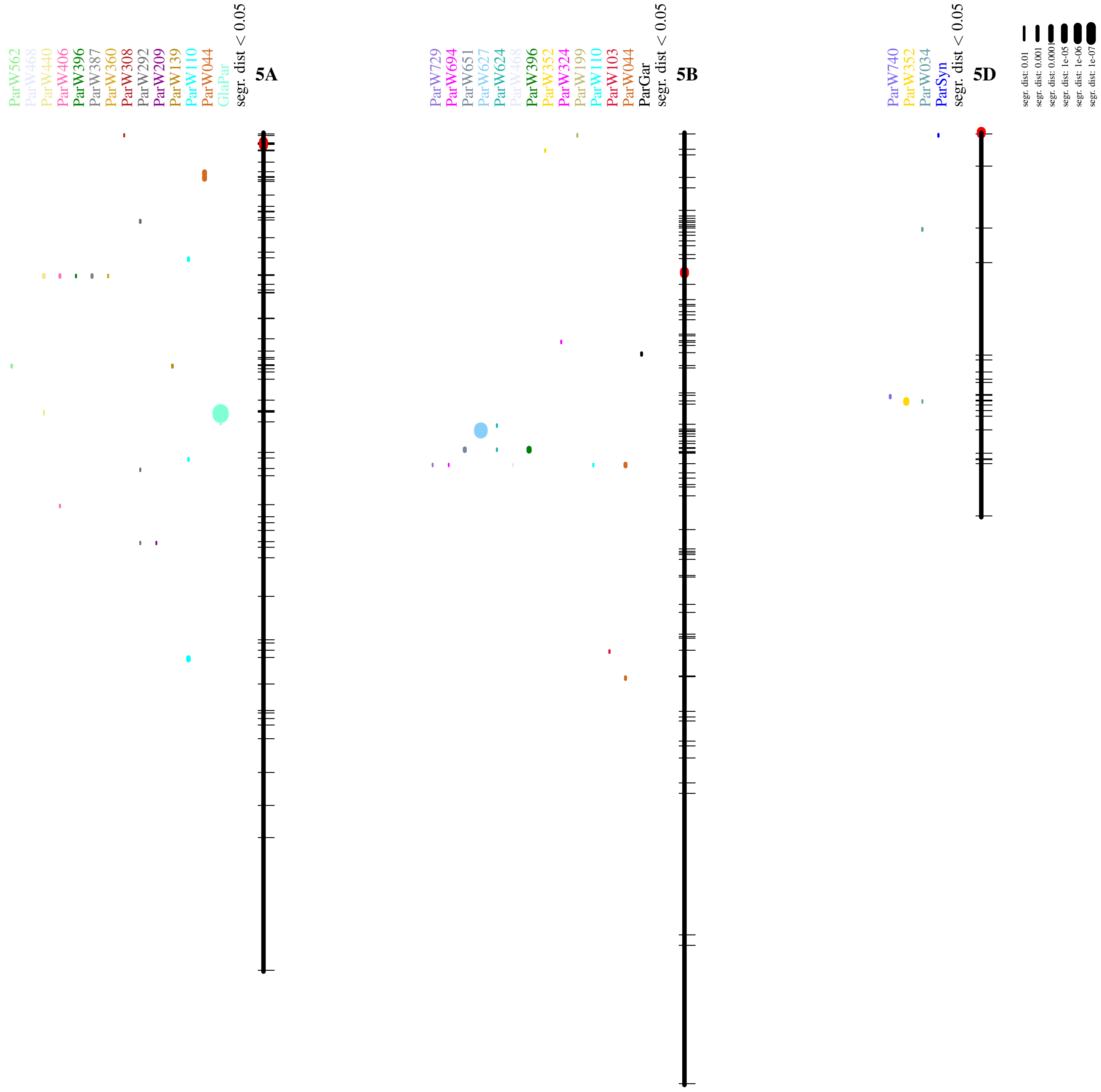
5A
segr. dist < 0.05
GlaPar
ParW044
ParW110
ParW139
ParW209
ParW292
ParW308
ParW360
ParW387
ParW396
ParW406
ParW440
ParW468
ParW562
5B
segr. dist < 0.05
ParGar
ParW044
ParW103
ParW110
ParW199
ParW324
ParW352
ParW396
ParW468
ParW624
ParW627
ParW651
ParW694
ParW729
5D
segr. dist < 0.05
ParSyn
ParW034
ParW352
ParW740
segr. dist: 0.01
segr. dist: 0.001
segr. dist: 0.0001
segr. dist: 1e-05
segr. dist: 1e-06
segr. dist: 1e-07

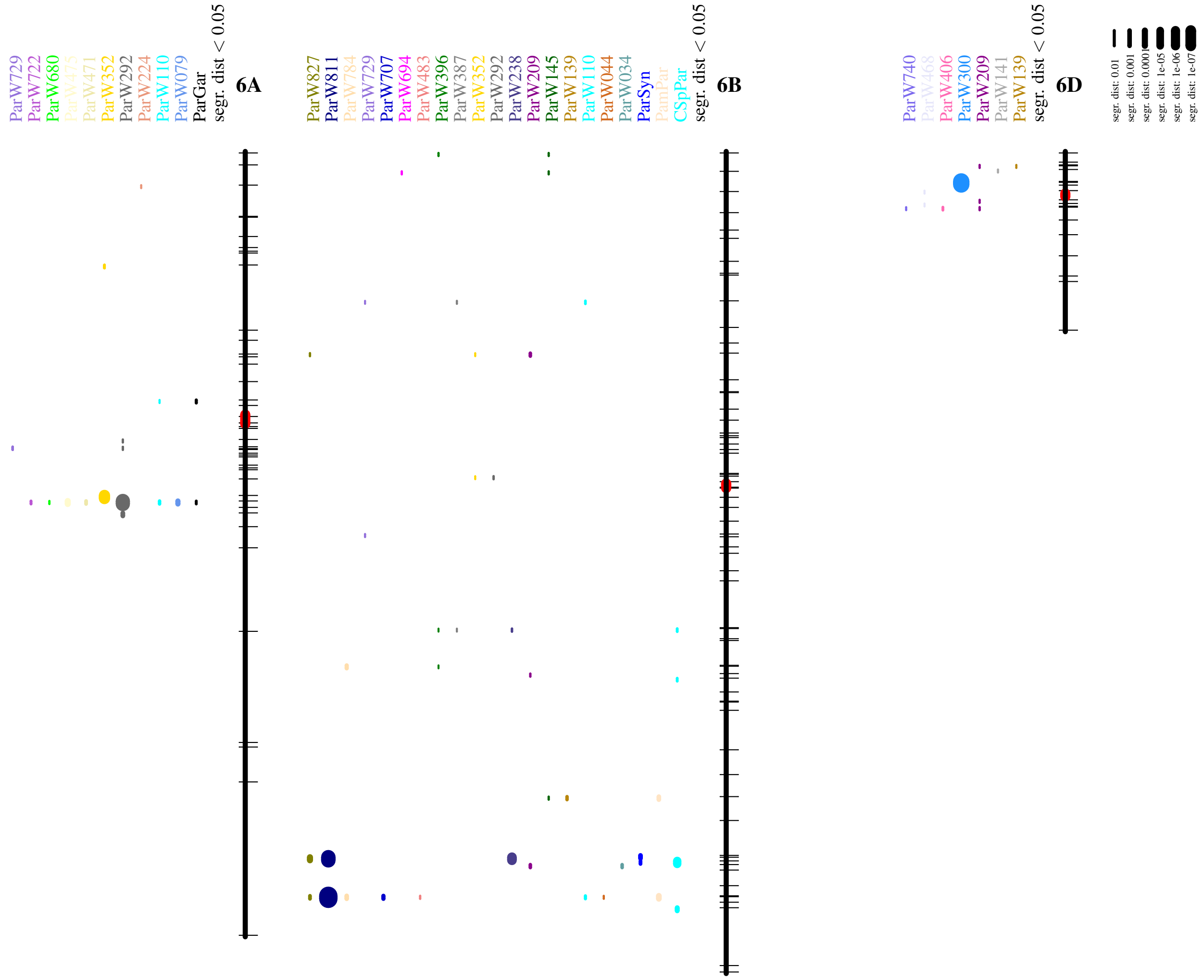
6A
ParW729
ParW722
ParW680
ParW471
ParW352
ParW292
ParW224
ParW110
ParW079
ParGar
segr. dist < 0.05
6B
ParW827
ParW811
ParW784
ParW729
ParW707
ParW694
ParW483
ParW396
ParW387
ParW352
ParW292
ParW238
ParW209
ParW145
ParW139
ParW110
ParW044
ParW034
ParSyn
CSpPar
segr. dist < 0.05
6D
ParW740
ParW406
ParW300
ParW209
ParW141
ParW139
segr. dist < 0.05
segr. dist: 0.01
segr. dist: 0.001
segr. dist: 1e-05
segr. dist: 1e-06
segr. dist: 1e-07

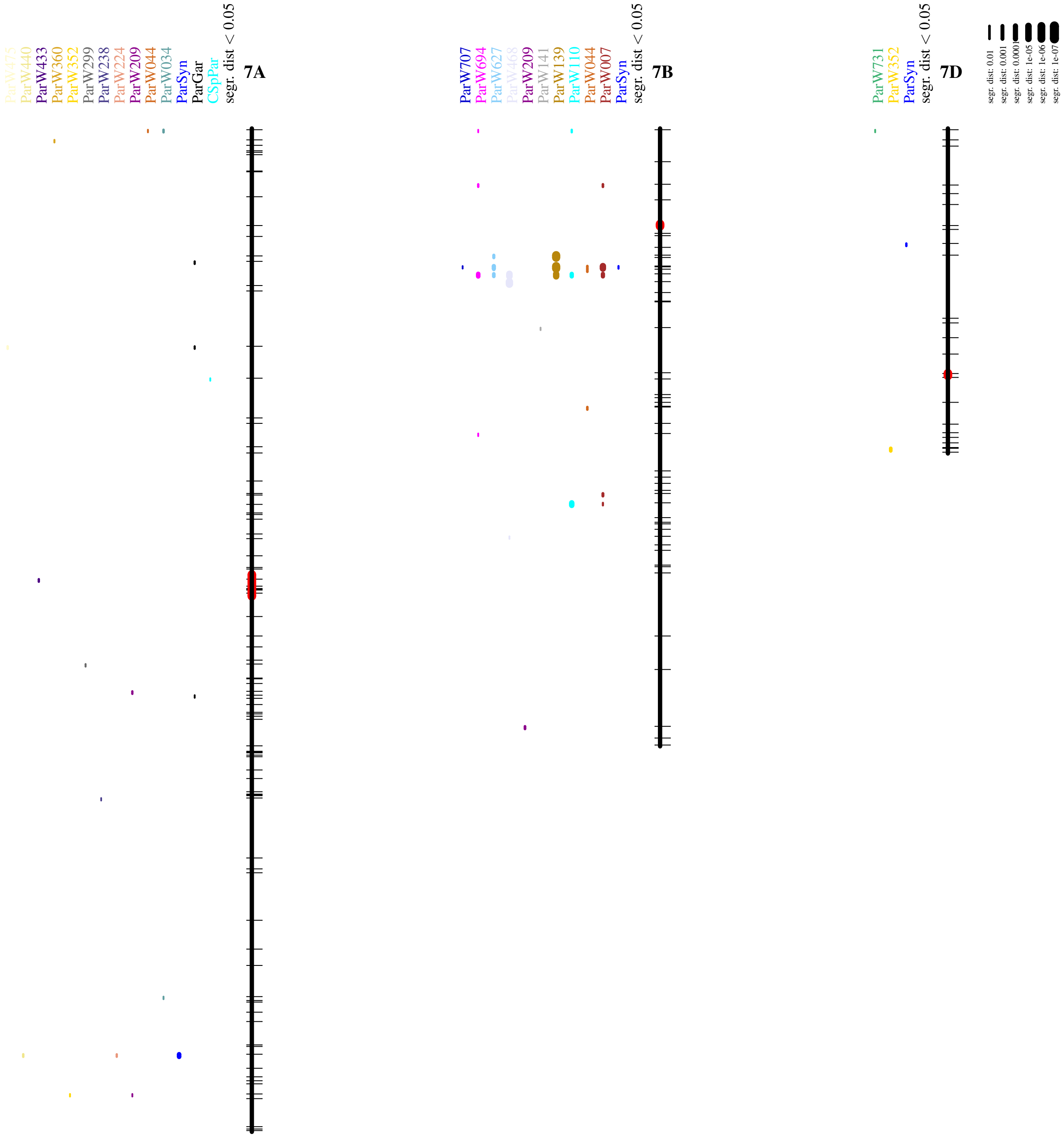
7A
ParW475
ParW440
ParW433
ParW360
ParW352
ParW299
ParW238
ParW224
ParW209
ParW044
ParW034
ParSyn
ParGar
CSpPar
segr. dist < 0.05
7B
ParW707
ParW694
ParW627
ParW468
ParW209
ParW141
ParW139
ParW110
ParW044
ParW007
ParSyn
segr. dist < 0.05
7D
ParW731
ParW352
ParSyn
segr. dist < 0.05
segr. dist: 0.01
segr. dist: 0.001
segr. dist: 0.0001
segr. dist: 1e-05
segr. dist: 1e-06
segr. dist: 1e-07

- CSpPar: Aqua
- GlaPar: Aquamarine
- PamPar: Bisque
- ParGar: Black
- ParSyn: Blue
- ParW007: Brown
- ParW034: CadetBlue
- ParW044: Chocolate
- ParW079: CornflowerBlue
- ParW103: Crimson
- ParW110: Cyan
- ParW139: DarkGoldenrod
- ParW141: DarkGray
- ParW145: DarkGreen
- ParW199: DarkKhaki
- ParW209: DarkMagenta
- ParW219: DarkOrchid
- ParW224: DarkSalmon
- ParW238: DarkSlateBlue
- ParW254: DarkTurquoise
- ParW273: DarkViolet
- ParW281: DeepPink
- ParW292: DimGray
- ParW299: DimGrey
- ParW300: DodgerBlue
- ParW308: FireBrick
- ParW313: ForestGreen
- ParW324: Fuchsia
- ParW352: Gold
- ParW360: Goldenrod
- ParW387: Gray
- ParW396: Green
- ParW406: HotPink
- ParW433: Indigo
- ParW440: Khaki
- ParW468: Lavender
- ParW471: PaleGoldenrod
- ParW475: LemonChiffon
- ParW483: LightCoral
- ParW546: LightGoldenrod
- ParW562: LightGreen
- ParW566: LightGrey
- ParW591: LightSalmon
- ParW624: LightSeaGreen
- ParW627: LightSkyBlue
- ParW629: LightSlateBlue
- ParW651: LightSlateGray
- ParW652: LightSlateGrey
- ParW670: LightSteelBlue
- ParW680: Lime
- ParW694: Magenta
- ParW707: MediumBlue
- ParW722: MediumOrchid
- ParW729: MediumPurple
- ParW731: MediumSeaGreen
- ParW740: MediumSlateBlue
- ParW777: Moccasin
- ParW784: NavajoWhite
- ParW811: Navy
- ParW827: Olive
- pop: BlueViolet

Figure S 9 Translocation of markers of different populations aligned along the LRC map. Chromosomes are depicted as bold black vertical bar underneath their name. Marker positions are indicated by thin black horizontal lines, crossing the chromosome. Putative centromere regions are highlighted in red on the chromosome. Names of populations with translocation are listed on top in alpha-numerical order starting on the left side of the chromosome bar. Each population is depicted in a separate color, color codes are given at the end of the consensus map. Translocations are depicted as vertical colored dots underneath the population name, at the height of the marker positions on the consensus chromosome. LOD scores levels are coded as line widths, the larger the score, the wider the line. Markers linked to a translocated marker are connected by thin vertical lines. Translocations are only shown if the linkage LOD score was above 10.

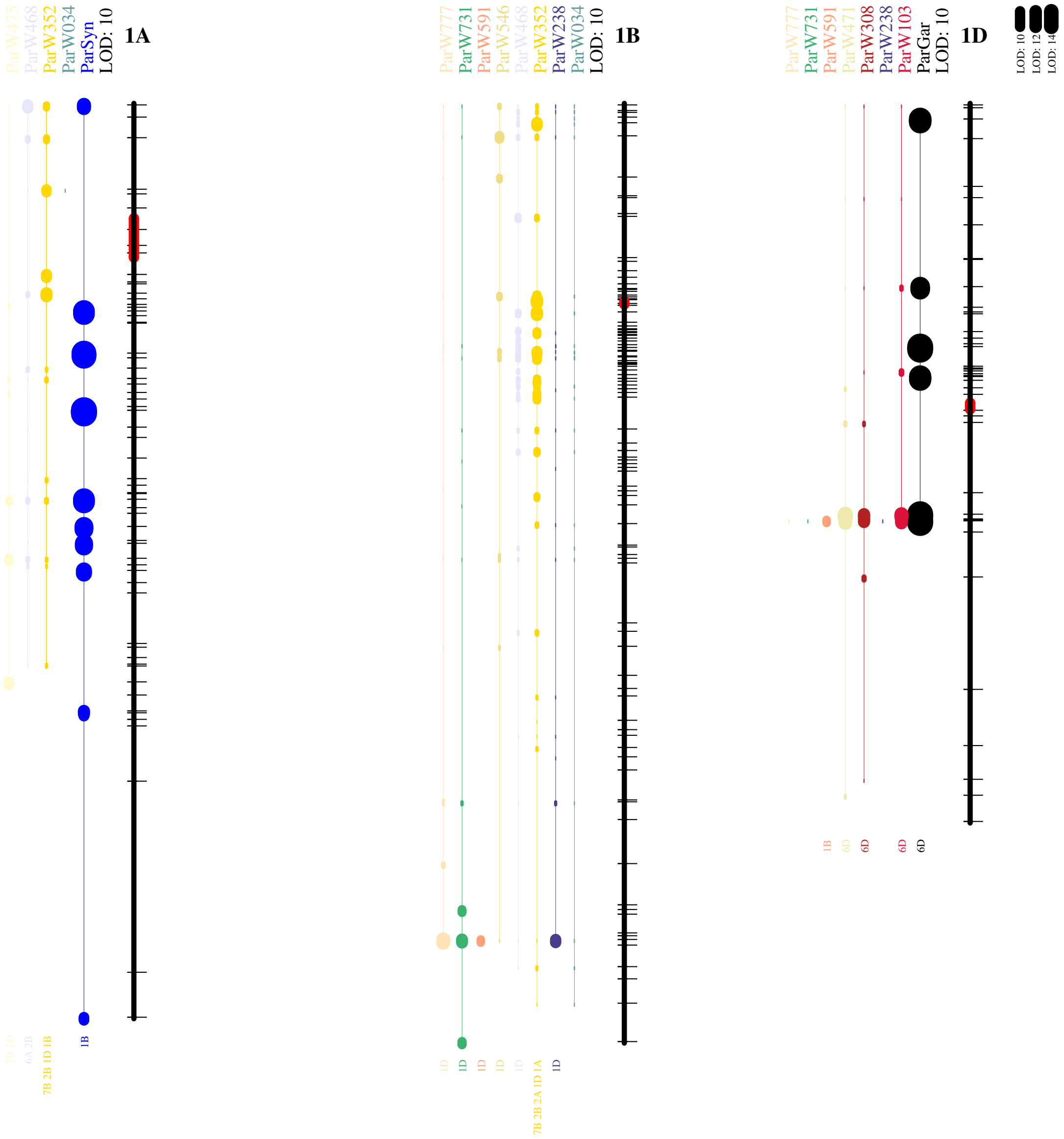

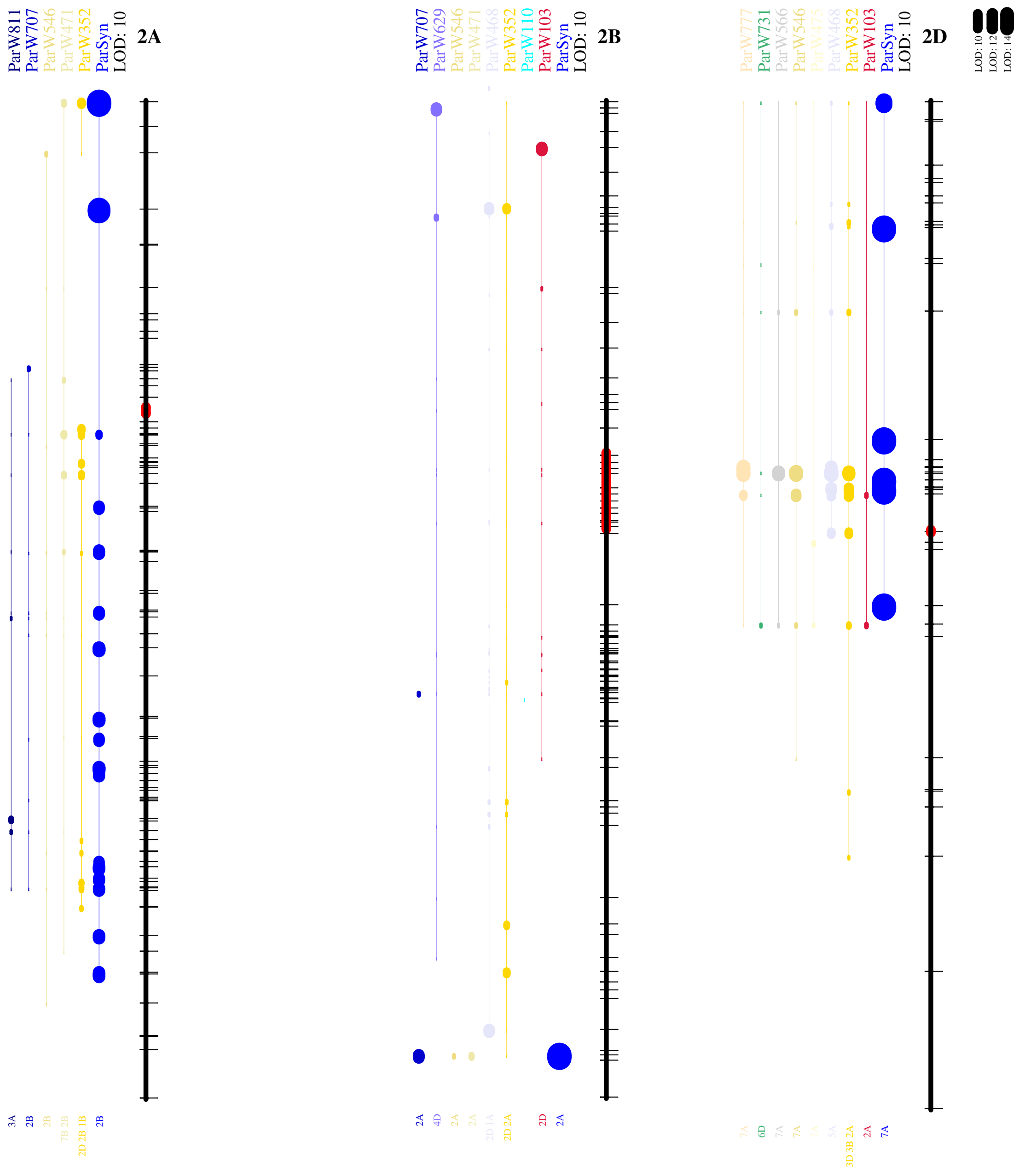

2A
ParW811
ParW707
ParW546
ParW471
ParW352
ParSyn
LOD: 10
2B
ParW707
ParW629
ParW546
ParW471
ParW468
ParW352
ParW110
ParW103
ParSyn
LOD: 10
2D
ParW777
ParW731
ParW566
ParW546
ParW475
ParW468
ParW352
ParW103
ParSyn
LOD: 10
LOD: 10
LOD: 12
LOD: 14

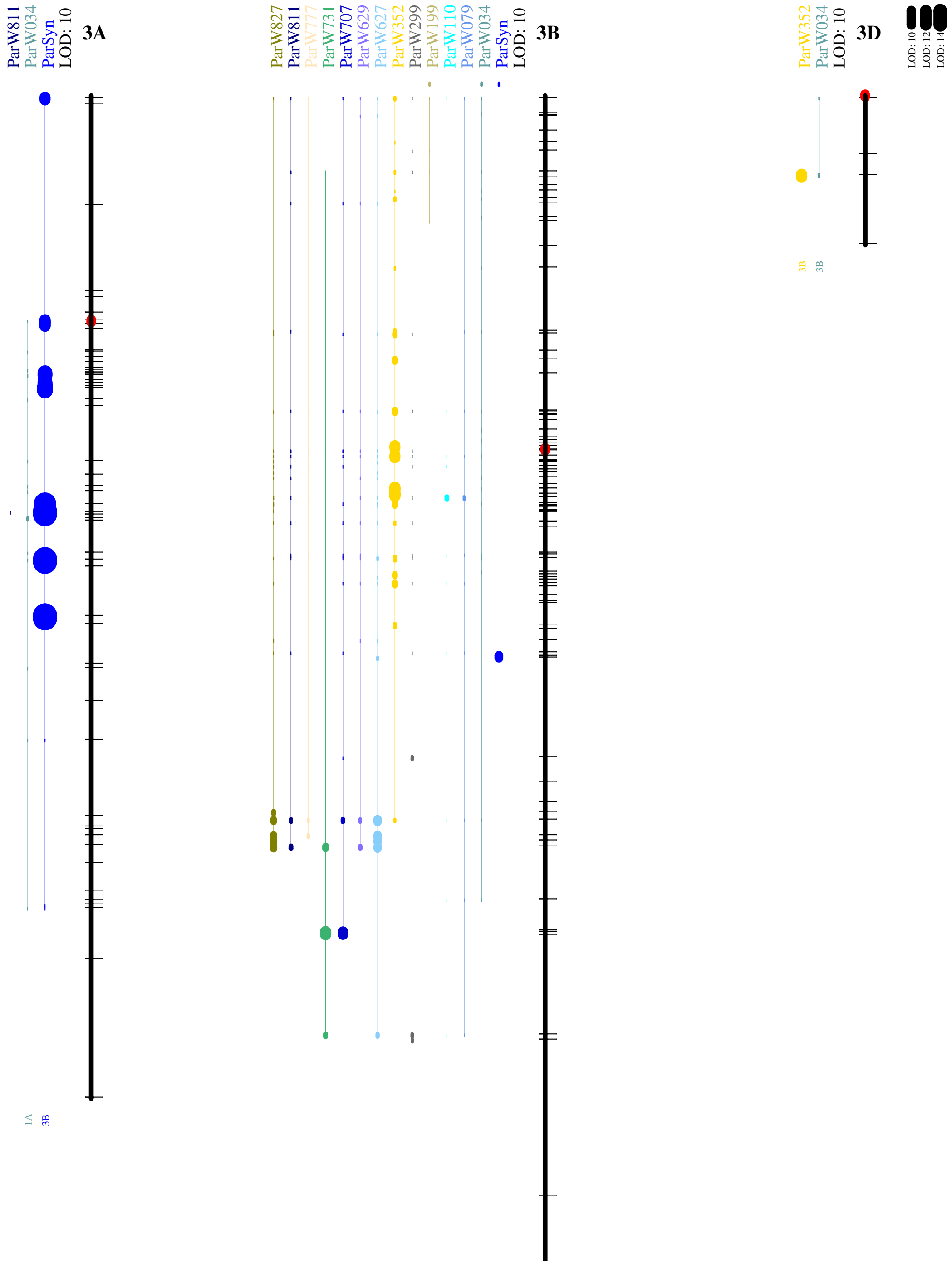
3A
LOD: 10
ParSyn
ParW034
ParW811
1A
3B
3B
LOD: 10
ParSyn
ParW034
ParW079
ParW110
ParW199
ParW299
ParW352
ParW627
ParW629
ParW707
ParW731
ParW777
ParW811
ParW827
3D
LOD: 10
ParW034
ParW352
3B
3B
LOD: 10
LOD: 12
LOD: 14

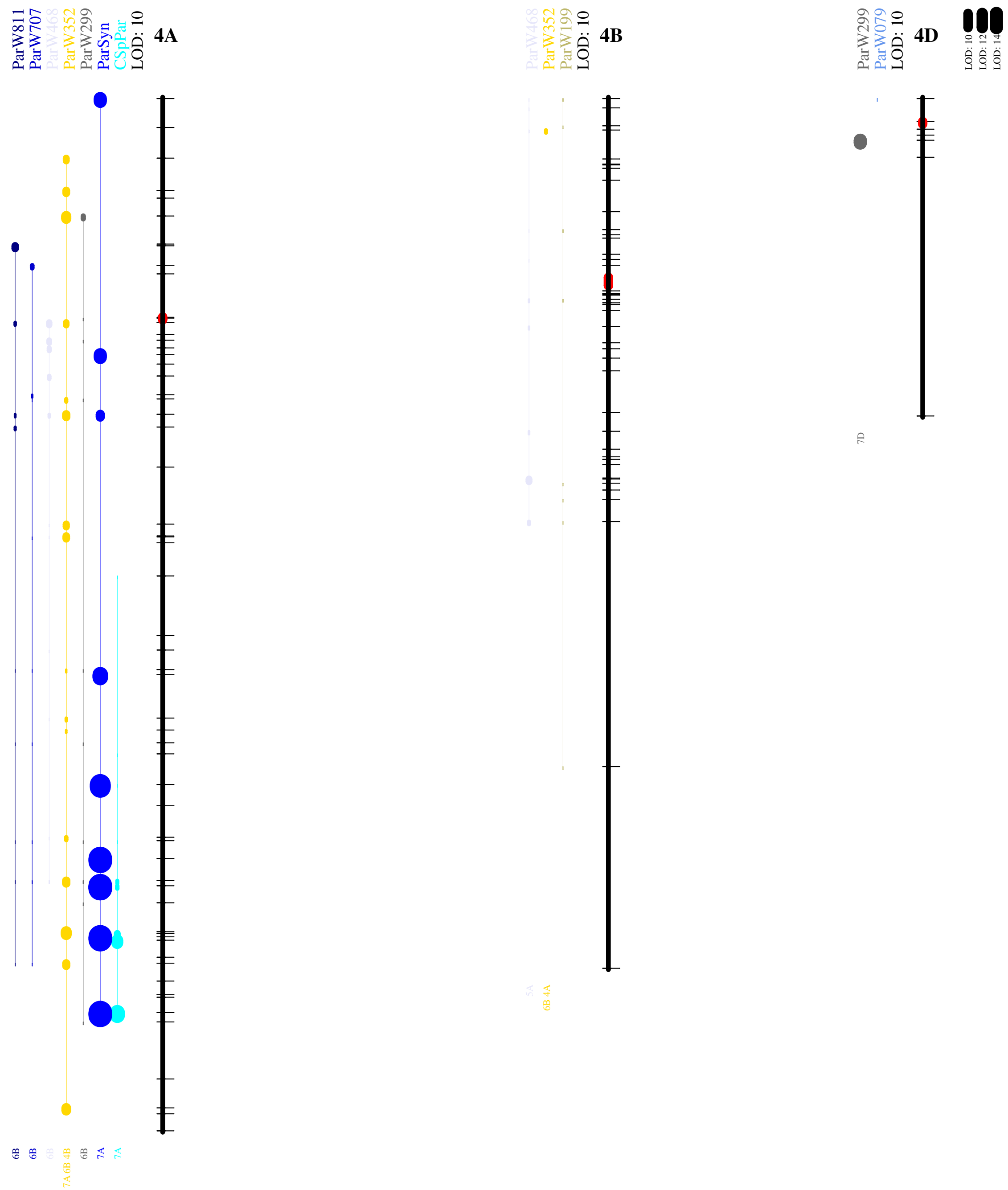
4A
LOD: 10
CSpPar
ParSyn
ParW299
ParW352
ParW468
ParW707
ParW811
4B
LOD: 10
ParW199
ParW352
ParW468
4D
LOD: 10
ParW079
ParW299
LOD: 10
LOD: 12
LOD: 14

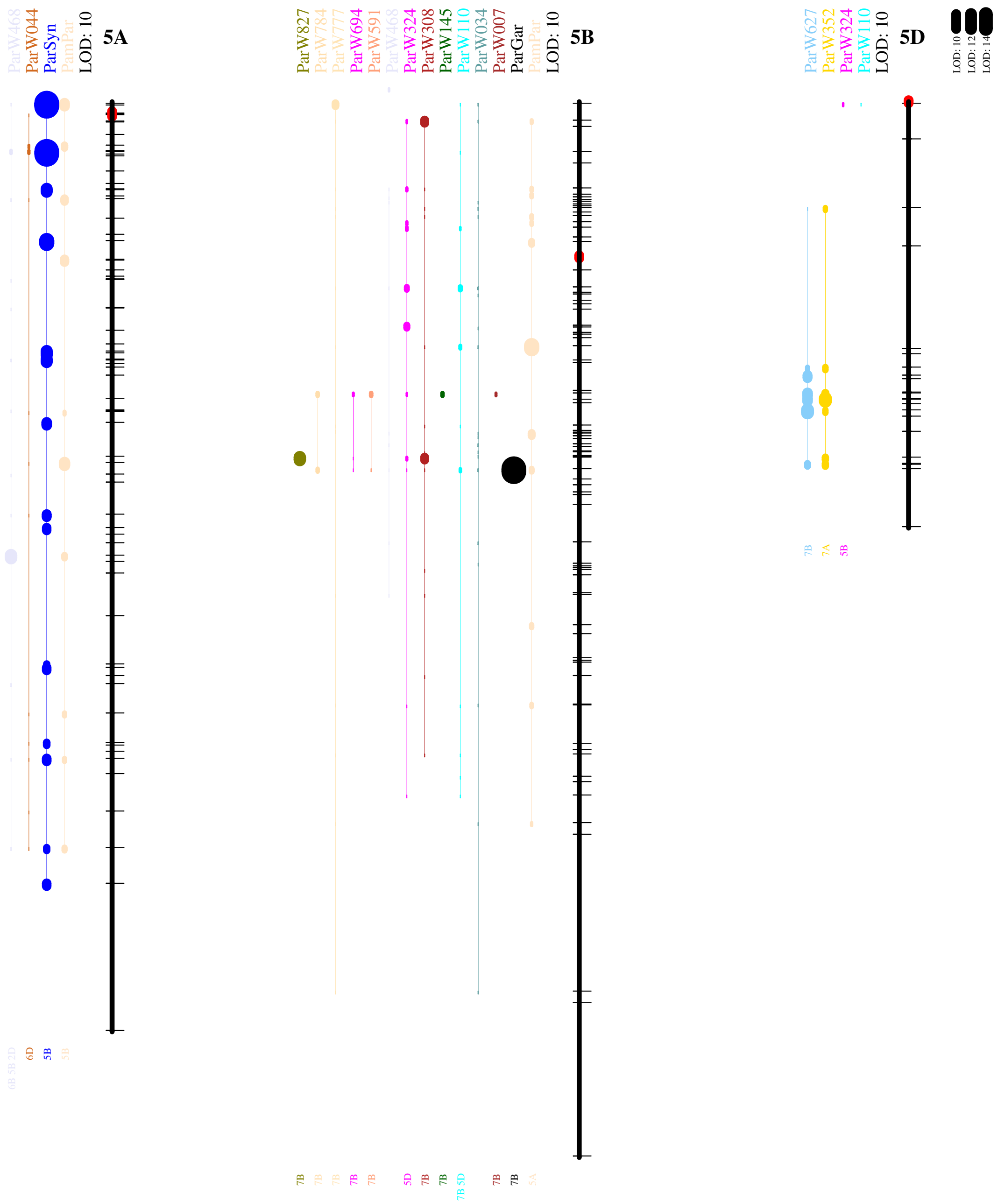
5A
LOD: 10
ParSyn
ParW044
ParW468
PamPar
5B
ParGar
ParW007
ParW034
ParW110
ParW145
ParW308
ParW324
ParW468
ParW591
ParW694
ParW777
ParW784
ParW827
5D
ParW627
ParW352
LOD: 12
LOD: 14

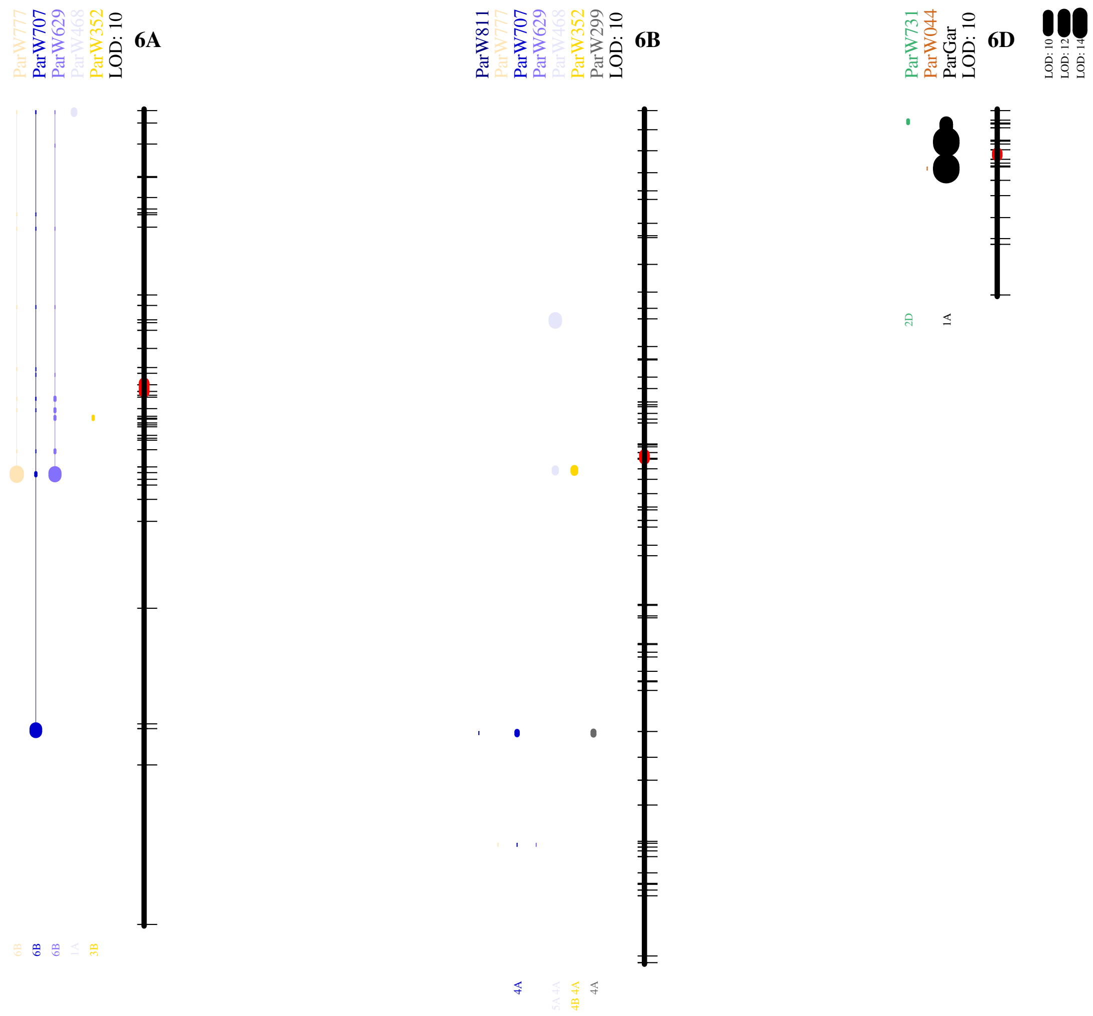
6A
LOD: 10
ParW352
ParW468
ParW629
ParW707
ParW777
6B
6B
6B
1A
3B
6B
LOD: 10
ParW299
ParW352
ParW468
ParW629
ParW707
ParW777
ParW811
4A
5A 4A
4B 4A
4A
6D
LOD: 10
ParGar
ParW044
ParW731
2D
1A
LOD: 10
LOD: 12
LOD: 14

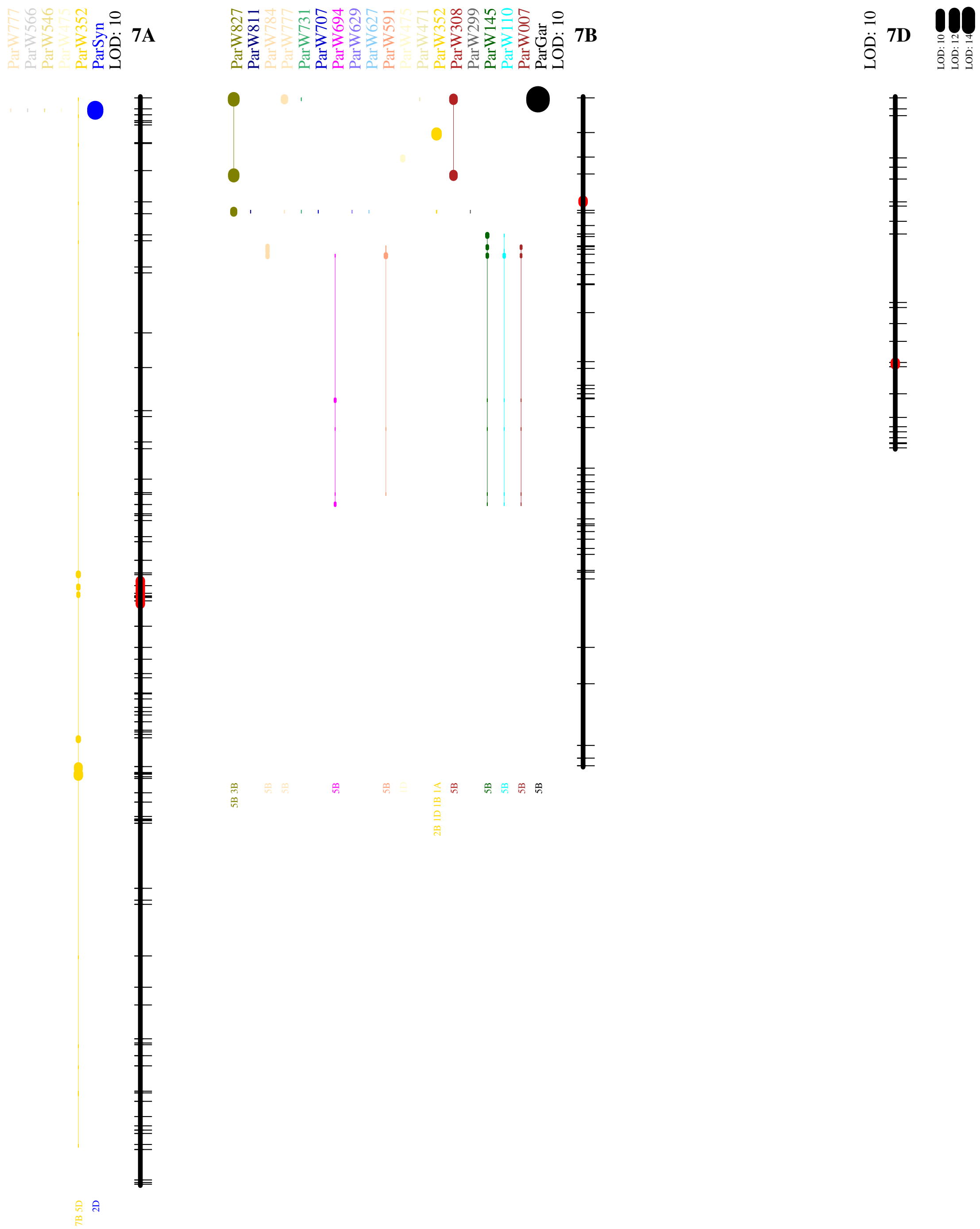
7A
LOD: 10
ParSyn
ParW352
ParW475
ParW546
ParW566
ParW777
7B 5D
2D
7B
LOD: 10
ParGar
ParW007
ParW110
ParW145
ParW299
ParW308
ParW352
ParW471
ParW475
ParW591
ParW627
ParW629
ParW694
ParW707
ParW731
ParW777
ParW784
ParW811
ParW827
5B 3B
2B 1D 1B 1A
7D
LOD: 10
LOD: 12
LOD: 14

- CSpPar: Aqua
- GlaPar: Aquamarine
- PamPar: Bisque
- ParGar: Black
- ParSyn: Blue
- ParW007: Brown
- ParW034: CadetBlue
- ParW044: Chocolate
- ParW079: CornflowerBlue
- ParW103: Crimson
- ParW110: Cyan
- ParW139: DarkGoldenrod
- ParW141: DarkGray
- ParW145: DarkGreen
- ParW199: DarkKhaki
- ParW209: DarkMagenta
- ParW219: DarkOrchid
- ParW224: DarkSalmon
- ParW238: DarkSlateBlue
- ParW254: DarkTurquoise
- ParW273: DarkViolet
- ParW281: DeepPink
- ParW292: DimGray
- ParW299: DimGrey
- ParW300: DodgerBlue
- ParW308: FireBrick
- ParW313: ForestGreen
- ParW324: Fuchsia
- ParW352: Gold
- ParW360: Goldenrod
- ParW387: Gray
- ParW396: Green
- ParW406: HotPink
- ParW433: Indigo
- ParW440: Khaki
- ParW468: Lavender
- ParW471: PaleGoldenrod
- ParW475: LemonChiffon
- ParW483: LightCoral
- ParW546: LightGoldenrod
- ParW562: LightGreen
- ParW566: LightGrey
- ParW591: LightSalmon
- ParW624: LightSeaGreen
- ParW627: LightSkyBlue
- ParW629: LightSlateBlue
- ParW651: LightSlateGray
- ParW652: LightSlateGrey
- ParW670: LightSteelBlue
- ParW680: Lime
- ParW694: Magenta
- ParW707: MediumBlue
- ParW722: MediumOrchid
- ParW729: MediumPurple
- ParW731: MediumSeaGreen
- ParW740: MediumSlateBlue
- ParW777: Moccasin
- ParW784: NavajoWhite
- ParW811: Navy
- ParW827: Olive

Figure S 10 Crossover QTL aligned along the LRC map chromosomes. Chromosomes are depicted as bold black vertical bar underneath their name. Marker positions are indicated by thin black horizontal lines, crossing the chromosome. Putative centromere regions are highlighted in red on the chromosome. Names of populations with QTL equal or above a LOD score of 2.0 on that chromosome are listed on top in alpha-numerical order starting on the left side of the chromosome bar. The QTL positions are depicted as a red or blue horizontal bar underneath the population name. Black dots give the location of the confidence interval bordering markers on the population map. The dashed purple vertical line indicates the extend of the confidence interval. The line is given as dotted line, if the bordering markers are located further away on the consensus map than in the population map. The additive effect of the QTL (in number of crossover) is listed behind the population name. QTL and additive effect are given in blue if the effect is on the A parent and in red if the effect is on the B parent. Abbreviations: add = additive effect, XO = crossover trait.

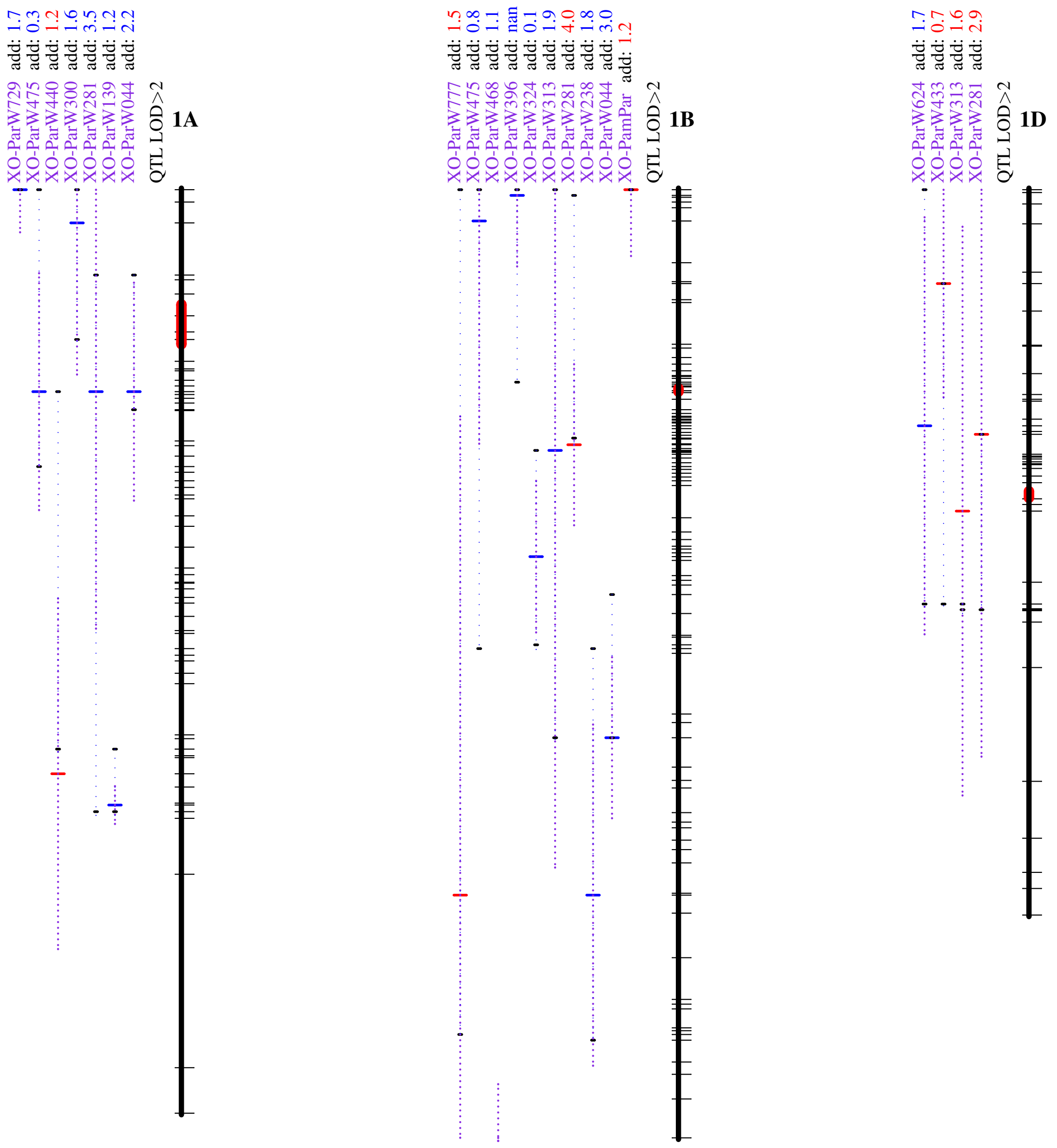

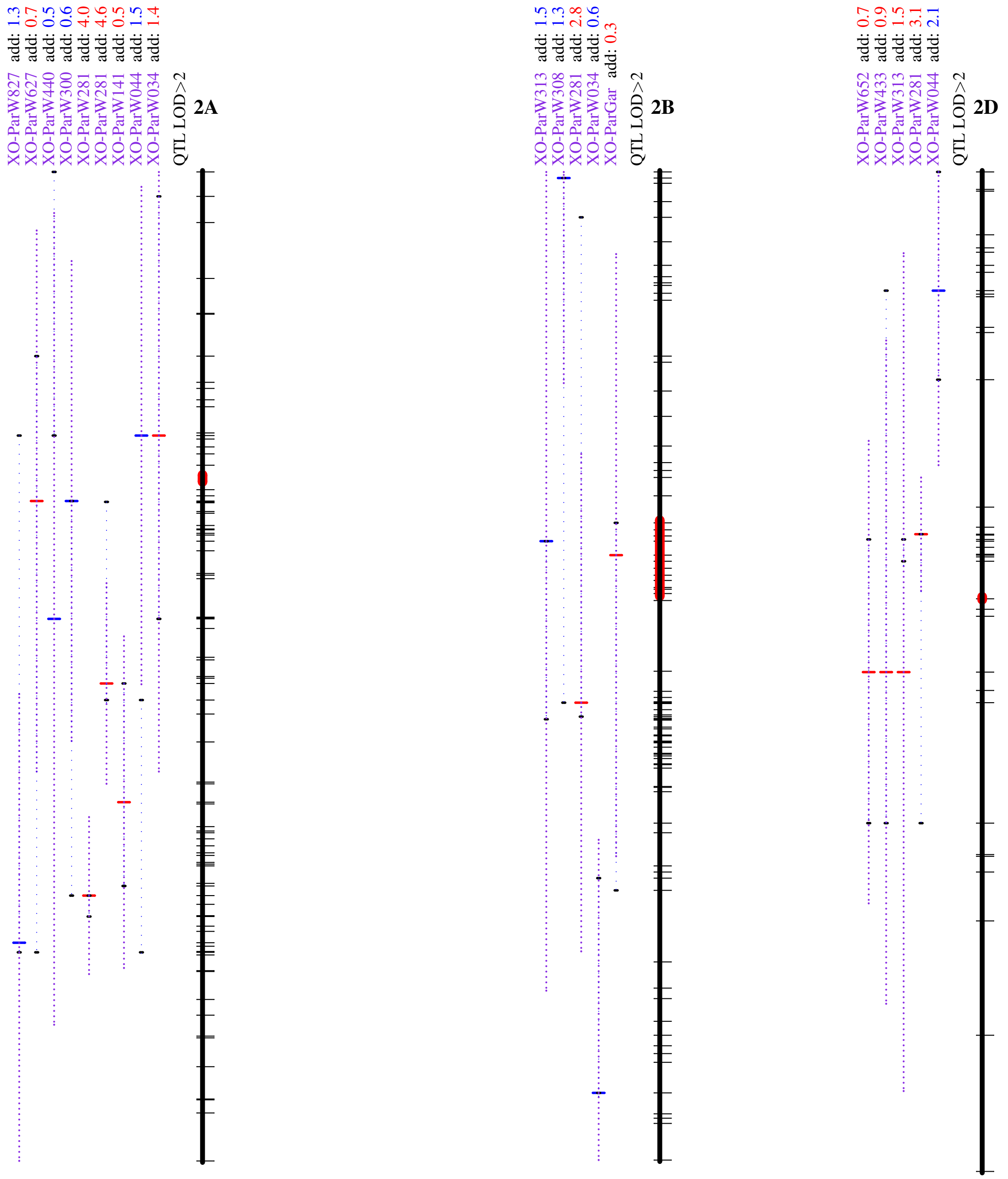
2A
QTL LOD>2
XO-ParW827 add: 1.3
XO-ParW627 add: 0.7
XO-ParW440 add: 0.5
XO-ParW300 add: 0.6
XO-ParW281 add: 4.0
XO-ParW281 add: 4.6
XO-ParW141 add: 0.5
XO-ParW044 add: 1.5
XO-ParW034 add: 1.4
2B
QTL LOD>2
XO-ParW313 add: 1.5
XO-ParW308 add: 1.3
XO-ParW281 add: 2.8
XO-ParW034 add: 0.6
XO-ParGar add: 0.3
2D
QTL LOD>2
XO-ParW652 add: 0.7
XO-ParW433 add: 0.9
XO-ParW313 add: 1.5
XO-ParW281 add: 3.1
XO-ParW044 add: 2.1

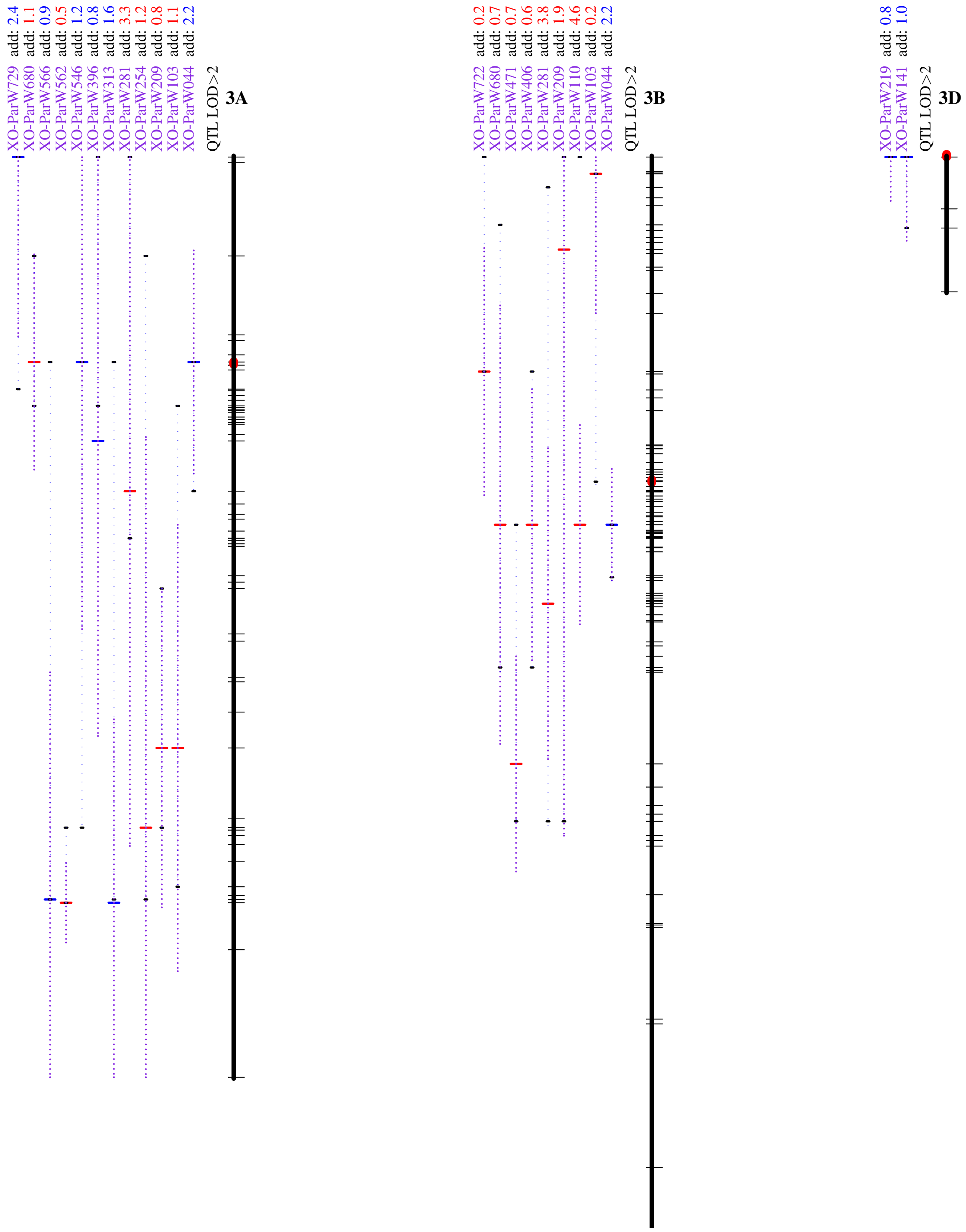
3A
QTL LOD>2
XO-ParW729 add: 2.4
XO-ParW680 add: 1.1
XO-ParW566 add: 0.9
XO-ParW562 add: 0.5
XO-ParW546 add: 1.2
XO-ParW396 add: 0.8
XO-ParW313 add: 1.6
XO-ParW281 add: 3.3
XO-ParW254 add: 1.2
XO-ParW209 add: 0.8
XO-ParW103 add: 1.1
XO-ParW044 add: 2.2
3B
QTL LOD>2
XO-ParW722 add: 0.2
XO-ParW680 add: 0.7
XO-ParW471 add: 0.7
XO-ParW406 add: 0.6
XO-ParW281 add: 3.8
XO-ParW209 add: 1.9
XO-ParW110 add: 4.6
XO-ParW103 add: 0.2
XO-ParW044 add: 2.2
3D
QTL LOD>2
XO-ParW219 add: 0.8
XO-ParW141 add: 1.0

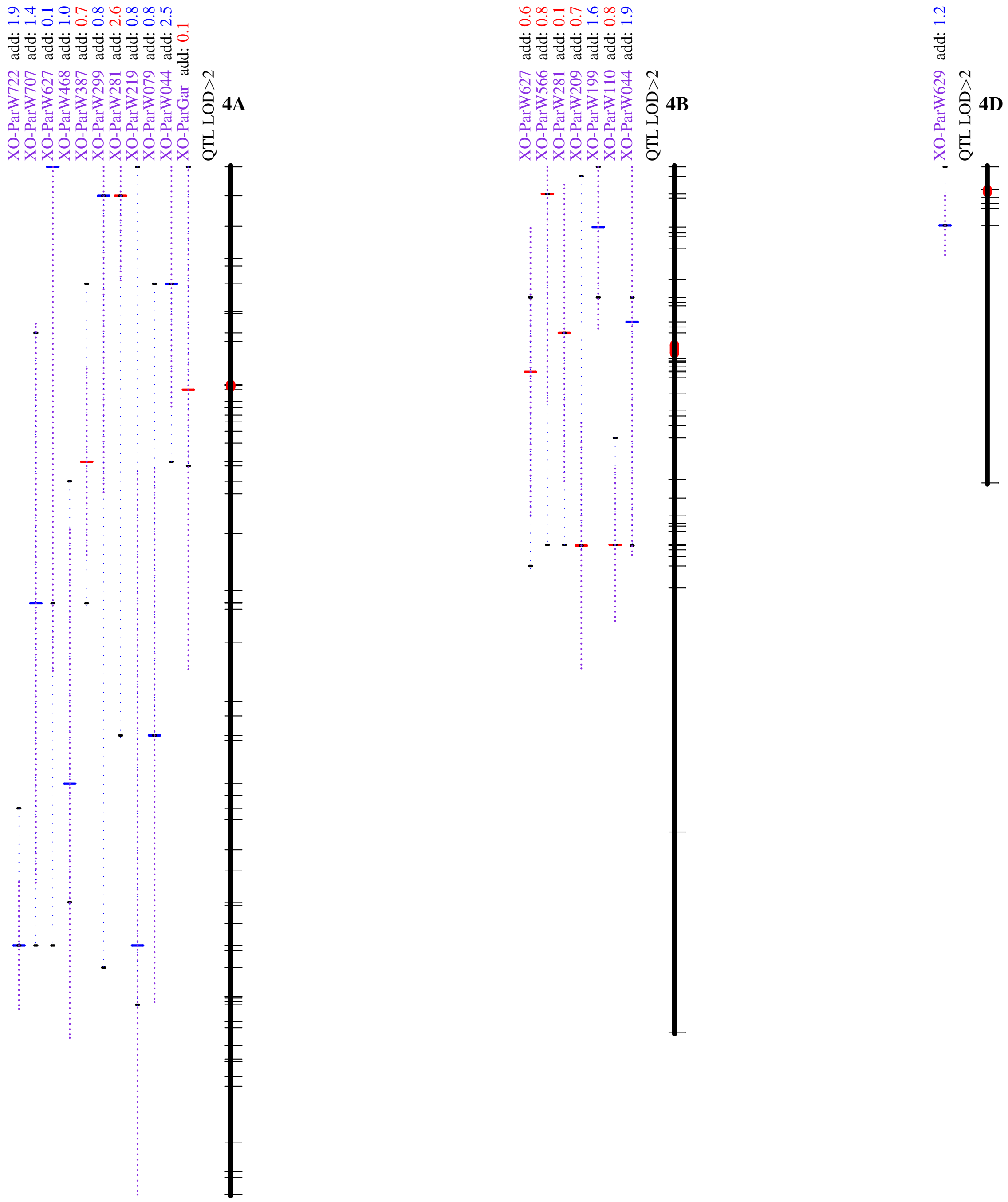
4A
QTL LOD>2
XO-ParW722 add: 1.9
XO-ParW707 add: 1.4
XO-ParW627 add: 0.1
XO-ParW468 add: 1.0
XO-ParW387 add: 0.7
XO-ParW299 add: 0.8
XO-ParW281 add: 2.6
XO-ParW219 add: 0.8
XO-ParW079 add: 0.8
XO-ParW044 add: 2.5
XO-ParGar add: 0.1
4B
QTL LOD>2
XO-ParW627 add: 0.6
XO-ParW566 add: 0.8
XO-ParW281 add: 0.1
XO-ParW209 add: 0.7
XO-ParW199 add: 1.6
XO-ParW110 add: 0.8
XO-ParW044 add: 1.9
4D
QTL LOD>2
XO-ParW629 add: 1.2

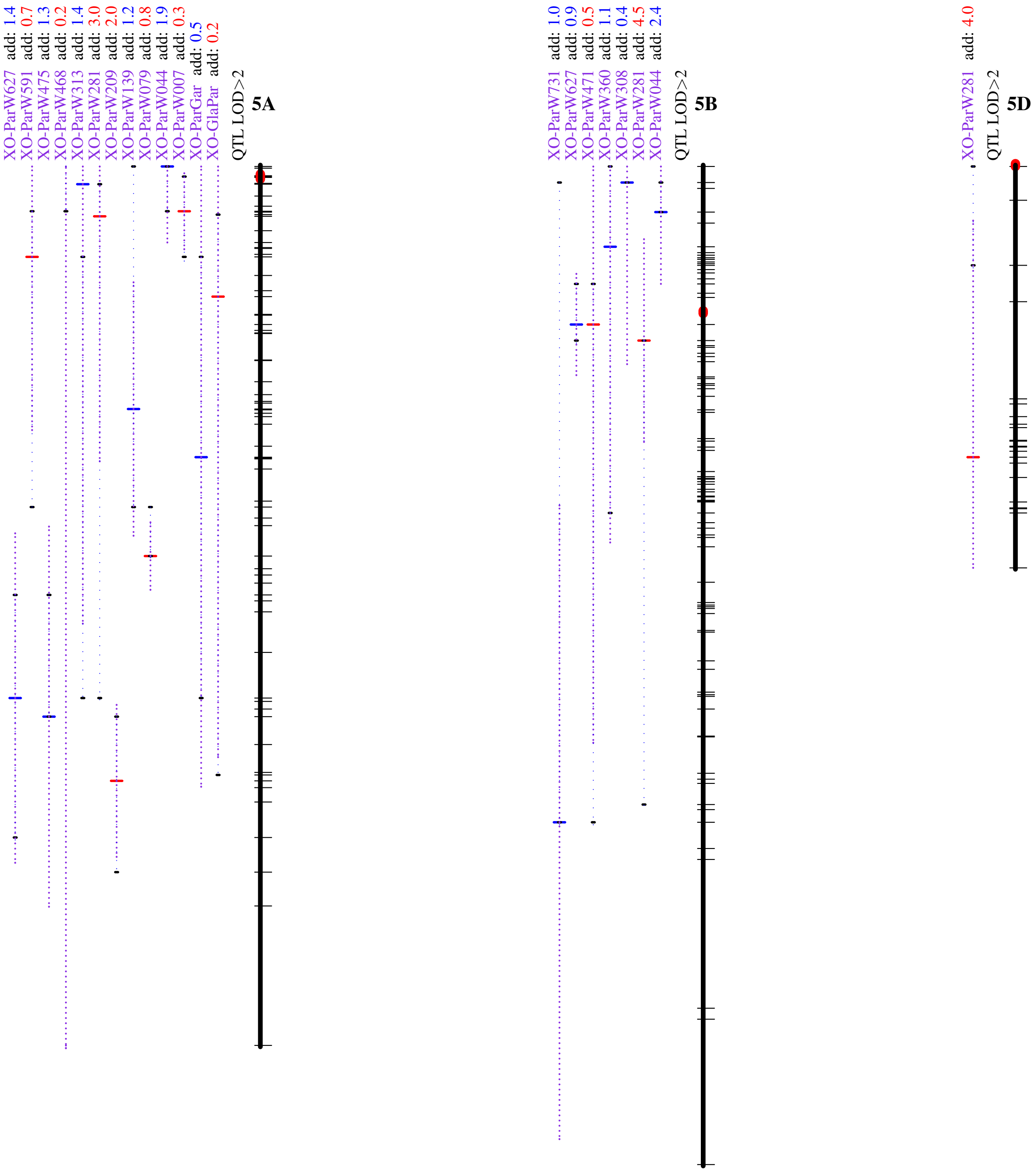
5A
QTL LOD>2
XO-ParW627 add: 1.4
XO-ParW591 add: 0.7
XO-ParW475 add: 1.3
XO-ParW468 add: 0.2
XO-ParW313 add: 1.4
XO-ParW281 add: 3.0
XO-ParW209 add: 2.0
XO-ParW139 add: 1.2
XO-ParW079 add: 0.8
XO-ParW044 add: 1.9
XO-ParW007 add: 0.3
XO-ParGar add: 0.5
XO-GlaPar add: 0.2
5B
QTL LOD>2
XO-ParW731 add: 1.0
XO-ParW627 add: 0.9
XO-ParW471 add: 0.5
XO-ParW360 add: 1.1
XO-ParW308 add: 0.4
XO-ParW281 add: 4.5
XO-ParW044 add: 2.4
5D
QTL LOD>2
XO-ParW281 add: 4.0

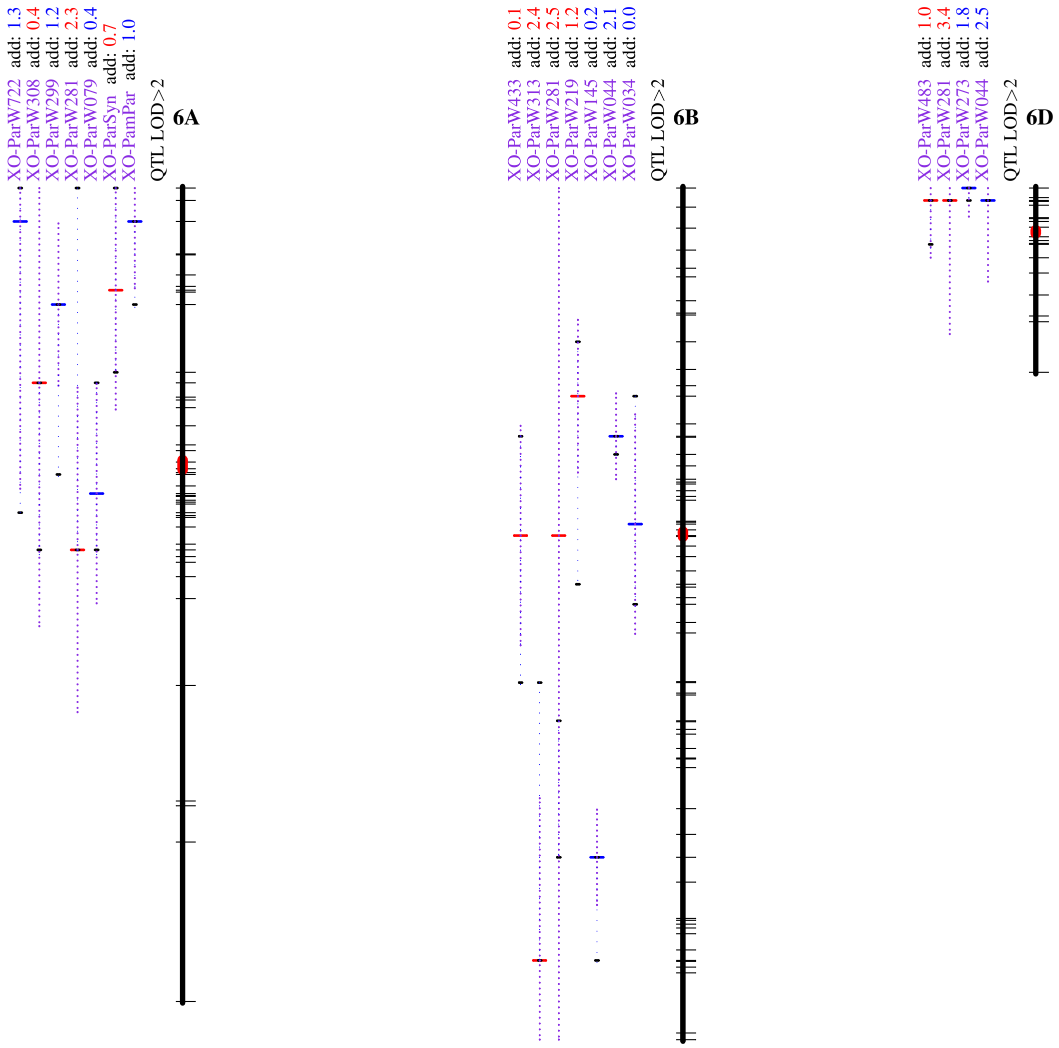
6A
QTL LOD>2
XO-ParW722 add: 1.3
XO-ParW308 add: 0.4
XO-ParW299 add: 1.2
XO-ParW281 add: 2.3
XO-ParW079 add: 0.4
XO-ParSyn add: 0.7
XO-PamPar add: 1.0
6B
QTL LOD>2
XO-ParW433 add: 0.1
XO-ParW313 add: 2.4
XO-ParW281 add: 2.5
XO-ParW219 add: 1.2
XO-ParW145 add: 0.2
XO-ParW044 add: 2.1
XO-ParW034 add: 0.0
6D
QTL LOD>2
XO-ParW483 add: 1.0
XO-ParW281 add: 3.4
XO-ParW273 add: 1.8
XO-ParW044 add: 2.5

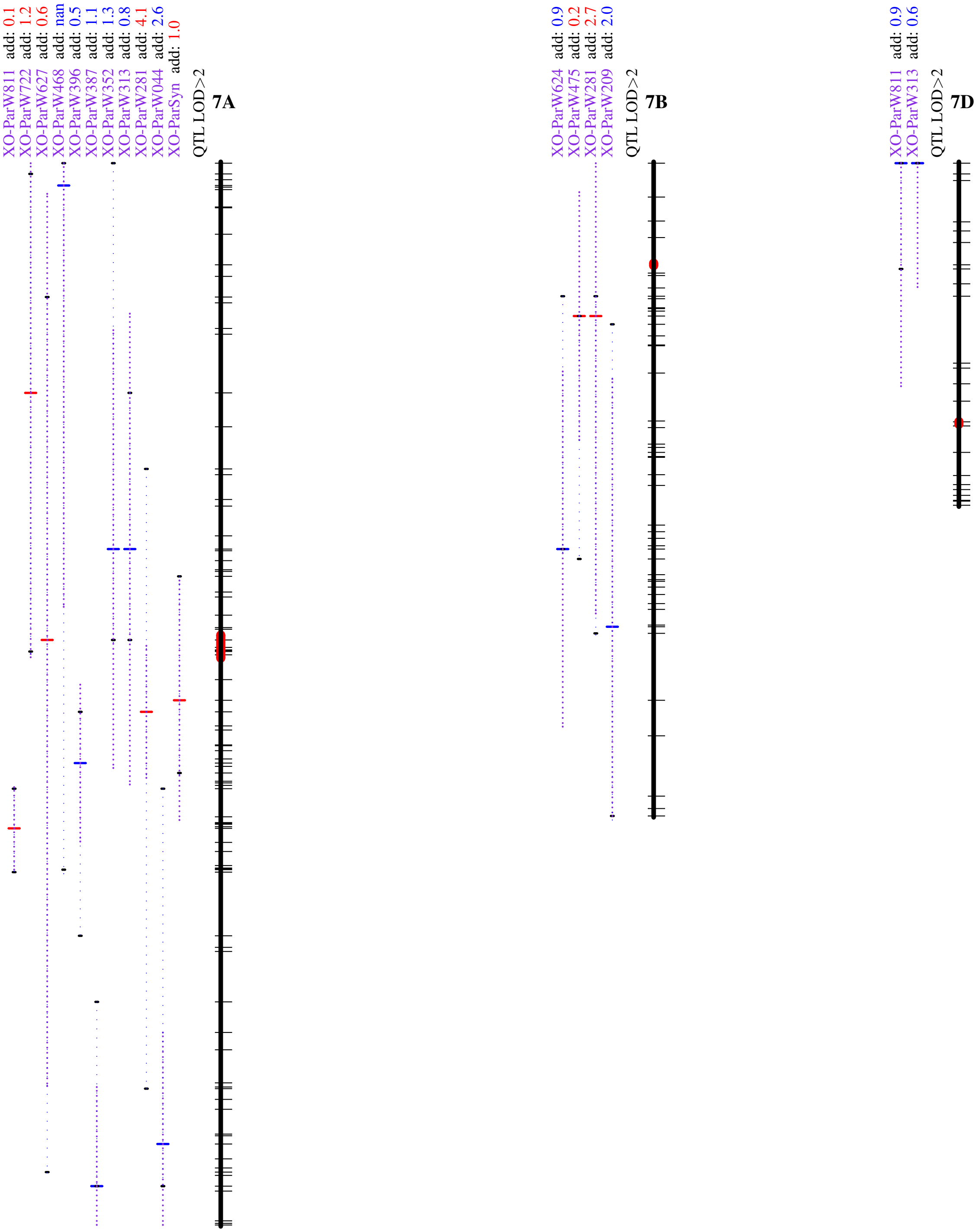
XO-ParW811 add: 0.1
XO-ParW722 add: 1.2
XO-ParW627 add: 0.6
XO-ParW468 add: nan
XO-ParW396 add: 0.5
XO-ParW387 add: 1.1
XO-ParW352 add: 1.3
XO-ParW313 add: 0.8
XO-ParW281 add: 4.1
XO-ParW044 add: 2.6
XO-ParSyn add: 1.0
QTL LOD>2
7A
XO-ParW624 add: 0.9
XO-ParW475 add: 0.2
XO-ParW281 add: 2.7
XO-ParW209 add: 2.0
QTL LOD>2
7B
XO-ParW811 add: 0.9
XO-ParW313 add: 0.6
QTL LOD>2
7D

Main Figure 1 **List of Populations** .
